# Supplementary material for: Alternatives in Education—Evaluation of Rat Simulators in Laboratory Animal Training Courses from Participants’ Perspective
Source: Animals (Basel). 2021 Dec 5;11(12):3462. doi: 10.3390/ani11123462 (PMC8698197; doi:10.3390/ani11123462)
Supplement: Supplementary file 1 [file animals-11-03462-s001.zip › Table S1_Animals_revised_27.11.21.pdf]

Table S1. Questionnaire for course participants in laboratory animal science courses
Original PDF document of the two-part paper-based questionnaire on the practical evaluation of currently available rat simulators distributed in LAS (laboratory animal science) courses. Simulator product names were anonymized via codes (Rat simulator A - E) and illustrations were removed. For illustrations of the simulators, see Table 1.

Welcome to „SimulRATor“!

We invite you to take part in our survey on simulators in laboratory animal science.

We are a team of scientists of the Institutes of Veterinary Anatomy, of Animal Welfare, Animal Behavior and Laboratory Animal Science and of Veterinary Epidemiology and Biometry at the Department of Veterinary Medicine of the Freie Universität Berlin and **are evaluating all currently commercially available rat and mouse simulators in laboratory animal training courses for a research project.**

Since you as a participant can evaluate best, how good the training on the simulator and the preparation for the practical exercises on the live animal is, we would kindly ask you for **your personal feedback on the strengths and weaknesses of the simulators** on which you have trained in the course.

Thank you very much for your participation!

The SimulRATor-Team

Further information can be found at www.simulrator.de or write to kontakt@simulrator.de.

Part 1 of 2 Rat Simulator A

ID: ___________________

← Please remember your ID for the 2nd part. Thank you!

Privacy Notice: Your participation in the survey is voluntary. The survey data is anonymously stored and managed. Under no circumstances will data be passed on to third parties. We collect no personal information from you. No conclusions on your participation are possible. Only the people involved in the research project have access to the information provided by you within the questionnaire and are obliged to maintain secrecy. The information provided is used for research purposes only. You can cancel the survey at any time without giving reasons.

1. How well were you able to apply the procedural techniques on the Rat Simulator A?

Techniques	Extremely good 1	Quite good 2	Slightly good 3	Slightly bad 4	Quite bad 5	Extremely bad 6	Technique was not trained.
Handling - transfer from cage to cage	o	o	o	o	o	o	o
Restraint - scruffing	o	o	o	o	o	o	o
Restraint - over the shoulder grip	o	o	o	o	o	o	o
Restraint - middle shoulder grip	o	o	o	o	o	o	o
Restraint - under the shoulder grip	o	o	o	o	o	o	o
Administration by oral gavage	o	o	o	o	o	o	o
Intravenous administration via lateral tail vein	o	o	o	o	o	o	o
Blood sampling from lateral tail vein	o	o	o	o	o	o	o
Other: ______________________________	o	o	o	o	o	o	o
______________________________	o	o	o	o	o	o	o

2. How well do the following statements about the training with the Rat Simulator A apply?

	Completely applies 1	Largely applies 2	Slightly applies 3	Slightly does not apply 4	Largely does not apply 5	Does not apply at all 6
The number of participants per Rat Simulator A was appropriate.	o	o	o	o	o	o
The training duration on the Rat Simulator A was sufficient.	o	o	o	o	o	o
The Rat Simulator A provides satisfactory training for the techniques.	o	o	o	o	o	o
By training with the Rat Simulator A, I feel better prepared for course training on the live rat.	o	o	o	o	o	o

3. If there were material related difficulties using the Rat Simulator A, please give us a brief description of these.

Technique: ____

Description of the complication: ____

____

____

Technique: ____

Description of the complication: ____

____

____

Do you have any recommendations, praise or criticism? We highly appreciate your feedback!

Thank you very much for your participation!

The SimulRATor-Team kontakt@simulrator.de

DEUTSCHES ZENTRUM ZUM SCHUTZ VON VERSUCHSTIEREN

Freie Universität Berlin

Welcome to „SimulRATor“!

We invite you to take part in our survey on simulators in laboratory animal science.

We are a team of scientists of the Institutes of Veterinary Anatomy, of Animal Welfare, Animal Behavior and Laboratory Animal Science and of Veterinary Epidemiology and Biometry at the Department of Veterinary Medicine of the Freie Universität Berlin and **are evaluating all currently commercially available rat and mouse simulators in laboratory animal training courses for a research project.**

Since you as a participant can evaluate best, how good the training on the simulator and the preparation for the practical exercises on the live animal is, we would kindly ask you for **your personal feedback on the strengths and weaknesses of the simulators** on which you have trained in the course.

Thank you very much for your participation!

The SimulRATor-Team

Further information can be found at www.simulrator.de or write to kontakt@simulrator.de.

Part 2 of 2 Rat Simulator A

ID: ________________

Privacy Notice: Your participation in the survey is voluntary. The survey data is anonymously stored and managed. Under no circumstances will data be passed on to third parties. We collect no personal information from you. No conclusions on your participation are possible. Only the people involved in the research project have access to the information provided by you within the questionnaire and are obliged to maintain secrecy. The information provided is used for research purposes only. You can cancel the survey at any time without giving reasons.

1. How well were you able to manage handling, restraint, and ear punching on the live rat?

**Techniques**	Extremely good **1**	Quite good **2**	Slightly good **3**	Slightly bad **4**	Quite bad **5**	Extremely bad **6**	Technique was not trained.
Handling - transfer from cage to cage	o	o	o	o	o	o	o
Restraint - scruffing	o	o	o	o	o	o	o
Restraint - over the shoulder grip	o	o	o	o	o	o	o
Restraint - middle shoulder grip	o	o	o	o	o	o	o
Restraint – under the shoulder grip	o	o	o	o	o	o	o
Ear punching	o	o	o	o	o	o	o
Other: ________________________________	o	o	o	o	o	o	o
________________________________	o	o	o	o	o	o	o

2. How well were you able to manage the following procedural techniques on the live rat?

Please also tick the box in the last column if the rat was anesthetized for the method.

**Techniques**	Extremely good **1**	Quite good **2**	Slightly good **3**	Slightly bad **4**	Quite bad **5**	Extremely bad **6**	Not trained.	Rat in anesthesia.
Feeding the rat	o	o	o	o	o	o	o	☐
Administration by oral gavage	o	o	o	o	o	o	o	☐
Subcutaneous administration under skin over the neck	o	o	o	o	o	o	o	☐
Subcutaneous administration under skin over the flank	o	o	o	o	o	o	o	☐
Intramuscular administration	o	o	o	o	o	o	o	☐
Intraperitoneal administration	o	o	o	o	o	o	o	☐
Intravenouse administration via dorsal penis vein	o	o	o	o	o	o	o	☐
Intravenous administration via lateral tail vein	o	o	o	o	o	o	o	☐
Blood sampling from sublingual vein	o	o	o	o	o	o	o	☐
Blood sampling from retro-orbital plexus	o	o	o	o	o	o	o	☐
Blood sampling from saphenous vein	o	o	o	o	o	o	o	☐
Blood sampling from lateral tail vein	o	o	o	o	o	o	o	☐
Blood sampling from heart	o	o	o	o	o	o	o	☐
Other: ________________________________	o	o	o	o	o	o	o	☐
________________________________	o	o	o	o	o	o	o	☐

3. Which <u>3 techniques</u> on the live rat are in your opinion particularly demanding for the performer?

Please name 3 techniques from 1st and 2nd question. Please specify the techniques completely.

1. ____

2. ____

3. ____

4. Please select from the list below the 5 techniques on the live rat, for which you consider a preparatory training on simulators to be particularly useful.

Max. 5 techniques can be chosen.

- ☐ Handling - transfer from cage to cage
- ☐ Restraint (different techniques)
- ☐ Ear punching
- ☐ Feeding the rat
- ☐ Administration by oral gavage
- ☐ Subcutaneous administration under skin over the neck
- ☐ Subcutaneous administration under skin over the flank
- ☐ Intramuscular administration
- ☐ Intraperitoneal administration
- ☐ Intravenous administration via dorsal penis vein
- ☐ Intravenous administration via lateral tail vein
- ☐ Blood sampling from sublingual vein
- ☐ Blood sampling from retro-orbital plexus
- ☐ Blood sampling from saphenous vein
- ☐ Blood sampling from lateral tail vein
- ☐ Blood sampling from heart

5. Would you also like to have a simulator training for other methods on the rat?

If so, please describe which technique(s) is/are important to you.

Your personal feedback about the Rat Simulator A

6. How realistic are the following features of the Rat Simulator A in comparison to a live rat?

**Features**	**Extremely realistic** **1**	**Quite realistic** **2**	**Slightly realistic** **3**	**Slightly unrealistic** **4**	**Quite unrealistic** **5**	**Extremely unrealistic** **6**
General appearance	○	○	○	○	○	○
Haptics	○	○	○	○	○	○
Movability of skin over neck	○	○	○	○	○	○
Movability of skin over flank	○	○	○	○	○	○
Consistency of skin surface	○	○	○	○	○	○
Body height	○	○	○	○	○	○
Body weight	○	○	○	○	○	○
Body shape	○	○	○	○	○	○
Proportions	○	○	○	○	○	○

7. How realistic was the training with the Rat Simulator A in comparison to a live rat for you?

**Techniques**	**Extremely realistic** **1**	**Quite realistic** **2**	**Slightly realistic** **3**	**Slightly unrealistic** **4**	**Quite unrealistic** **5**	**Extremely unrealistic** **6**	**Technique was not trained.**
Handling - transfer from cage to cage	○	○	○	○	○	○	○
Restraint - scruffing	○	○	○	○	○	○	○
Restraint - over the shoulder grip	○	○	○	○	○	○	○
Restraint - middle shoulder grip	○	○	○	○	○	○	○
Restraint - under the shoulder grip	○	○	○	○	○	○	○
Administration by oral gavage	○	○	○	○	○	○	○
Intravenous administration via lateral tail vein	○	○	○	○	○	○	○
Blood sampling from lateral tail vein	○	○	○	○	○	○	○
Other: ________________________________	○	○	○	○	○	○	○
________________________________	○	○	○	○	○	○	○

8. How well do the following statements about the training with the Rat Simulator A apply?

	**Completely applies** **1**	**Largely applies** **2**	**Slightly applies** **3**	**Slightly does not apply** **4**	**Largely does not apply** **5**	**Does not apply at all** **6**
I felt more secure in performing **handling und restraint** on the live rat.	○	○	○	○	○	○
I felt more secure in performing **procedural techniques** on the live rat.	○	○	○	○	○	○

9. How well did the Rat Simulator A prepare you for the entire course training on the live rat?

Extremely good	Quite good	Slightly good	Slightly bad	Quite bad	Extremely bad
1	2	3	4	5	6
○	○	○	○	○	○

10. How well did the Rat Simulator A prepare you for the following techniques in the course training on the live rat?

Techniques	Extremely good 1	Quite good 2	Slightly good 3	Slightly bad 4	Quite bad 5	Extremely bad 6	Technique was not trained.
Handling - transfer from cage to cage	○	○	○	○	○	○	○
Restraint - scruffing	○	○	○	○	○	○	○
Restraint - over the shoulder grip	○	○	○	○	○	○	○
Restraint - middle shoulder grip	○	○	○	○	○	○	○
Restraint - under the shoulder grip	○	○	○	○	○	○	○
Administration by oral gavage	○	○	○	○	○	○	○
Intravenous administration via lateral tail vein	○	○	○	○	○	○	○
Blood sampling from lateral tail vein	○	○	○	○	○	○	○
Other: ________________________________	○	○	○	○	○	○	○
________________________________	○	○	○	○	○	○	○

11. What did you particularly like about the Rat Simulator A?

12. What did you not like about the Rat Simulator A?

13. What would you like to improve about the Rat Simulator A?

14. Which species are you expecting to work with for scientific reasons in the next 6 months after you have passed the course?
Multiple answers for species are possible.

- ☐ Rat
- ☐ Mouse
- ☐ Rabbit
- ☐ Guinea pig
- ☐ Hamster
- ☐ Pig
- ☐ Sheep
- ☐ Goat
- ☐ Cow
- ☐ Horse
- ☐ Dog
- ☐ Cat
- ☐ Other_____________________________
- ☐ None
- ☐ I cannot judge.

15. How old are you?

________ years

16. What is your gender?

- ☐ Male
- ☐ Female
- ☐ Other
- ☐ No answer

17. Please mark your <u>highest</u> educational degree you have achieved.
Only one answer is possible.

- ☐ (Still) no qualification (yet)
- ☐ Apprenticeship/Vocational training
- ☐ Technical college qualification
- ☐ University of Applied Sciences Degree
- ☐ Bachelor's Degree
- ☐ Master's Degree, diploma or state examination
- ☐ Ph.D. /Doctoral degree
- ☐ Other: ____

18. Please complete the sentence below.
Only one answer is possible.

I am participating in the course as …

- ☐ …an academic employee.
- ☐ …a technical assistant.
- ☐ …an apprentice.
- ☐ …a student.
- ☐ …other: ____.

19. What is your discipline?
Only one answer is possible.

- ☐ Human Medicine
- ☐ Veterinary Medicine
- ☐ Dental Medicine
- ☐ Pharmacology
- ☐ Biology
- ☐ Chemistry
- ☐ Physics
- ☐ Biotechnology
- ☐ Nutritional Science
- ☐ Other: ____

20. Had you already had experience …
Only one answer is possible.

…in handling <u>rats</u> before the course?

- ☐ Yes, a lot of.
- ☐ Yes, a little bit.
- ☐ No, not at all.

21. Had you already had experience …
Only one answer is possible.

…in handling <u>mice</u> before the course?

- ☐ Yes, a lot of.
- ☐ Yes, a little bit.
- ☐ No, not at all.

22. Had you already worked with other simulator(s) before the course?
Multiple answers for simulators are possible. Please describe the model in brief.

- ☐ Yes, …
  - ☐ …with rat simulators: Model: ____
  - ☐ …with mouse simulators: Model: ______________________________ __________
  - ☐ …with other simulators: Model: ____
- ☐ No.

Do you have any recommendations, praise or criticism? We highly appreciate your feedback!

Thank you very much for your participation!

The SimulRATor-Team

web: www.simulator.de mail: kontakt@simulrator.de

Welcome to „SimulRATor“!

We invite you to take part in our survey on simulators in laboratory animal science.

We are a team of scientists of the Institutes of Veterinary Anatomy, of Animal Welfare, Animal Behavior and Laboratory Animal Science and of Veterinary Epidemiology and Biometry at the Department of Veterinary Medicine of the Freie Universität Berlin and **are evaluating all currently commercially available rat and mouse simulators in laboratory animal training courses for a research project.**

Since you as a participant can evaluate best, how good the training on the simulator and the preparation for the practical exercises on the live animal is, we would kindly ask you for **your personal feedback on the strengths and weaknesses of the simulators** on which you have trained in the course.

Thank you very much for your participation!

The SimulRATor-Team

Further information can be found at www.simulrator.de or write to kontakt@simulrator.de.

Part 1 of 2 Rat Simulator B

ID: ________________

← **Please remember your ID for the 2nd part.**

Thank you!

Privacy Notice: Your participation in the survey is voluntary. The survey data is anonymously stored and managed. Under no circumstances will data be passed on to third parties. We collect no personal information from you. No conclusions on your participation are possible. Only the people involved in the research project have access to the information provided by you within the questionnaire and are obliged to maintain secrecy. The information provided is used for research purposes only. You can cancel the survey at any time without giving reasons.

1. How well were you able to apply the procedural techniques on the Rat Simulator B?

Techniques	Extremely good 1	Quite good 2	Slightly good 3	Slightly bad 4	Quite bad 5	Extremely bad 6	Technique was not trained.
Handling - transfer from cage to cage	o	o	o	o	o	o	o
Restraint - scruffing	o	o	o	o	o	o	o
Restraint - over the shoulder grip	o	o	o	o	o	o	o
Restraint - middle shoulder grip	o	o	o	o	o	o	o
Restraint - under the shoulder grip	o	o	o	o	o	o	o
Administration by oral gavage	o	o	o	o	o	o	o
Intravenous administration via lateral tail vein	o	o	o	o	o	o	o
Blood sampling from lateral tail vein	o	o	o	o	o	o	o
Other: ________________________________	o	o	o	o	o	o	o
________________________________	o	o	o	o	o	o	o

2. How well do the following statements about the training with the Rat Simulator B apply?

	Completely applies 1	Largely applies 2	Slightly applies 3	Slightly does not apply 4	Largely does not apply 5	Does not apply at all 6
The number of participants per Rat Simulator B was appropriate.	o	o	o	o	o	o
The training duration on the Rat Simulator B was sufficient.	o	o	o	o	o	o
The Rat Simulator B provides satisfactory training for the techniques.	o	o	o	o	o	o
By training with the Rat Simulator B, I feel better prepared for course training on the live rat.	o	o	o	o	o	o

3. If there were material related difficulties using the Rat Simulator B, please give us a brief description of these.

Technique: ____

Description of the complication: ____

____

____

Technique: ____

Description of the complication: ____

____

____

Do you have any recommendations, praise or criticism? We highly appreciate your feedback!

Thank you very much for your participation!

The SimulRATor-Team kontakt@simulrator.de

DEUTSCHES ZENTRUM ZUM SCHUTZ VON VERSUCHSTIEREN

Freie Universität Berlin

Welcome to „SimulRATor“!

We invite you to take part in our survey on simulators in laboratory animal science.

We are a team of scientists of the Institutes of Veterinary Anatomy, of Animal Welfare, Animal Behavior and Laboratory Animal Science and of Veterinary Epidemiology and Biometry at the Department of Veterinary Medicine of the Freie Universität Berlin and **are evaluating all currently commercially available rat and mouse simulators in laboratory animal training courses for a research project.**

Since you as a participant can evaluate best, how good the training on the simulator and the preparation for the practical exercises on the live animal is, we would kindly ask you for **your personal feedback on the strengths and weaknesses of the simulators** on which you have trained in the course.

Thank you very much for your participation!

The SimulRATor-Team

Further information can be found at www.simulrator.de or write to kontakt@simulrator.de.

Part 2 of 2 Rat Simulator B

ID: ___________________

Privacy Notice: Your participation in the survey is voluntary. The survey data is anonymously stored and managed. Under no circumstances will data be passed on to third parties. We collect no personal information from you. No conclusions on your participation are possible. Only the people involved in the research project have access to the information provided by you within the questionnaire and are obliged to maintain secrecy. The information provided is used for research purposes only. You can cancel the survey at any time without giving reasons.

1. How well were you able to manage handling, restraint, and ear punching on the live rat?

**Techniques**	**Extremely good 1**	**Quite good 2**	**Slightly good 3**	**Slightly bad 4**	**Quite bad 5**	**Extremely bad 6**	**Technique was not trained.**
Handling - transfer from cage to cage	o	o	o	o	o	o	o
Restraint - scruffing	o	o	o	o	o	o	o
Restraint - over the shoulder grip	o	o	o	o	o	o	o
Restraint - middle shoulder grip	o	o	o	o	o	o	o
Restraint – under the shoulder grip	o	o	o	o	o	o	o
Ear punching	o	o	o	o	o	o	o
Other: ________________________________	o	o	o	o	o	o	o
________________________________	o	o	o	o	o	o	o

2. How well were you able to manage the following procedural techniques on the live rat?

Please also tick the box in the last column if the rat was anesthetized for the method.

**Techniques**	**Extremely good 1**	**Quite good 2**	**Slightly good 3**	**Slightly bad 4**	**Quite bad 5**	**Extremely bad 6**	**Not trained.**	**Rat in anesthesia.**
Feeding the rat	o	o	o	o	o	o	o	☐
Administration by oral gavage	o	o	o	o	o	o	o	☐
Subcutaneous administration under skin over the neck	o	o	o	o	o	o	o	☐
Subcutaneous administration under skin over the flank	o	o	o	o	o	o	o	☐
Intramuscular administration	o	o	o	o	o	o	o	☐
Intraperitoneal administration	o	o	o	o	o	o	o	☐
Intravenouse administration via dorsal penis vein	o	o	o	o	o	o	o	☐
Intravenous administration via lateral tail vein	o	o	o	o	o	o	o	☐
Blood sampling from sublingual vein	o	o	o	o	o	o	o	☐
Blood sampling from retro-orbital plexus	o	o	o	o	o	o	o	☐
Blood sampling from saphenous vein	o	o	o	o	o	o	o	☐
Blood sampling from lateral tail vein	o	o	o	o	o	o	o	☐
Blood sampling from heart	o	o	o	o	o	o	o	☐
Other: ________________________________	o	o	o	o	o	o	o	☐
________________________________	o	o	o	o	o	o	o	☐

3. Which 3 techniques on the live rat are in your opinion particularly demanding for the performer?

Please name 3 techniques from 1st and 2nd question. Please specify the techniques completely.

1. ____

2. ____

3. ____

4. Please select from the list below the 5 techniques on the live rat, for which you consider a preparatory training on simulators to be particularly useful.

Max. 5 techniques can be chosen.

- ☐ Handling - transfer from cage to cage
- ☐ Restraint (different techniques)
- ☐ Ear punching
- ☐ Feeding the rat
- ☐ Administration by oral gavage
- ☐ Subcutaneous administration under skin over the neck
- ☐ Subcutaneous administration under skin over the flank
- ☐ Intramuscular administration
- ☐ Intraperitoneal administration
- ☐ Intravenous administration via dorsal penis vein
- ☐ Intravenous administration via lateral tail vein
- ☐ Blood sampling from sublingual vein
- ☐ Blood sampling from retro-orbital plexus
- ☐ Blood sampling from saphenous vein
- ☐ Blood sampling from lateral tail vein
- ☐ Blood sampling from heart

5. Would you also like to have a simulator training for other methods on the rat?

If so, please describe which technique(s) is/are important to you.

Your personal feedback about the Rat Simulator B

6. How realistic are the following features of the Rat Simulator B in comparison to a live rat?

Features	Extremely realistic 1	Quite realistic 2	Slightly realistic 3	Slightly <u>un</u>realistic 4	Quite <u>un</u>realistic 5	Extremely <u>un</u>realistic 6
General appearance	o	o	o	o	o	o
Haptic	o	o	o	o	o	o
Movability of skin over neck	o	o	o	o	o	o
Movability of skin over flank	o	o	o	o	o	o
Consistency of skin surface	o	o	o	o	o	o
Body height	o	o	o	o	o	o
Body weight	o	o	o	o	o	o
Body shape	o	o	o	o	o	o
Proportions	o	o	o	o	o	o

7. How realistic was the training with the Rat Simulator B in comparison to a live rat for you?

Techniques	Extremely realistic 1	Quite realistic 2	Slightly realistic 3	Slightly <u>un</u>realistic 4	Quite <u>un</u>realistic 5	Extremely <u>un</u>realistic 6	Technique was not trained.
Handling - transfer from cage to cage	o	o	o	o	o	o	o
Restraint - scruffing	o	o	o	o	o	o	o
Restraint - over the shoulder grip	o	o	o	o	o	o	o
Restraint - middle shoulder grip	o	o	o	o	o	o	o
Restraint - under the shoulder grip	o	o	o	o	o	o	o
Administration by oral gavage	o	o	o	o	o	o	o
Intravenous administration via lateral tail vein	o	o	o	o	o	o	o
Blood sampling from lateral tail vein	o	o	o	o	o	o	o
Other: ______________________________	o	o	o	o	o	o	o
______________________________	o	o	o	o	o	o	o

8. How well do the following statements about the training with the Rat Simulator B apply?

	Completely applies 1	Largely applies 2	Slightly applies 3	Slightly does <u>not</u> apply 4	Largely does <u>not</u> apply 5	Does <u>not</u> apply at all 6
I felt more secure in performing **handling und restraint** on the live rat.	o	o	o	o	o	o
I felt more secure in performing **procedural techniques** on the live rat.	o	o	o	o	o	o

9. How well did the Rat Simulator B prepare you for the entire course training on the live rat?

Extremely good	Quite good	Slightly good	Slightly bad	Quite bad	Extremely bad
1	2	3	4	5	6
○	○	○	○	○	○

10. How well did the Rat Simulator B prepare you for the following techniques in the course training on the live rat?

Techniques	Extremely good 1	Quite good 2	Slightly good 3	Slightly bad 4	Quite bad 5	Extremely bad 6	Technique was not trained.
Handling - transfer from cage to cage	○	○	○	○	○	○	○
Restraint - scruffing	○	○	○	○	○	○	○
Restraint - over the shoulder grip	○	○	○	○	○	○	○
Restraint - middle shoulder grip	○	○	○	○	○	○	○
Restraint - under the shoulder grip	○	○	○	○	○	○	○
Administration by oral gavage	○	○	○	○	○	○	○
Intravenous administration via lateral tail vein	○	○	○	○	○	○	○
Blood sampling from lateral tail vein	○	○	○	○	○	○	○
Other: ___________________________________	○	○	○	○	○	○	○
___________________________________	○	○	○	○	○	○	○

11. What did you particularly like about the Rat Simulator B?

12. What did you not like about the Rat Simulator B?

13. What would you like to improve about the Rat Simulator B?

14. Which species are you expecting to work with for scientific reasons in the next 6 months after you have passed the course?
Multiple answers for species are possible.

- □ Rat
- □ Mouse
- □ Rabbit
- □ Guinea pig
- □ Hamster
- □ Pig
- □ Sheep
- □ Goat
- □ Cow
- □ Horse
- □ Dog
- □ Cat
- □ Other______________________________
- □ None
- □ I cannot judge.

15. How old are you?

_________ years

Please turn over.

16. What is your gender?

- □ Male
- □ Female
- □ Other
- □ No answer

17. Please mark your highest educational degree you have achieved.
Only one answer is possible.

- □ (Still) no qualification (yet)
- □ Apprenticeship/Vocational training
- □ Technical college qualification
- □ University of Applied Sciences Degree
- □ Bachelor's Degree
- □ Master's Degree, diploma or state examination
- □ Ph.D. /Doctoral degree
- □ Other: ____

18. Please complete the sentence below.
Only one answer is possible.

I am participating in the course as …

- □ …an academic employee.
- □ …a technical assistant.
- □ …an apprentice.
- □ …a student.
- □ …other: ____.

19. What is your discipline?
Only one answer is possible.

- □ Human Medicine
- □ Veterinary Medicine
- □ Dental Medicine
- □ Pharmacology
- □ Biology
- □ Chemistry
- □ Physics
- □ Biotechnology
- □ Nutritional Science
- □ Other: ____

20. Had you already had experience …
Only one answer is possible.

…in handling rats before the course?

- □ Yes, a lot of.
- □ Yes, a little bit.
- □ No, not at all.

21. Had you already had experience …
Only one answer is possible.

…in handling mice before the course?

- □ Yes, a lot of.
- □ Yes, a little bit.
- □ No, not at all.

22. Had you already worked with other simulator(s) before the course?
Multiple answers for simulators are possible. Please describe the model in brief.

- □ Yes, …
  - □ …with rat simulators: Model: ____
  - □ …with mouse simulators: Model: _______________________________ __________
  - □ …with other simulators: Model: ____
- □ No.

Do you have any recommendations, praise or criticism? We highly appreciate your feedback!

Thank you very much for your participation!

The SimulRATor-Team

web: www.simulator.de mail: kontakt@simulrator.de

DEUTSCHES ZENTRUM ZUM SCHUTZ VON VERSUCHSTIEREN

Freie Universität Berlin

Welcome to „SimulRATor“!

We invite you to take part in our survey on simulators in laboratory animal science.

We are a team of scientists of the Institutes of Veterinary Anatomy, of Animal Welfare, Animal Behavior and Laboratory Animal Science and of Veterinary Epidemiology and Biometry at the Department of Veterinary Medicine of the Freie Universität Berlin and **are evaluating all currently commercially available rat and mouse simulators in laboratory animal training courses for a research project.**

Since you as a participant can evaluate best, how good the training on the simulator and the preparation for the practical exercises on the live animal is, we would kindly ask you for **your personal feedback on the strengths and weaknesses of the simulators** on which you have trained in the course.

Thank you very much for your participation!

The SimulRATor-Team

Further information can be found at www.simulrator.de or write to kontakt@simulrator.de.

Part 1 of 2 Rat Simulator C

ID: ___________________

← Please remember your ID for the 2nd part. Thank you!

Privacy Notice: Your participation in the survey is voluntary. The survey data is anonymously stored and managed. Under no circumstances will data be passed on to third parties. We collect no personal information from you. No conclusions on your participation are possible. Only the people involved in the research project have access to the information provided by you within the questionnaire and are obliged to maintain secrecy. The information provided is used for research purposes only. You can cancel the survey at any time without giving reasons.

1. How well were you able to apply the procedural techniques on the Rat Simulator C?

Techniques	Extremely good 1	Quite good 2	Slightly good 3	Slightly bad 4	Quite bad 5	Extremely bad 6	Technique was not trained.
Handling - transfer from cage to cage	o	o	o	o	o	o	o
Restraint - scruffing	o	o	o	o	o	o	o
Restraint - over the shoulder grip	o	o	o	o	o	o	o
Restraint - middle shoulder grip	o	o	o	o	o	o	o
Restraint - under the shoulder grip	o	o	o	o	o	o	o
Intravenous administration via lateral tail vein	o	o	o	o	o	o	o
Blood sampling from saphenous vein	o	o	o	o	o	o	o
Blood sampling from lateral tail vein	o	o	o	o	o	o	o
Blood sampling from heart	o	o	o	o	o	o	o
Other: ______________________________	o	o	o	o	o	o	o
______________________________	o	o	o	o	o	o	o

2. How well do the following statements about the training with the Rat Simulator C apply?

	Completely applies 1	Largely applies 2	Slightly applies 3	Slightly does not apply 4	Largely does not apply 5	Does not apply at all 6
The number of participants per Rat Simulator C was appropriate.	o	o	o	o	o	o
The training duration on the Rat Simulator C was sufficient.	o	o	o	o	o	o
The Rat Simulator C provides satisfactory training for the techniques.	o	o	o	o	o	o
By training with the Rat Simulator C, I feel better prepared for course training on the live rat.	o	o	o	o	o	o

3. If there were material related difficulties using the Rat Simulator C, please give us a brief description of these.

Technique: ____

Description of the complication: ____

____

____

Technique: ____

Description of the complication: ____

____

____

Do you have any recommendations, praise or criticism? We highly appreciate your feedback!

Thank you very much for your participation!

The SimulRATor-Team kontakt@simulrator.de

Welcome to „SimulRATor“!

We invite you to take part in our survey on simulators in laboratory animal science.

We are a team of scientists of the Institutes of Veterinary Anatomy, of Animal Welfare, Animal Behavior and Laboratory Animal Science and of Veterinary Epidemiology and Biometry at the Department of Veterinary Medicine of the Freie Universität Berlin and **are evaluating all currently commercially available rat and mouse simulators in laboratory animal training courses for a research project.**

Since you as a participant can evaluate best, how good the training on the simulator and the preparation for the practical exercises on the live animal is, we would kindly ask you for **your personal feedback on the strengths and weaknesses of the simulators** on which you have trained in the course.

Thank you very much for your participation!

The SimulRATor-Team

Further information can be found at www.simulrator.de or write to kontakt@simulrator.de.

Part 2 of 2 Rat Simulator C

ID: ___________________

Privacy Notice: Your participation in the survey is voluntary. The survey data is anonymously stored and managed. Under no circumstances will data be passed on to third parties. We collect no personal information from you. No conclusions on your participation are possible. Only the people involved in the research project have access to the information provided by you within the questionnaire and are obliged to maintain secrecy. The information provided is used for research purposes only. You can cancel the survey at any time without giving reasons.

1. How well were you able to manage handling, restraint, and ear punching on the live rat?

Techniques	Extremely good 1	Quite good 2	Slightly good 3	Slightly bad 4	Quite bad 5	Extremely bad 6	Technique was not trained.
Handling - transfer from cage to cage	o	o	o	o	o	o	o
Restraint - scruffing	o	o	o	o	o	o	o
Restraint - over the shoulder grip	o	o	o	o	o	o	o
Restraint - middle shoulder grip	o	o	o	o	o	o	o
Restraint – under the shoulder grip	o	o	o	o	o	o	o
Ear punching	o	o	o	o	o	o	o
Other: ________________________________	o	o	o	o	o	o	o
________________________________	o	o	o	o	o	o	o

2. How well were you able to manage the following procedural techniques on the live rat?

Please also tick the box in the last column if the rat was anesthetized for the method.

Techniques	Extremely good 1	Quite good 2	Slightly good 3	Slightly bad 4	Quite bad 5	Extremely bad 6	Not trained.	Rat in anesthesia.
Feeding the rat	o	o	o	o	o	o	o	☐
Administration by oral gavage	o	o	o	o	o	o	o	☐
Subcutaneous administration under skin over the neck	o	o	o	o	o	o	o	☐
Subcutaneous administration under skin over the flank	o	o	o	o	o	o	o	☐
Intramuscular administration	o	o	o	o	o	o	o	☐
Intraperitoneal administration	o	o	o	o	o	o	o	☐
Intravenouse administration via dorsal penis vein	o	o	o	o	o	o	o	☐
Intravenous administration via lateral tail vein	o	o	o	o	o	o	o	☐
Blood sampling from sublingual vein	o	o	o	o	o	o	o	☐
Blood sampling from retro-orbital plexus	o	o	o	o	o	o	o	☐
Blood sampling from saphenous vein	o	o	o	o	o	o	o	☐
Blood sampling from lateral tail vein	o	o	o	o	o	o	o	☐
Blood sampling from heart	o	o	o	o	o	o	o	☐
Other: ________________________________	o	o	o	o	o	o	o	☐
________________________________	o	o	o	o	o	o	o	☐

3. Which 3 techniques on the live rat are in your opinion particularly demanding for the performer?

Please name 3 techniques from 1st and 2nd question. Please specify the techniques completely.

1. ____

2. ____

3. ____

4. Please select from the list below the 5 techniques on the live rat, for which you consider a preparatory training on simulators to be particularly useful.

Max. 5 techniques can be chosen.

- ☐ Handling - transfer from cage to cage
- ☐ Restraint (different techniques)
- ☐ Ear punching
- ☐ Feeding the rat
- ☐ Administration by oral gavage
- ☐ Subcutaneous administration under skin over the neck
- ☐ Subcutaneous administration under skin over the flank
- ☐ Intramuscular administration
- ☐ Intraperitoneal administration
- ☐ Intravenous administration via dorsal penis vein
- ☐ Intravenous administration via lateral tail vein
- ☐ Blood sampling from sublingual vein
- ☐ Blood sampling from retro-orbital plexus
- ☐ Blood sampling from saphenous vein
- ☐ Blood sampling from lateral tail vein
- ☐ Blood sampling from heart

5. Would you also like to have a simulator training for other methods on the rat?

If so, please describe which technique(s) is/are important to you.

Your personal feedback about the Rat Simulator C

6. How realistic are the following features of the Rat Simulator C in comparison to a live rat?

Features	Extremely realistic 1	Quite realistic 2	Slightly realistic 3	Slightly unrealistic 4	Quite unrealistic 5	Extremely unrealistic 6
General appearance	o	o	o	o	o	o
Haptics	o	o	o	o	o	o
Movability of skin over neck	o	o	o	o	o	o
Movability of skin over flank	o	o	o	o	o	o
Consistency of skin surface	o	o	o	o	o	o
Body height	o	o	o	o	o	o
Body weight	o	o	o	o	o	o
Body shape	o	o	o	o	o	o
Proportions	o	o	o	o	o	o

7. How realistic was the training with the Rat Simulator C in comparison to a live rat for you?

Techniques	Extremely realistic 1	Quite realistic 2	Slightly realistic 3	Slightly unrealistic 4	Quite unrealistic 5	Extremely unrealistic 6	Technique was not trained.
Handling - transfer from cage to cage	o	o	o	o	o	o	o
Restraint - scruffing	o	o	o	o	o	o	o
Restraint - over the shoulder grip	o	o	o	o	o	o	o
Restraint - middle shoulder grip	o	o	o	o	o	o	o
Restraint - under the shoulder grip	o	o	o	o	o	o	o
Intravenous administration via lateral tail vein	o	o	o	o	o	o	o
Blood sampling from saphenous vein	o	o	o	o	o	o	o
Blood sampling from lateral tail vein	o	o	o	o	o	o	o
Blood sampling from heart	o	o	o	o	o	o	o
Other: ______________________________	o	o	o	o	o	o	o
______________________________	o	o	o	o	o	o	o

8. How well do the following statements about the training with the Rat Simulator C apply?

	Completely applies 1	Largely applies 2	Slightly applies 3	Slightly does not apply 4	Largely does not apply 5	Does not apply at all 6
I felt more secure in performing **handling und restraint** on the live rat.	o	o	o	o	o	o
I felt more secure in performing **procedural techniques** on the live rat.	o	o	o	o	o	o

9. How well did the Rat Simulator C prepare you for the entire course training on the live rat?

Extremely good	Quite good	Slightly good	Slightly bad	Quite bad	Extremely bad
1	2	3	4	5	6
o	o	o	o	o	o

10. How well did the Rat Simulator C prepare you for the following techniques in the course training on the live rat?

Techniques	Extremely good 1	Quite good 2	Slightly good 3	Slightly bad 4	Quite bad 5	Extremely bad 6	Technique was not trained.
Handling - transfer from cage to cage	o	o	o	o	o	o	o
Restraint - scruffing	o	o	o	o	o	o	o
Restraint - over the shoulder grip	o	o	o	o	o	o	o
Restraint - middle shoulder grip	o	o	o	o	o	o	o
Restraint - under the shoulder grip	o	o	o	o	o	o	o
Intravenous administration via lateral tail vein	o	o	o	o	o	o	o
Blood sampling from saphenous vein	o	o	o	o	o	o	o
Blood sampling from lateral tail vein	o	o	o	o	o	o	o
Blood sampling from heart	o	o	o	o	o	o	o
Other: ________________________________	o	o	o	o	o	o	o
________________________________	o	o	o	o	o	o	o

11. What did you particularly like about the Rat Simulator C?

12. What did you not like about the Rat Simulator C?

13. What would you like to improve about the Rat Simulator C?

14. Which species are you expecting to work with for scientific reasons in the next 6 months after you have passed the course?
Multiple answers for species are possible.

- ☐ Rat
- ☐ Mouse
- ☐ Rabbit
- ☐ Guinea pig
- ☐ Hamster
- ☐ Pig
- ☐ Sheep
- ☐ Goat
- ☐ Cow
- ☐ Horse
- ☐ Dog
- ☐ Cat
- ☐ Other_____________________________
- ☐ None
- ☐ I cannot judge.

15. How old are you?

________ years

16. What is your gender?

- ☐ Male
- ☐ Female
- ☐ Other
- ☐ No answer

17. Please mark your <u>highest</u> educational degree you have achieved.
Only one answer is possible.

- ☐ (Still) no qualification (yet)
- ☐ Apprenticeship/Vocational training
- ☐ Technical college qualification
- ☐ University of Applied Sciences Degree
- ☐ Bachelor's Degree
- ☐ Master's Degree, diploma or state examination
- ☐ Ph.D. /Doctoral degree
- ☐ Other: ____

18. Please complete the sentence below.
Only one answer is possible.

I am participating in the course as …

- ☐ …an academic employee.
- ☐ …a technical assistant.
- ☐ …an apprentice.
- ☐ …a student.
- ☐ …other: ____.

19. What is your discipline?
Only one answer is possible.

- ☐ Human Medicine
- ☐ Veterinary Medicine
- ☐ Dental Medicine
- ☐ Pharmacology
- ☐ Biology
- ☐ Chemistry
- ☐ Physics
- ☐ Biotechnology
- ☐ Nutritional Science
- ☐ Other: ____

20. Had you already had experience …
Only one answer is possible.

…in handling <u>rats</u> before the course?

- ☐ Yes, a lot of.
- ☐ Yes, a little bit.
- ☐ No, not at all.

21. Had you already had experience …
Only one answer is possible.

…in handling <u>mice</u> before the course?

- ☐ Yes, a lot of.
- ☐ Yes, a little bit.
- ☐ No, not at all.

22. Had you already worked with other simulator(s) before the course?
Multiple answers for simulators are possible. Please describe the model in brief.

- ☐ Yes, …
  - ☐ …with rat simulators: Model: _______________________________________
  - ☐ …with mouse simulators: Model: ______________________________ _________
  - ☐ …with other simulators: Model: _______________________________________
- ☐ No.

Do you have any recommendations, praise or criticism? We highly appreciate your feedback!

Thank you very much for your participation!

The SimulRATor-Team

web: www.simulator.de mail: kontakt@simulrator.de

Welcome to „SimulRATor“!

We invite you to take part in our survey on simulators in laboratory animal science.

We are a team of scientists of the Institutes of Veterinary Anatomy, of Animal Welfare, Animal Behavior and Laboratory Animal Science and of Veterinary Epidemiology and Biometry at the Department of Veterinary Medicine of the Freie Universität Berlin and **are evaluating all currently commercially available rat and mouse simulators in laboratory animal training courses for a research project.**

Since you as a participant can evaluate best, how good the training on the simulator and the preparation for the practical exercises on the live animal is, we would kindly ask you for **your personal feedback on the strengths and weaknesses of the simulators** on which you have trained in the course.

Thank you very much for your participation!

The SimulRATor-Team

Further information can be found at www.simulrator.de or write to kontakt@simulrator.de.

Part 1 of 2 Rat Simulator D

ID: ____________________

← **Please remember your ID for the 2nd part. Thank you!**

Privacy Notice: Your participation in the survey is voluntary. The survey data is anonymously stored and managed. Under no circumstances will data be passed on to third parties. We collect no personal information from you. No conclusions on your participation are possible. Only the people involved in the research project have access to the information provided by you within the questionnaire and are obliged to maintain secrecy. The information provided is used for research purposes only. You can cancel the survey at any time without giving reasons.

1. How well were you able to apply the procedural techniques on the Rat Simulator D?

Techniques	Extremely good 1	Quite good 2	Slightly good 3	Slightly bad 4	Quite bad 5	Extremely bad 6	Technique was not trained.
Handling - transfer from cage to cage	o	o	o	o	o	o	o
Restraint - scruffing	o	o	o	o	o	o	o
Restraint - over the shoulder grip	o	o	o	o	o	o	o
Restraint - middle shoulder grip	o	o	o	o	o	o	o
Restraint - under the shoulder grip	o	o	o	o	o	o	o
Ear punching	o	o	o	o	o	o	o
Intravenous administration via lateral tail vein	o	o	o	o	o	o	o
Blood sampling from lateral tail vein	o	o	o	o	o	o	o
Other: ______________________________	o	o	o	o	o	o	o
______________________________	o	o	o	o	o	o	o

2. How well do the following statements about the training with the Rat Simulator D apply?

	Completely applies 1	Largely applies 2	Slightly applies 3	Slightly does not apply 4	Largely does not apply 5	Does not apply at all 6
The number of participants per Rat Simulator D was appropriate.	o	o	o	o	o	o
The training duration on the Rat Simulator D was sufficient.	o	o	o	o	o	o
The Rat Simulator D provides satisfactory training for the techniques.	o	o	o	o	o	o
By training with the Rat Simulator D, I feel better prepared for course training on the live rat.	o	o	o	o	o	o

3. If there were material related difficulties using the Rat Simulator D, please give us a brief description of these.

Technique: ______________________________

Description of the complication: ______________________________

____

____

Technique: ______________________________

Description of the complication: ______________________________

____

____

Do you have any recommendations, praise or criticism? We highly appreciate your feedback!

Thank you very much for your participation!

The SimulRATor-Team kontakt@simulrator.de

Welcome to „SimulRATor"!

We invite you to take part in our survey on simulators in laboratory animal science.

We are a team of scientists of the Institutes of Veterinary Anatomy, of Animal Welfare, Animal Behavior and Laboratory Animal Science and of Veterinary Epidemiology and Biometry at the Department of Veterinary Medicine of the Freie Universität Berlin and **are evaluating all currently commercially available rat and mouse simulators in laboratory animal training courses for a research project.**

Since you as a participant can evaluate best, how good the training on the simulator and the preparation for the practical exercises on the live animal is, we would kindly ask you for **your personal feedback on the strengths and weaknesses of the simulators** on which you have trained in the course.

Thank you very much for your participation!

The SimulRATor-Team

Further information can be found at www.simulrator.de or write to kontakt@simulrator.de.

Part 2 of 2 Rat Simulator D

ID: ___________________

Privacy Notice: Your participation in the survey is voluntary. The survey data is anonymously stored and managed. Under no circumstances will data be passed on to third parties. We collect no personal information from you. No conclusions on your participation are possible. Only the people involved in the research project have access to the information provided by you within the questionnaire and are obliged to maintain secrecy. The information provided is used for research purposes only. You can cancel the survey at any time without giving reasons.

1. How well were you able to manage handling, restraint, and ear punching on the live rat?

Techniques	Extremely good 1	Quite good 2	Slightly good 3	Slightly bad 4	Quite bad 5	Extremely bad 6	Technique was not trained.
Handling - transfer from cage to cage	o	o	o	o	o	o	o
Restraint - scruffing	o	o	o	o	o	o	o
Restraint - over the shoulder grip	o	o	o	o	o	o	o
Restraint - middle shoulder grip	o	o	o	o	o	o	o
Restraint – under the shoulder grip	o	o	o	o	o	o	o
Ear punching	o	o	o	o	o	o	o
Other: ________________________________	o	o	o	o	o	o	o
________________________________	o	o	o	o	o	o	o

2. How well were you able to manage the following procedural techniques on the live rat?

Please also tick the box in the last column if the rat was anesthetized for the method.

Techniques	Extremely good 1	Quite good 2	Slightly good 3	Slightly bad 4	Quite bad 5	Extremely bad 6	Not trained.	Rat in anesthesia.
Feeding the rat	o	o	o	o	o	o	o	☐
Administration by oral gavage	o	o	o	o	o	o	o	☐
Subcutaneous administration under skin over the neck	o	o	o	o	o	o	o	☐
Subcutaneous administration under skin over the flank	o	o	o	o	o	o	o	☐
Intramuscular administration	o	o	o	o	o	o	o	☐
Intraperitoneal administration	o	o	o	o	o	o	o	☐
Intravenouse administration via dorsal penis vein	o	o	o	o	o	o	o	☐
Intravenous administration via lateral tail vein	o	o	o	o	o	o	o	☐
Blood sampling from sublingual vein	o	o	o	o	o	o	o	☐
Blood sampling from retro-orbital plexus	o	o	o	o	o	o	o	☐
Blood sampling from saphenous vein	o	o	o	o	o	o	o	☐
Blood sampling from lateral tail vein	o	o	o	o	o	o	o	☐
Blood sampling from heart	o	o	o	o	o	o	o	☐
Other: ________________________________	o	o	o	o	o	o	o	☐
________________________________	o	o	o	o	o	o	o	☐

3. Which 3 techniques on the live rat are in your opinion particularly demanding for the performer?

Please name 3 techniques from 1st and 2nd question. Please specify the techniques completely.

1. ____

2. ____

3. ____

4. Please select from the list below the 5 techniques on the live rat, for which you consider a preparatory training on simulators to be particularly useful.

Max. 5 techniques can be chosen.

- ☐ Handling - transfer from cage to cage
- ☐ Restraint (different techniques)
- ☐ Ear punching
- ☐ Feeding the rat
- ☐ Administration by oral gavage
- ☐ Subcutaneous administration under skin over the neck
- ☐ Subcutaneous administration under skin over the flank
- ☐ Intramuscular administration
- ☐ Intraperitoneal administration
- ☐ Intravenous administration via dorsal penis vein
- ☐ Intravenous administration via lateral tail vein
- ☐ Blood sampling from sublingual vein
- ☐ Blood sampling from retro-orbital plexus
- ☐ Blood sampling from saphenous vein
- ☐ Blood sampling from lateral tail vein
- ☐ Blood sampling from heart

5. Would you also like to have a simulator training for other methods on the rat?

If so, please describe which technique(s) is/are important to you.

Your personal feedback about the Rat Simulator D

6. How realistic are the following features of the Rat Simulator D in comparison to a live rat?

Features	Extremely realistic 1	Quite realistic 2	Slightly realistic 3	Slightly unrealistic 4	Quite unrealistic 5	Extremely unrealistic 6
General appearance	o	o	o	o	o	o
Haptics	o	o	o	o	o	o
Movability of skin over neck	o	o	o	o	o	o
Movability of skin over flank	o	o	o	o	o	o
Consistency of skin surface	o	o	o	o	o	o
Body height	o	o	o	o	o	o
Body weight	o	o	o	o	o	o
Body shape	o	o	o	o	o	o
Proportions	o	o	o	o	o	o

7. How realistic was the training with the Rat Simulator D in comparison to a live rat for you?

Techniques	Extremely realistic 1	Quite realistic 2	Slightly realistic 3	Slightly unrealistic 4	Quite unrealistic 5	Extremely unrealistic 6	Technique was not trained.
Handling - transfer from cage to cage	o	o	o	o	o	o	o
Restraint - scruffing	o	o	o	o	o	o	o
Restraint - over the shoulder grip	o	o	o	o	o	o	o
Restraint - middle shoulder grip	o	o	o	o	o	o	o
Restraint - under the shoulder grip	o	o	o	o	o	o	o
Ear punching	o	o	o	o	o	o	o
Intravenous administration via lateral tail vein	o	o	o	o	o	o	o
Blood sampling from lateral tail vein	o	o	o	o	o	o	o
Other: ______________________________	o	o	o	o	o	o	o
______________________________	o	o	o	o	o	o	o

8. How well do the following statements about the training with the Rat Simulator D apply?

	Completely applies 1	Largely applies 2	Slightly applies 3	Slightly does not apply 4	Largely does not apply 5	Does not apply at all 6
I felt more secure in performing **handling und restraint** on the live rat.	o	o	o	o	o	o
I felt more secure in performing **procedural techniques** on the live rat.	o	o	o	o	o	o

9. How well did the Rat Simulator D prepare you for the entire course training on the live rat?

Extremely good	Quite good	Slightly good	Slightly bad	Quite bad	Extremely bad
1	2	3	4	5	6
○	○	○	○	○	○

10. How well did the Rat Simulator D prepare you for the following techniques in the course training on the live rat?

Techniques	Extremely good 1	Quite good 2	Slightly good 3	Slightly bad 4	Quite bad 5	Extremely bad 6	Technique was not trained.
Handling - transfer from cage to cage	○	○	○	○	○	○	○
Restraint - scruffing	○	○	○	○	○	○	○
Restraint - over the shoulder grip	○	○	○	○	○	○	○
Restraint - middle shoulder grip	○	○	○	○	○	○	○
Restraint - under the shoulder grip	○	○	○	○	○	○	○
Ear punching	○	○	○	○	○	○	○
Intravenous administration via lateral tail vein	○	○	○	○	○	○	○
Blood sampling from lateral tail vein	○	○	○	○	○	○	○
Other: ________________________________	○	○	○	○	○	○	○
________________________________	○	○	○	○	○	○	○

11. What did you particularly like about the Rat Simulator D?

12. What did you not like about the Rat Simulator D?

13. What would you like to improve about the Rat Simulator D?

14. Which species are you expecting to work with for scientific reasons in the next 6 months after you have passed the course?
Multiple answers for species are possible.

- ◻ Rat
- ◻ Mouse
- ◻ Rabbit
- ◻ Guinea pig
- ◻ Hamster
- ◻ Pig
- ◻ Sheep
- ◻ Goat
- ◻ Cow
- ◻ Horse
- ◻ Dog
- ◻ Cat
- ◻ Other____________________________
- ◻ None
- ◻ I cannot judge.

15. How old are you?

________ years

16. What is your gender?

- □ Male
- □ Female
- □ Other
- □ No answer

17. Please mark your <u>highest</u> educational degree you have achieved.
Only one answer is possible.

- □ (Still) no qualification (yet)
- □ Apprenticeship/Vocational training
- □ Technical college qualification
- □ University of Applied Sciences Degree
- □ Bachelor's Degree
- □ Master's Degree, diploma or state examination
- □ Ph.D. /Doctoral degree
- □ Other: ____

18. Please complete the sentence below.
Only one answer is possible.

I am participating in the course as …

- □ …an academic employee.
- □ …a technical assistant.
- □ …an apprentice.
- □ …a student.
- □ …other: ____.

19. What is your discipline?
Only one answer is possible.

- □ Human Medicine
- □ Veterinary Medicine
- □ Dental Medicine
- □ Pharmacology
- □ Biology
- □ Chemistry
- □ Physics
- □ Biotechnology
- □ Nutritional Science
- □ Other: ____

20. Had you already had experience …
Only one answer is possible.

…in handling <u>rats</u> before the course?

- □ Yes, a lot of.
- □ Yes, a little bit.
- □ No, not at all.

21. Had you already had experience …
Only one answer is possible.

…in handling <u>mice</u> before the course?

- □ Yes, a lot of.
- □ Yes, a little bit.
- □ No, not at all.

22. Had you already worked with other simulator(s) before the course?
Multiple answers for simulators are possible. Please describe the model in brief.

- □ Yes, …
  - □ …with rat simulators: Model: ____
  - □ …with mouse simulators: Model: ______________________________ __________
  - □ …with other simulators: Model: ____
- □ No.

Do you have any recommendations, praise or criticism? We highly appreciate your feedback!

Thank you very much for your participation!

The SimulRATor-Team

web: www.simulator.de mail: kontakt@simulrator.de

DEUTSCHES ZENTRUM
ZUM SCHUTZ VON
VERSUCHSTIEREN

Freie Universität Berlin

Welcome to „SimulRATor“!

We invite you to take part in our survey on simulators in laboratory animal science.

We are a team of scientists of the Institutes of Veterinary Anatomy, of Animal Welfare, Animal Behavior and Laboratory Animal Science and of Veterinary Epidemiology and Biometry at the Department of Veterinary Medicine of the Freie Universität Berlin and **are evaluating all currently commercially available rat and mouse simulators in laboratory animal training courses for a research project.**

Since you as a participant can evaluate best, how good the training on the simulator and the preparation for the practical exercises on the live animal is, we would kindly ask you for **your personal feedback on the strengths and weaknesses of the simulators** on which you have trained in the course.

Thank you very much for your participation!

The SimulRATor-Team

Further information can be found at www.simulrator.de or write to kontakt@simulrator.de.

Part 1 of 2 Rat Simulator E

ID: ___________________

← Please remember your ID for the 2nd part. Thank you!

Privacy Notice: Your participation in the survey is voluntary. The survey data is anonymously stored and managed. Under no circumstances will data be passed on to third parties. We collect no personal information from you. No conclusions on your participation are possible. Only the people involved in the research project have access to the information provided by you within the questionnaire and are obliged to maintain secrecy. The information provided is used for research purposes only. You can cancel the survey at any time without giving reasons.

1. How well were you able to apply the procedural techniques on the Rat Simulator E?

Techniques	Extremely good 1	Quite good 2	Slightly good 3	Slightly bad 4	Quite bad 5	Extremely bad 6	Technique was not trained.
Handling - transfer from cage to cage	o	o	o	o	o	o	o
Restraint - scruffing	o	o	o	o	o	o	o
Restraint - over the shoulder grip	o	o	o	o	o	o	o
Restraint - middle shoulder grip	o	o	o	o	o	o	o
Restraint - under the shoulder grip	o	o	o	o	o	o	o
Administration by oral gavage	o	o	o	o	o	o	o
Subcutaneous administration under skin over the neck	o	o	o	o	o	o	o
Subcutaneous administration under skin over the flank	o	o	o	o	o	o	o
Intramuscular administration	o	o	o	o	o	o	o
Intravenous administration via lateral tail vein	o	o	o	o	o	o	o
Blood sampling from lateral tail vein	o	o	o	o	o	o	o
Other: ______________________	o	o	o	o	o	o	o
______________________	o	o	o	o	o	o	o

2. How well do the following statements about the training with the Rat Simulator E apply?

	Completely applies 1	Largely applies 2	Slightly applies 3	Slightly does not apply 4	Largely does not apply 5	Does not apply at all 6
The number of participants per Rat Simulator E was appropriate.	o	o	o	o	o	o
The training duration on the Rat Simulator E was sufficient.	o	o	o	o	o	o
The Rat Simulator E provides satisfactory training for the techniques.	o	o	o	o	o	o
By training with the Rat Simulator E, I feel better prepared for course training on the live rat.	o	o	o	o	o	o

3. If there were material related difficulties using the Rat Simulator E, please give us a brief description of these.

Technique: ____

Description of the complication: ____

____

____

Technique: ____

Description of the complication: ____

____

____

Do you have any recommendations, praise or criticism? We highly appreciate your feedback!

Thank you very much for your participation!

The SimulRATor-Team kontakt@simulrator.de

Welcome to „SimulRATor“!

We invite you to take part in our survey on simulators in laboratory animal science.

We are a team of scientists of the Institutes of Veterinary Anatomy, of Animal Welfare, Animal Behavior and Laboratory Animal Science and of Veterinary Epidemiology and Biometry at the Department of Veterinary Medicine of the Freie Universität Berlin and **are evaluating all currently commercially available rat and mouse simulators in laboratory animal training courses for a research project.**

Since you as a participant can evaluate best, how good the training on the simulator and the preparation for the practical exercises on the live animal is, we would kindly ask you for **your personal feedback on the strengths and weaknesses of the simulators** on which you have trained in the course.

Thank you very much for your participation!

The SimulRATor-Team

Further information can be found at www.simulrator.de or write to kontakt@simulrator.de.

Part 2 of 2 Rat Simulator E

ID: ___________________

Privacy Notice: Your participation in the survey is voluntary. The survey data is anonymously stored and managed. Under no circumstances will data be passed on to third parties. We collect no personal information from you. No conclusions on your participation are possible. Only the people involved in the research project have access to the information provided by you within the questionnaire and are obliged to maintain secrecy. The information provided is used for research purposes only. You can cancel the survey at any time without giving reasons.

1. How well were you able to manage handling, restraint, and ear punching on the live rat?

Techniques	Extremely good 1	Quite good 2	Slightly good 3	Slightly bad 4	Quite bad 5	Extremely bad 6	Technique was not trained.
Handling - transfer from cage to cage	o	o	o	o	o	o	o
Restraint - scruffing	o	o	o	o	o	o	o
Restraint - over the shoulder grip	o	o	o	o	o	o	o
Restraint - middle shoulder grip	o	o	o	o	o	o	o
Restraint – under the shoulder grip	o	o	o	o	o	o	o
Ear punching	o	o	o	o	o	o	o
Other: ________________________________	o	o	o	o	o	o	o
________________________________	o	o	o	o	o	o	o

2. How well were you able to manage the following procedural techniques on the live rat?
Please also tick the box in the last column if the rat was anesthetized for the method.

Techniques	Extremely good 1	Quite good 2	Slightly good 3	Slightly bad 4	Quite bad 5	Extremely bad 6	Not trained.	Rat in anesthesia.
Feeding the rat	o	o	o	o	o	o	o	☐
Administration by oral gavage	o	o	o	o	o	o	o	☐
Subcutaneous administration under skin over the neck	o	o	o	o	o	o	o	☐
Subcutaneous administration under skin over the flank	o	o	o	o	o	o	o	☐
Intramuscular administration	o	o	o	o	o	o	o	☐
Intraperitoneal administration	o	o	o	o	o	o	o	☐
Intravenouse administration via dorsal penis vein	o	o	o	o	o	o	o	☐
Intravenous administration via lateral tail vein	o	o	o	o	o	o	o	☐
Blood sampling from sublingual vein	o	o	o	o	o	o	o	☐
Blood sampling from retro-orbital plexus	o	o	o	o	o	o	o	☐
Blood sampling from saphenous vein	o	o	o	o	o	o	o	☐
Blood sampling from lateral tail vein	o	o	o	o	o	o	o	☐
Blood sampling from heart	o	o	o	o	o	o	o	☐
Other: ________________________________	o	o	o	o	o	o	o	☐
________________________________	o	o	o	o	o	o	o	☐

3. Which 3 techniques on the live rat are in your opinion particularly demanding for the performer?

Please name 3 techniques from 1st and 2nd question. Please specify the techniques completely.

1. ____

2. ____

3. ____

4. Please select from the list below the 5 techniques on the live rat, for which you consider a preparatory training on simulators to be particularly useful.

Max. 5 techniques can be chosen.

☐ Handling - transfer from cage to cage
☐ Restraint (different techniques)
☐ Ear punching
☐ Feeding the rat
☐ Administration by oral gavage
☐ Subcutaneous administration under skin over the neck
☐ Subcutaneous administration under skin over the flank
☐ Intramuscular administration
☐ Intraperitoneal administration
☐ Intravenous administration via dorsal penis vein
☐ Intravenous administration via lateral tail vein
☐ Blood sampling from sublingual vein
☐ Blood sampling from retro-orbital plexus
☐ Blood sampling from saphenous vein
☐ Blood sampling from lateral tail vein
☐ Blood sampling from heart

5. Would you also like to have a simulator training for other methods on the rat?
If so, please describe which technique(s) is/are important to you.

Your personal feedback about the Rat Simulator E

6. How realistic are the following features of the Rat Simulator E in comparison to a live rat?

**Features**	**Extremely realistic 1**	**Quite realistic 2**	**Slightly realistic 3**	**Slightly unrealistic 4**	**Quite unrealistic 5**	**Extremely unrealistic 6**
General appearance	o	o	o	o	o	o
Haptics	o	o	o	o	o	o
Movability of skin over neck	o	o	o	o	o	o
Movability of skin over flank	o	o	o	o	o	o
Consistency of skin surface	o	o	o	o	o	o
Body height	o	o	o	o	o	o
Body weight	o	o	o	o	o	o
Body shape	o	o	o	o	o	o
Proportions	o	o	o	o	o	o

7. How realistic was the training with the Rat Simulator E in comparison to a live rat for you?

**Techniques**	**Extremely realistic 1**	**Quite realistic 2**	**Slightly realistic 3**	**Slightly unrealistic 4**	**Quite unrealistic 5**	**Extremely unrealistic 6**	**Technique was not trained.**
Handling - transfer from cage to cage	o	o	o	o	o	o	o
Restraint - scruffing	o	o	o	o	o	o	o
Restraint - over the shoulder grip	o	o	o	o	o	o	o
Restraint - middle shoulder grip	o	o	o	o	o	o	o
Restraint - under the shoulder grip	o	o	o	o	o	o	o
Administration by oral gavage	o	o	o	o	o	o	o
Subcutaneous administration under skin over the neck	o	o	o	o	o	o	o
Subcutaneous administration under skin over the flank	o	o	o	o	o	o	o
Intramuscular administration	o	o	o	o	o	o	o
Intravenous administration via lateral tail vein	o	o	o	o	o	o	o
Blood sampling from lateral tail vein	o	o	o	o	o	o	o
Other: ___________________________	o	o	o	o	o	o	o
___________________________	o	o	o	o	o	o	o

8. How well do the following statements about the training with the Rat Simulator E apply?

	**Completely applies 1**	**Largely applies 2**	**Slightly applies 3**	**Slightly does not apply 4**	**Largely does not apply 5**	**Does not apply at all 6**
I felt more secure in performing **handling und restraint** on the live rat.	o	o	o	o	o	o
I felt more secure in performing **procedural techniques** on the live rat.	o	o	o	o	o	o

9. How well did the Rat Simulator E prepare you for the entire course training on the live rat?

Extremely good	Quite good	Slightly good	Slightly bad	Quite bad	Extremely bad
1	2	3	4	5	6
○	○	○	○	○	○

10. How well did the Rat Simulator E prepare you for the following techniques in the course training on the live rat?

Techniques	Extremely good 1	Quite good 2	Slightly good 3	Slightly bad 4	Quite bad 5	Extremely bad 6	Technique was not trained.
Handling - transfer from cage to cage	○	○	○	○	○	○	○
Restraint - scruffing	○	○	○	○	○	○	○
Restraint - over the shoulder grip	○	○	○	○	○	○	○
Restraint - middle shoulder grip	○	○	○	○	○	○	○
Restraint - under the shoulder grip	○	○	○	○	○	○	○
Administration by oral gavage	○	○	○	○	○	○	○
Subcutaneous administration under skin over the neck	○	○	○	○	○	○	○
Subcutaneous administration under skin over the flank	○	○	○	○	○	○	○
Intramuscular administration	○	○	○	○	○	○	○
Intravenous administration via lateral tail vein	○	○	○	○	○	○	○
Blood sampling from lateral tail vein	○	○	○	○	○	○	○
Other: ___________________________________	○	○	○	○	○	○	○
___________________________________	○	○	○	○	○	○	○

11. What did you particularly like about the Rat Simulator E?

12. What did you not like about the Rat Simulator E?

13. What would you like to improve about the Rat Simulator E?

14. Which species are you expecting to work with for scientific reasons in the next 6 months after you have passed the course?
Multiple answers for species are possible.

- □ Rat
- □ Mouse
- □ Rabbit
- □ Guinea pig
- □ Hamster
- □ Pig
- □ Sheep
- □ Goat
- □ Cow
- □ Horse
- □ Dog
- □ Cat
- □ Other______________________________
- □ None
- □ I cannot judge.

15. How old are you?

________ years

16. What is your gender?

- ☐ Male
- ☐ Female
- ☐ Other
- ☐ No answer

17. Please mark your <u>highest</u> educational degree you have achieved.
Only one answer is possible.

- ☐ (Still) no qualification (yet)
- ☐ Apprenticeship/Vocational training
- ☐ Technical college qualification
- ☐ University of Applied Sciences Degree
- ☐ Bachelor's Degree
- ☐ Master's Degree, diploma or state examination
- ☐ Ph.D. /Doctoral degree
- ☐ Other: ____

18. Please complete the sentence below.
Only one answer is possible.

I am participating in the course as …

- ☐ …an academic employee.
- ☐ …a technical assistant.
- ☐ …an apprentice.
- ☐ …a student.
- ☐ …other: ____.

19. What is your discipline?
Only one answer is possible.

- ☐ Human Medicine
- ☐ Veterinary Medicine
- ☐ Dental Medicine
- ☐ Pharmacology
- ☐ Biology
- ☐ Chemistry
- ☐ Physics
- ☐ Biotechnology
- ☐ Nutritional Science
- ☐ Other: ____

Please turn over.

20. Had you already had experience …
Only one answer is possible.

…in handling <u>rats</u> before the course?

- ☐ Yes, a lot of.
- ☐ Yes, a little bit.
- ☐ No, not at all.

21. Had you already had experience …
Only one answer is possible.

…in handling <u>mice</u> before the course?

- ☐ Yes, a lot of.
- ☐ Yes, a little bit.
- ☐ No, not at all.

22. Had you already worked with other simulator(s) before the course?
Multiple answers for simulators are possible. Please describe the model in brief.

- ☐ Yes, …
  - ☐ …with rat simulators: Model: _______________________________________
  - ☐ …with mouse simulators: Model: ______________________________ _________
  - ☐ …with other simulators: Model: ____
- ☐ No.

Do you have any recommendations, praise or criticism? We highly appreciate your feedback!

Thank you very much for your participation!

The SimulRATor-Team

web: www.simulator.de mail: kontakt@simulrator.de

Herzlich willkommen bei „SimulRATor“

Wir laden Sie ein, an unserer Umfrage über versuchstierkundliche Simulatoren teilzunehmen.

Wir sind ein Team aus Wissenschaftler/innen der Institute für Veterinär-Anatomie, für Tierschutz, Tierverhalten und Versuchstierkunde und für Veterinär-Epidemiologie und Biometrie am Fachbereich Veterinärmedizin der Freien Universität Berlin **und evaluieren in einem Forschungsprojekt alle derzeit kommerziell erhältlichen Ratten- und Maussimulatoren** in versuchstierkundlichen Kursen.

Da Sie als Kursteilnehmer/in am besten bewerten können, wie gut man an einem Simulator trainieren und sich auf die praktischen Übungen am Tier im Kurs vorbereiten kann, bitten wir Sie um **Ihr persönliches Feedback über die Stärken und Schwächen der Simulatoren** mit denen Sie im Kurs trainiert haben.

Danke für Ihre Unterstützung!

Das SimulRATor-Team

Weitere Informationen finden Sie auf www.simulrator.de oder schreiben Sie uns auf kontakt@simulrator.de.

Teil 1 von 2 Rat Simulator A

ID: ____________________

➔ **Bitte merken Sie sich Ihre ID für Teil 2. Danke!**

Hinweis zum Datenschutz: Ihre Teilnahme an der Umfrage ist freiwillig. Die Umfragedaten werden anonymisiert gespeichert und verwaltet. Keinesfalls werden Daten an Dritte weitergegeben. Wir erheben keine personenbezogenen Daten von Ihnen. Es sind keine Rückschlüsse auf Ihre Teilnahme möglich. Nur Projektbeteiligte haben Zugriff auf die von Ihnen innerhalb des Fragebogens gemachten Angaben und sind zur Verschwiegenheit verpflichtet. Die gemachten Angaben werden ausschließlich für Forschungszwecke genutzt. Sie können die Umfrage jederzeit ohne Angabe von Gründen abbrechen.

1. Wie gut gelangen Ihnen die versuchstierkundlichen Methoden an der Rat Simulator A?

Versuchstierkundliche Methoden	Sehr schlecht 1	Ziemlich schlecht 2	Eher schlecht 3	Eher gut 4	Ziemlich gut 5	Sehr gut 6	Methode nicht trainiert.
Handling - Transfer von Käfig zu Käfig	○	○	○	○	○	○	○
Fixieren mit Nackengriff	○	○	○	○	○	○	○
Fixieren mit oberem Schultergriff	○	○	○	○	○	○	○
Fixieren mit mittlerem Schultergriff	○	○	○	○	○	○	○
Fixieren mit unterem Schultergriff	○	○	○	○	○	○	○
Applikation per os mit Sonde	○	○	○	○	○	○	○
Applikation intravenös in die laterale Schwanzvene	○	○	○	○	○	○	○
Blutentnahme aus der lateralen Schwanzvene	○	○	○	○	○	○	○
Sonstiges: ______________________	○	○	○	○	○	○	○
______________________	○	○	○	○	○	○	○

2. Wie gut treffen folgenden Aussagen über das Training an der Rat Simulator A zu?

	Trifft voll und ganz zu 1	Trifft weitgehend zu 2	Trifft eher zu 3	Trifft eher nicht zu 4	Trifft weitgehend nicht zu 5	Trifft ganz und gar nicht zu 6
Die Anzahl an Teilnehmer/innen pro Rat Simulator A war angemessen.	○	○	○	○	○	○
Die Trainingsdauer an der Rat Simulator A war ausreichend.	○	○	○	○	○	○
Die Methoden lassen sich an der Rat Simulator A zufriedenstellend trainieren.	○	○	○	○	○	○
Durch das Training an der Rat Simulator A fühle ich mich für das Kurstraining an der lebenden Ratte besser vorbereitet.	○	○	○	○	○	○

3. Falls es zu materialbedingten Schwierigkeiten bei der Verwendung der Rat Simulator A kam, bitte nennen Sie uns diese.

Versuchstierkundliche Methode: ______________________________

Beschreibung der Komplikation: ______________________________

____

____

Versuchstierkundliche Methode: ______________________________

Beschreibung der Komplikation: ______________________________

____

____

Haben Sie weitere Anmerkungen, Lob oder Kritik? Wir freuen uns über Ihr Feedback!

Herzlichen Dank für Ihre Teilnahme!

Das SimulRATor-Team kontakt@simulrator.de

DEUTSCHES ZENTRUM ZUM SCHUTZ VON VERSUCHSTIEREN

Freie Universität Berlin

Herzlich willkommen bei „SimulRATor“

Wir laden Sie ein, an unserer Umfrage über versuchstierkundliche Simulatoren teilzunehmen.

Wir sind ein Team aus Wissenschaftler/innen der Institute für Veterinär-Anatomie, für Tierschutz, Tierverhalten und Versuchstierkunde und für Veterinär-Epidemiologie und Biometrie am Fachbereich Veterinärmedizin der Freien Universität Berlin **und evaluieren in einem Forschungsprojekt alle derzeit kommerziell erhältlichen Ratten- und Maussimulatoren** in versuchstierkundlichen Kursen.

Da Sie als Kursteilnehmer/in am besten bewerten können, wie gut man an einem Simulator trainieren und sich auf die praktischen Übungen am Tier im Kurs vorbereiten kann, bitten wir Sie um **Ihr persönliches Feedback über die Stärken und Schwächen der Simulatoren** mit denen Sie im Kurs trainiert haben.

Danke für Ihre Unterstützung!

Das SimulRATor-Team

Weitere Informationen finden Sie auf www.simulrator.de oder schreiben Sie uns auf kontakt@simulrator.de.

Teil 2 von 2 Rat Simulator A

ID: ________________

Hinweis zum Datenschutz: Ihre Teilnahme an der Umfrage ist freiwillig. Die Umfragedaten werden anonymisiert gespeichert und verwaltet. Keinesfalls werden Daten an Dritte weitergegeben. Wir erheben keine personenbezogenen Daten von Ihnen. Es sind keine Rückschlüsse auf Ihre Teilnahme möglich. Nur Projektbeteiligte haben Zugriff auf die von Ihnen innerhalb des Fragebogens gemachten Angaben und sind zur Verschwiegenheit verpflichtet. Die gemachten Angaben werden ausschließlich für Forschungszwecke genutzt. Sie können die Umfrage jederzeit ohne Angabe von Gründen abbrechen.

1. Wie gut gelangen Ihnen Handling, Fixieren und Markieren an der lebenden Ratte?

Versuchstierkundliche Methoden	Sehr gut 1	Ziemlich gut 2	Eher gut 3	Eher schlecht 4	Ziemlich schlecht 5	Sehr schlecht 6	Methode nicht trainiert.
Handling - Transfer von Käfig zu Käfig	o	o	o	o	o	o	o
Fixieren mit Nackengriff	o	o	o	o	o	o	o
Fixieren mit oberem Schultergriff	o	o	o	o	o	o	o
Fixieren mit mittlerem Schultergriff	o	o	o	o	o	o	o
Fixieren mit unterem Schultergriff	o	o	o	o	o	o	o
Ohrlochmarkierung	o	o	o	o	o	o	o
Sonstiges: ___________________________________	o	o	o	o	o	o	o
___________________________________	o	o	o	o	o	o	o

2. Wie gut gelangen Ihnen die folgenden versuchstierkundlichen Methoden an der lebenden Ratte?

Bitte kreuzen Sie zusätzlich das Kästchen der letzten Spalte an, wenn die Ratte für die Methode narkotisiert war.

Versuchstierkundliche Methoden	Sehr gut 1	Ziemlich gut 2	Eher gut 3	Eher schlecht 4	Ziemlich schlecht 5	Sehr schlecht 6	Nicht trainiert.	Ratte in Narkose.
Applikation per os ohne Sonde	o	o	o	o	o	o	o	☐
Applikation per os mit Sonde	o	o	o	o	o	o	o	☐
Applikation subkutan Nackenhautfalte	o	o	o	o	o	o	o	☐
Applikation subkutan seitliche Bauchhautfalte (Flanke)	o	o	o	o	o	o	o	☐
Applikation intramuskulär	o	o	o	o	o	o	o	☐
Applikation intraperitoneal	o	o	o	o	o	o	o	☐
Applikation intravenös in die dorsale Penisvene	o	o	o	o	o	o	o	☐
Applikation intravenös in die laterale Schwanzvene	o	o	o	o	o	o	o	☐
Blutentnahme aus der Vena sublingualis	o	o	o	o	o	o	o	☐
Blutentnahme aus retrobulbärem Venenplexus	o	o	o	o	o	o	o	☐
Blutentnahme aus der Vena saphena	o	o	o	o	o	o	o	☐
Blutentnahme aus der lateralen Schwanzvene	o	o	o	o	o	o	o	☐
Blutentnahme kardial	o	o	o	o	o	o	o	☐
Sonstiges: ___________________________________	o	o	o	o	o	o	o	☐
___________________________________	o	o	o	o	o	o	o	☐

3. Welche 3 Methoden an der lebenden Ratte sind Ihrer Meinung nach besonders anspruchsvoll für die/den Durchzuführende(n)?

Bitte nennen Sie 3 Methoden aus der 1.und 2. Frage. Bitte geben Sie diese vollständig an.

1. ____

2. ____

3. ____

4. Bitte wählen Sie aus der unten stehenden Liste die 5 Methoden an der lebenden Ratte, für die Sie ein vorbereitendes Training an Simulatoren für besonders sinnvoll einschätzen.

Sie können bis zu 5 Methoden auswählen.

- ☐ Handling - Transfer von Käfig zu Käfig
- ☐ Fixieren (mit verschiedenen Griffen)
- ☐ Ohrlochmarkierung
- ☐ Applikation per os ohne Sonde
- ☐ Applikation per os mit Sonde
- ☐ Applikation subkutan Nackenhautfalte
- ☐ Applikation subkutan seitliche Bauchhautfalte (Flanke)
- ☐ Applikation intramuskulär
- ☐ Applikation intraperitoneal
- ☐ Applikation intravenös in die dorsale Penisvene
- ☐ Applikation intravenös in die laterale Schwanzvene
- ☐ Blutentnahme aus der Vena sublingualis
- ☐ Blutentnahme aus retrobulbärem Venenplexus
- ☐ Blutentnahme aus der Vena saphena
- ☐ Blutentnahme aus der lateralen Schwanzvene
- ☐ Blutentnahme kardial

5. Würden Sie sich ein Simulator-Training auch für andere Methoden an der Ratte wünschen?

Falls ja, beschreiben Sie uns bitte, welche Methode(n) Ihnen wichtig wäre(n).

Ihr persönliches Feedback zur Rat Simulator A

6. Wie realitätsnah sind folgende Merkmale an der Rat Simulator A im Vergleich zu einer echten Ratte?

Merkmale	Sehr realistisch 1	Ziemlich realistisch 2	Eher realistisch 3	Eher unrealistisch 4	Ziemlich unrealistisch 5	Sehr unrealistisch 6
Gesamterscheinung	o	o	o	o	o	o
Haptik	o	o	o	o	o	o
Verschieblichkeit der Haut im Nacken	o	o	o	o	o	o
Verschieblichkeit der Haut an der seitlichen Bauchwand (Flanke)	o	o	o	o	o	o
Konsistenz der Hautoberfläche	o	o	o	o	o	o
Körpergröße	o	o	o	o	o	o
Gewicht	o	o	o	o	o	o
Körperform	o	o	o	o	o	o
Proportionen	o	o	o	o	o	o

7. Wie realitätsnah war für Sie das Training an der Rat Simulator A im Vergleich zu einer echten Ratte?

Versuchstierkundliche Methoden	Sehr realistisch 1	Ziemlich realistisch 2	Eher realistisch 3	Eher unrealistisch 4	Ziemlich unrealistisch 5	Sehr unrealistisch 6	Methode nicht trainiert.
Handling - Transfer von Käfig zu Käfig	o	o	o	o	o	o	o
Fixieren mit Nackengriff	o	o	o	o	o	o	o
Fixieren mit oberem Schultergriff	o	o	o	o	o	o	o
Fixieren mit mittlerem Schultergriff	o	o	o	o	o	o	o
Fixieren mit unterem Schultergriff	o	o	o	o	o	o	o
Applikation per os mit Sonde	o	o	o	o	o	o	o
Applikation intravenös in die laterale Schwanzvene	o	o	o	o	o	o	o
Blutentnahme aus der lateralen Schwanzvene	o	o	o	o	o	o	o
Sonstiges: ______________________	o	o	o	o	o	o	o
______________________	o	o	o	o	o	o	o

8. Wie gut treffen folgende Aussagen über das Training an der Rat Simulator A zu?

	Trifft voll und ganz zu 1	Trifft weitgehend zu 2	Trifft eher zu 3	Trifft eher nicht zu 4	Trifft weitgehend nicht zu 5	Trifft ganz und gar nicht zu 6
Ich habe mich beim **Handling und Fixieren** an der lebenden Ratte sicherer gefühlt.	o	o	o	o	o	o
Ich habe mich bei den **versuchstierkundlichen Methoden** an der lebenden Ratte sicherer gefühlt.	o	o	o	o	o	o

9. Wie gut hat Sie die Rat Simulator A auf das gesamte Kurstraining an der lebenden Ratte vorbereitet?

Sehr gut	Ziemlich gut	Eher gut	Eher schlecht	Ziemlich schlecht	Sehr schlecht
1	2	3	4	5	6
o	o	o	o	o	o

10. Wie gut hat Sie die Rat Simulator A auf die folgenden Methoden im Kurstraining an der lebenden Ratte vorbereitet?

Versuchstierkundliche Methoden	Sehr gut 1	Ziemlich gut 2	Eher gut 3	Eher schlecht 4	Ziemlich schlecht 5	Sehr schlecht 6	Methode nicht trainiert.
Handling - Transfer von Käfig zu Käfig	o	o	o	o	o	o	o
Fixieren mit Nackengriff	o	o	o	o	o	o	o
Fixieren mit oberem Schultergriff	o	o	o	o	o	o	o
Fixieren mit mittlerem Schultergriff	o	o	o	o	o	o	o
Fixieren mit unterem Schultergriff	o	o	o	o	o	o	o
Applikation per os mit Sonde	o	o	o	o	o	o	o
Applikation intravenös in die laterale Schwanzvene	o	o	o	o	o	o	o
Blutentnahme aus der lateralen Schwanzvene	o	o	o	o	o	o	o
Sonstiges: ___________________________________	o	o	o	o	o	o	o
___________________________________	o	o	o	o	o	o	o

11. Was hat Ihnen an der Rat Simulator A besonders gut gefallen?

12. Was hat Ihnen an der Rat Simulator A nicht gefallen?

13. Was würden Sie an der Rat Simulator A gerne verbessern?

14. Mit welchen Tierarten werden Sie nach bestandenem Kurs in den nächsten 6 Monaten voraussichtlich versuchstierkundlich arbeiten?
Mehrfachantworten sind bei den Tierarten möglich.

- ☐ Ratte
- ☐ Maus
- ☐ Kaninchen
- ☐ Meerschwein
- ☐ Hamster
- ☐ Schwein
- ☐ Schaf
- ☐ Ziege
- ☐ Rind
- ☐ Pferd
- ☐ Hund
- ☐ Katze
- ☐ Sonstiges ___________________________
- ☐ Keine
- ☐ Kann ich nicht beurteilen.

15. Wie alt sind Sie?

________ Jahre

DEUTSCHES ZENTRUM ZUM SCHUTZ VON VERSUCHSTIEREN

Freie Universität Berlin

Herzlich willkommen bei „SimulRATor“

Wir laden Sie ein, an unserer Umfrage über versuchstierkundliche Simulatoren teilzunehmen.

Wir sind ein Team aus Wissenschaftler/innen der Institute für Veterinär-Anatomie, für Tierschutz, Tierverhalten und Versuchstierkunde und für Veterinär-Epidemiologie und Biometrie am Fachbereich Veterinärmedizin der Freien Universität Berlin **und evaluieren in einem Forschungsprojekt alle derzeit kommerziell erhältlichen Ratten- und Maussimulatoren** in versuchstierkundlichen Kursen.

Da Sie als Kursteilnehmer/in am besten bewerten können, wie gut man an einem Simulator trainieren und sich auf die praktischen Übungen am Tier im Kurs vorbereiten kann, bitten wir Sie um **Ihr persönliches Feedback über die Stärken und Schwächen der Simulatoren** mit denen Sie im Kurs trainiert haben.

Danke für Ihre Unterstützung!

Das SimulRATor-Team

Weitere Informationen finden Sie auf www.simulrator.de oder schreiben Sie uns auf kontakt@simulrator.de.

Teil 1 von 2 Rat Simulator B

ID: ____________________

➔ **Bitte merken Sie sich Ihre ID für Teil 2. Danke!**

Hinweis zum Datenschutz: Ihre Teilnahme an der Umfrage ist freiwillig. Die Umfragedaten werden anonymisiert gespeichert und verwaltet. Keinesfalls werden Daten an Dritte weitergegeben. Wir erheben keine personenbezogenen Daten von Ihnen. Es sind keine Rückschlüsse auf Ihre Teilnahme möglich. Nur Projektbeteiligte haben Zugriff auf die von Ihnen innerhalb des Fragebogens gemachten Angaben und sind zur Verschwiegenheit verpflichtet. Die gemachten Angaben werden ausschließlich für Forschungszwecke genutzt. Sie können die Umfrage jederzeit ohne Angabe von Gründen abbrechen.

1. Wie gut gelangen Ihnen die versuchstierkundlichen Methoden an der Rat Simulator B?							
**Versuchstierkundliche Methoden**	**Sehr schlecht 1**	**Ziemlich schlecht 2**	**Eher schlecht 3**	**Eher gut 4**	**Ziemlich gut 5**	**Sehr gut 6**	**Methode nicht trainiert.**
Handling - Transfer von Käfig zu Käfig	o	o	o	o	o	o	o
Fixieren mit Nackengriff	o	o	o	o	o	o	o
Fixieren mit oberem Schultergriff	o	o	o	o	o	o	o
Fixieren mit mittlerem Schultergriff	o	o	o	o	o	o	o
Fixieren mit unterem Schultergriff	o	o	o	o	o	o	o
Applikation per os mit Sonde	o	o	o	o	o	o	o
Applikation intravenös in die laterale Schwanzvene	o	o	o	o	o	o	o
Blutentnahme aus der lateralen Schwanzvene	o	o	o	o	o	o	o
Sonstiges: ______________________	o	o	o	o	o	o	o
______________________	o	o	o	o	o	o	o

2. Wie gut treffen folgenden Aussagen über das Training an der Rat Simulator B zu?						
	**Trifft voll und ganz zu 1**	**Trifft weitgehend zu 2**	**Trifft eher zu 3**	**Trifft eher nicht zu 4**	**Trifft weitgehend nicht zu 5**	**Trifft ganz und gar nicht zu 6**
Die Anzahl an Teilnehmer/innen pro Rat Simulator B war angemessen.	o	o	o	o	o	o
Die Trainingsdauer an der Rat Simulator B war ausreichend.	o	o	o	o	o	o
Die Methoden lassen sich an der Rat Simulator B zufriedenstellend trainieren.	o	o	o	o	o	o
Durch das Training an der Rat Simulator B fühle ich mich für das Kurstraining an der lebenden Ratte besser vorbereitet.	o	o	o	o	o	o

3. Falls es zu materialbedingten Schwierigkeiten bei der Verwendung der Rat Simulator B kam, bitte nennen Sie uns diese.

Versuchstierkundliche Methode: ______________________________

Beschreibung der Komplikation: ______________________________

____

____

Versuchstierkundliche Methode: ______________________________

Beschreibung der Komplikation: ______________________________

____

____

Haben Sie weitere Anmerkungen, Lob oder Kritik? Wir freuen uns über Ihr Feedback!

Herzlichen Dank für Ihre Teilnahme!

Das SimulRATor-Team kontakt@simulrator.de

Herzlich willkommen bei „SimulRATor“

Wir laden Sie ein, an unserer Umfrage über versuchstierkundliche Simulatoren teilzunehmen.

Wir sind ein Team aus Wissenschaftler/innen der Institute für Veterinär-Anatomie, für Tierschutz, Tierverhalten und Versuchstierkunde und für Veterinär-Epidemiologie und Biometrie am Fachbereich Veterinärmedizin der Freien Universität Berlin **und evaluieren in einem Forschungsprojekt alle derzeit kommerziell erhältlichen Ratten- und Maussimulatoren** in versuchstierkundlichen Kursen.

Da Sie als Kursteilnehmer/in am besten bewerten können, wie gut man an einem Simulator trainieren und sich auf die praktischen Übungen am Tier im Kurs vorbereiten kann, bitten wir Sie um **Ihr persönliches Feedback über die Stärken und Schwächen der Simulatoren** mit denen Sie im Kurs trainiert haben.

Danke für Ihre Unterstützung!

Das SimulRATor-Team

Weitere Informationen finden Sie auf www.simulrator.de oder schreiben Sie uns auf kontakt@simulrator.de.

Teil 2 von 2 Rat Simulator B

ID: ________________

Hinweis zum Datenschutz: Ihre Teilnahme an der Umfrage ist freiwillig. Die Umfragedaten werden anonymisiert gespeichert und verwaltet. Keinesfalls werden Daten an Dritte weitergegeben. Wir erheben keine personenbezogenen Daten von Ihnen. Es sind keine Rückschlüsse auf Ihre Teilnahme möglich. Nur Projektbeteiligte haben Zugriff auf die von Ihnen innerhalb des Fragebogens gemachten Angaben und sind zur Verschwiegenheit verpflichtet. Die gemachten Angaben werden ausschließlich für Forschungszwecke genutzt. Sie können die Umfrage jederzeit ohne Angabe von Gründen abbrechen.

1. Wie gut gelangen Ihnen Handling, Fixieren und Markieren an der lebenden Ratte?

Versuchstierkundliche Methoden	Sehr gut 1	Ziemlich gut 2	Eher gut 3	Eher schlecht 4	Ziemlich schlecht 5	Sehr schlecht 6	Methode nicht trainiert.
Handling - Transfer von Käfig zu Käfig	○	○	○	○	○	○	○
Fixieren mit Nackengriff	○	○	○	○	○	○	○
Fixieren mit oberem Schultergriff	○	○	○	○	○	○	○
Fixieren mit mittlerem Schultergriff	○	○	○	○	○	○	○
Fixieren mit unterem Schultergriff	○	○	○	○	○	○	○
Ohrlochmarkierung	○	○	○	○	○	○	○
Sonstiges: ______________________________	○	○	○	○	○	○	○
______________________________	○	○	○	○	○	○	○

2. Wie gut gelangen Ihnen die folgenden versuchstierkundlichen Methoden an der lebenden Ratte?

Bitte kreuzen Sie zusätzlich das Kästchen der letzten Spalte an, wenn die Ratte für die Methode narkotisiert war.

Versuchstierkundliche Methoden	Sehr gut 1	Ziemlich gut 2	Eher gut 3	Eher schlecht 4	Ziemlich schlecht 5	Sehr schlecht 6	Nicht trainiert.	Ratte in Narkose.
Applikation per os ohne Sonde	○	○	○	○	○	○	○	☐
Applikation per os mit Sonde	○	○	○	○	○	○	○	☐
Applikation subkutan Nackenhautfalte	○	○	○	○	○	○	○	☐
Applikation subkutan seitliche Bauchhautfalte (Flanke)	○	○	○	○	○	○	○	☐
Applikation intramuskulär	○	○	○	○	○	○	○	☐
Applikation intraperitoneal	○	○	○	○	○	○	○	☐
Applikation intravenös in die dorsale Penisvene	○	○	○	○	○	○	○	☐
Applikation intravenös in die laterale Schwanzvene	○	○	○	○	○	○	○	☐
Blutentnahme aus der Vena sublingualis	○	○	○	○	○	○	○	☐
Blutentnahme aus retrobulbärem Venenplexus	○	○	○	○	○	○	○	☐
Blutentnahme aus der Vena saphena	○	○	○	○	○	○	○	☐
Blutentnahme aus der lateralen Schwanzvene	○	○	○	○	○	○	○	☐
Blutentnahme kardial	○	○	○	○	○	○	○	☐
Sonstiges: ______________________________	○	○	○	○	○	○	○	☐
______________________________	○	○	○	○	○	○	○	☐

3. Welche 3 Methoden an der lebenden Ratte sind Ihrer Meinung nach besonders anspruchsvoll für die/den Durchzuführende(n)?

Bitte nennen Sie 3 Methoden aus der 1.und 2. Frage. Bitte geben Sie diese vollständig an.

1. ____

2. ____

3. ____

4. Bitte wählen Sie aus der unten stehenden Liste die 5 Methoden an der lebenden Ratte, für die Sie ein vorbereitendes Training an Simulatoren für besonders sinnvoll einschätzen.

Sie können bis zu 5 Methoden auswählen.

- ☐ Handling - Transfer von Käfig zu Käfig
- ☐ Fixieren (mit verschiedenen Griffen)
- ☐ Ohrlochmarkierung
- ☐ Applikation per os ohne Sonde
- ☐ Applikation per os mit Sonde
- ☐ Applikation subkutan Nackenhautfalte
- ☐ Applikation subkutan seitliche Bauchhautfalte (Flanke)
- ☐ Applikation intramuskulär
- ☐ Applikation intraperitoneal
- ☐ Applikation intravenös in die dorsale Penisvene
- ☐ Applikation intravenös in die laterale Schwanzvene
- ☐ Blutentnahme aus der Vena sublingualis
- ☐ Blutentnahme aus retrobulbärem Venenplexus
- ☐ Blutentnahme aus der Vena saphena
- ☐ Blutentnahme aus der lateralen Schwanzvene
- ☐ Blutentnahme kardial

5. Würden Sie sich ein Simulator-Training auch für andere Methoden an der Ratte wünschen?

Falls ja, beschreiben Sie uns bitte, welche Methode(n) Ihnen wichtig wäre(n).

Ihr persönliches Feedback zur Rat Simulator B

6. Wie realitätsnah sind folgende Merkmale an der Rat Simulator B im Vergleich zu einer echten Ratte?

Merkmale	Sehr realistisch 1	Ziemlich realistisch 2	Eher realistisch 3	Eher unrealistisch 4	Ziemlich unrealistisch 5	Sehr unrealistisch 6
Gesamterscheinung	○	○	○	○	○	○
Haptik	○	○	○	○	○	○
Verschieblichkeit der Haut im Nacken	○	○	○	○	○	○
Verschieblichkeit der Haut an der seitlichen Bauchwand (Flanke)	○	○	○	○	○	○
Konsistenz der Hautoberfläche	○	○	○	○	○	○
Körpergröße	○	○	○	○	○	○
Gewicht	○	○	○	○	○	○
Körperform	○	○	○	○	○	○
Proportionen	○	○	○	○	○	○

7. Wie realitätsnah war für Sie das Training an der Rat Simulator B im Vergleich zu einer echten Ratte?

Versuchstierkundliche Methoden	Sehr realistisch 1	Ziemlich realistisch 2	Eher realistisch 3	Eher unrealistisch 4	Ziemlich unrealistisch 5	Sehr unrealistisch 6	Methode nicht trainiert.
Handling - Transfer von Käfig zu Käfig	○	○	○	○	○	○	○
Fixieren mit Nackengriff	○	○	○	○	○	○	○
Fixieren mit oberem Schultergriff	○	○	○	○	○	○	○
Fixieren mit mittlerem Schultergriff	○	○	○	○	○	○	○
Fixieren mit unterem Schultergriff	○	○	○	○	○	○	○
Applikation per os mit Sonde	○	○	○	○	○	○	○
Applikation intravenös in die laterale Schwanzvene	○	○	○	○	○	○	○
Blutentnahme aus der lateralen Schwanzvene	○	○	○	○	○	○	○
Sonstiges: ______________________	○	○	○	○	○	○	○
______________________	○	○	○	○	○	○	○

8. Wie gut treffen folgende Aussagen über das Training an der Rat Simulator B zu?

	Trifft voll und ganz zu 1	Trifft weitgehend zu 2	Trifft eher zu 3	Trifft eher nicht zu 4	Trifft weitgehend nicht zu 5	Trifft ganz und gar nicht zu 6
Ich habe mich beim **Handling und Fixieren** an der lebenden Ratte sicherer gefühlt.	○	○	○	○	○	○
Ich habe mich bei den **versuchstierkundlichen Methoden** an der lebenden Ratte sicherer gefühlt.	○	○	○	○	○	○

9. Wie gut hat Sie die Rat Simulator B auf das gesamte Kurstraining an der lebenden Ratte vorbereitet?					
Sehr gut	Ziemlich gut	Eher gut	Eher schlecht	Ziemlich schlecht	Sehr schlecht
1	2	3	4	5	6
○	○	○	○	○	○

10. Wie gut hat Sie die Rat Simulator B auf die folgenden Methoden im Kurstraining an der lebenden Ratte vorbereitet?

Versuchstierkundliche Methoden	Sehr gut 1	Ziemlich gut 2	Eher gut 3	Eher schlecht 4	Ziemlich schlecht 5	Sehr schlecht 6	Methode nicht trainiert.
Handling - Transfer von Käfig zu Käfig	○	○	○	○	○	○	○
Fixieren mit Nackengriff	○	○	○	○	○	○	○
Fixieren mit oberem Schultergriff	○	○	○	○	○	○	○
Fixieren mit mittlerem Schultergriff	○	○	○	○	○	○	○
Fixieren mit unterem Schultergriff	○	○	○	○	○	○	○
Applikation per os mit Sonde	○	○	○	○	○	○	○
Applikation intravenös in die laterale Schwanzvene	○	○	○	○	○	○	○
Blutentnahme aus der lateralen Schwanzvene	○	○	○	○	○	○	○
Sonstiges: ___________________________________	○	○	○	○	○	○	○
___________________________________	○	○	○	○	○	○	○

11. Was hat Ihnen an der Rat Simulator B besonders gut gefallen?

12. Was hat Ihnen an der Rat Simulator B nicht gefallen?

13. Was würden Sie an der Rat Simulator B gerne verbessern?

14. Mit welchen Tierarten werden Sie nach bestandenem Kurs in den nächsten 6 Monaten voraussichtlich versuchstierkundlich arbeiten?
Mehrfachantworten sind bei den Tierarten möglich.

- ☐ Ratte
- ☐ Maus
- ☐ Kaninchen
- ☐ Meerschwein
- ☐ Hamster
- ☐ Schwein
- ☐ Schaf
- ☐ Ziege
- ☐ Rind
- ☐ Pferd
- ☐ Hund
- ☐ Katze
- ☐ Sonstiges ___________________________
- ☐ Keine
- ☐ Kann ich nicht beurteilen.

15. Wie alt sind Sie?

________ Jahre

Bitte wenden.

16. Welches Geschlecht haben Sie?

- □ Männlich
- □ Weiblich
- □ Divers
- □ Keine Angabe

17. Bitte kreuzen Sie Ihren höchsten abgeschlossenen Bildungsabschluss an.
Nur eine Antwort ist möglich

- □ (Noch) kein beruflicher Abschluss
- □ Abgeschlossene berufliche-/ betriebliche-/ schulische Ausbildung
- □ Abgeschlossene Ausbildung an Fachschule, Meister-, Technikerschule, Berufs- oder Fachakademie
- □ Fachhochschulabschluss
- □ Bachelorabschluss
- □ Master-, Magister-, Diplom- oder Staatsexamensabschluss
- □ Promotion
- □ Sonstiges: ____

18. Bitte vervollständigen Sie den Satz:
Nur eine Antwort ist möglich.

Ich besuche den Kurs als …

- □ …wissenschaftlich Beschäftigte/r.
- □ …wissenschaftlich-technische/r Mitarbeiter/in.
- □ …Auszubildende/r.
- □ …Studierende/r.
- □ …Sonstiges: ____.

19. Aus welcher Fachrichtung kommen Sie?
Nur eine Antwort ist möglich.

- □ Humanmedizin
- □ Veterinärmedizin
- □ Zahnmedizin
- □ Pharmazie
- □ Biologie
- □ Chemie
- □ Physik
- □ Biotechnologie
- □ Ernährungswissenschaft
- □ Sonstiges: ____

20. Hatten Sie vor dem Kurs bereits Erfahrung …
Nur eine Antwort ist möglich.

…im Handling von Ratten?

- □ Ja, viel.
- □ Ja, etwas.
- □ Nein, gar keine.

21. Hatten Sie vor dem Kurs bereits Erfahrung …
Nur eine Antwort ist möglich.

…im Handling von Mäusen?

- □ Ja, viel.
- □ Ja, etwas.
- □ Nein, gar keine.

22. Haben Sie vor dem Kurs schon einmal mit Simulatoren gearbeitet?
Mehrere Antworten für Simulatoren sind möglich. Bitte beschreiben Sie das Modell kurz.

- □ Ja, …
  - □ …mit Ratten-Simulatoren: Modell: ____
  - □ …mit Maus-Simulatoren: Modell: ____
  - □ …mit sonstigen Simulatoren: Modell: ____
- □ Nein.

Haben Sie weitere Anmerkungen, Lob oder Kritik? Wir freuen uns über Ihr Feedback!

Herzlichen Dank für Ihre Teilnahme!

Das SimulRATor-Team

web: www.simulator.de mail: kontakt@simulrator.de

Herzlich willkommen bei „SimulRATor“

Wir laden Sie ein, an unserer Umfrage über versuchstierkundliche Simulatoren teilzunehmen.

Wir sind ein Team aus Wissenschaftler/innen der Institute für Veterinär-Anatomie, für Tierschutz, Tierverhalten und Versuchstierkunde und für Veterinär-Epidemiologie und Biometrie am Fachbereich Veterinärmedizin der Freien Universität Berlin **und evaluieren in einem Forschungsprojekt alle derzeit kommerziell erhältlichen Ratten- und Maussimulatoren** in versuchstierkundlichen Kursen.

Da Sie als Kursteilnehmer/in am besten bewerten können, wie gut man an einem Simulator trainieren und sich auf die praktischen Übungen am Tier im Kurs vorbereiten kann, bitten wir Sie um **Ihr persönliches Feedback über die Stärken und Schwächen der Simulatoren** mit denen Sie im Kurs trainiert haben.

Danke für Ihre Unterstützung!

Das SimulRATor-Team

Weitere Informationen finden Sie auf www.simulrator.de oder schreiben Sie uns auf kontakt@simulrator.de.

Teil 1 von 2 Rat Simulator C

ID: ________________

➔ **Bitte merken Sie sich Ihre ID für Teil 2. Danke!**

Hinweis zum Datenschutz: Ihre Teilnahme an der Umfrage ist freiwillig. Die Umfragedaten werden anonymisiert gespeichert und verwaltet. Keinesfalls werden Daten an Dritte weitergegeben. Wir erheben keine personenbezogenen Daten von Ihnen. Es sind keine Rückschlüsse auf Ihre Teilnahme möglich. Nur Projektbeteiligte haben Zugriff auf die von Ihnen innerhalb des Fragebogens gemachten Angaben und sind zur Verschwiegenheit verpflichtet. Die gemachten Angaben werden ausschließlich für Forschungszwecke genutzt. Sie können die Umfrage jederzeit ohne Angabe von Gründen abbrechen.

1. Wie gut gelangen Ihnen die versuchstierkundlichen Methoden an der Rat Simulator C?							
**Versuchstierkundliche Methoden**	Sehr schlecht 1	Ziemlich schlecht 2	Eher schlecht 3	Eher gut 4	Ziemlich gut 5	Sehr gut 6	Methode nicht trainiert.
Handling - Transfer von Käfig zu Käfig	o	o	o	o	o	o	o
Fixieren mit Nackengriff	o	o	o	o	o	o	o
Fixieren mit oberem Schultergriff	o	o	o	o	o	o	o
Fixieren mit mittlerem Schultergriff	o	o	o	o	o	o	o
Fixieren mit unterem Schultergriff	o	o	o	o	o	o	o
Applikation intravenös in die laterale Schwanzvene	o	o	o	o	o	o	o
Blutentnahme aus der Vena saphena	o	o	o	o	o	o	o
Blutentnahme aus der lateralen Schwanzvene	o	o	o	o	o	o	o
Blutentnahme kardial	o	o	o	o	o	o	o
Sonstiges: ______________________	o	o	o	o	o	o	o
______________________	o	o	o	o	o	o	o

2. Wie gut treffen folgenden Aussagen über das Training an der Rat Simulator C zu?						
	Trifft voll und ganz zu 1	Trifft weitgehend zu 2	Trifft eher zu 3	Trifft eher nicht zu 4	Trifft weitgehend nicht zu 5	Trifft ganz und gar nicht zu 6
Die Anzahl an Teilnehmer/innen pro Rat Simulator C war angemessen.	o	o	o	o	o	o
Die Trainingsdauer an der Rat Simulator C war ausreichend.	o	o	o	o	o	o
Die Methoden lassen sich an der Rat Simulator C zufriedenstellend trainieren.	o	o	o	o	o	o
Durch das Training an der Rat Simulator C fühle ich mich für das Kurstraining an der lebenden Ratte besser vorbereitet.	o	o	o	o	o	o

3. Falls es zu materialbedingten Schwierigkeiten bei der Verwendung der Rat Simulator C kam, bitte nennen Sie uns diese.

Versuchstierkundliche Methode: ______________________________

Beschreibung der Komplikation: ______________________________

____

____

Versuchstierkundliche Methode: ______________________________

Beschreibung der Komplikation: ______________________________

____

____

Haben Sie weitere Anmerkungen, Lob oder Kritik? Wir freuen uns über Ihr Feedback!

Herzlichen Dank für Ihre Teilnahme!

Das SimulRATor-Team kontakt@simulrator.de

Herzlich willkommen bei „SimulRATor“

Wir laden Sie ein, an unserer Umfrage über versuchstierkundliche Simulatoren teilzunehmen.

Wir sind ein Team aus Wissenschaftler/innen der Institute für Veterinär-Anatomie, für Tierschutz, Tierverhalten und Versuchstierkunde und für Veterinär-Epidemiologie und Biometrie am Fachbereich Veterinärmedizin der Freien Universität Berlin **und evaluieren in einem Forschungsprojekt alle derzeit kommerziell erhältlichen Ratten- und Maussimulatoren** in versuchstierkundlichen Kursen.

Da Sie als Kursteilnehmer/in am besten bewerten können, wie gut man an einem Simulator trainieren und sich auf die praktischen Übungen am Tier im Kurs vorbereiten kann, bitten wir Sie um **Ihr persönliches Feedback über die Stärken und Schwächen der Simulatoren** mit denen Sie im Kurs trainiert haben.

Danke für Ihre Unterstützung!

Das SimulRATor-Team

Weitere Informationen finden Sie auf www.simulrator.de oder schreiben Sie uns auf kontakt@simulrator.de.

Teil 2 von 2 Rat Simulator C

ID: ________________

Hinweis zum Datenschutz: Ihre Teilnahme an der Umfrage ist freiwillig. Die Umfragedaten werden anonymisiert gespeichert und verwaltet. Keinesfalls werden Daten an Dritte weitergegeben. Wir erheben keine personenbezogenen Daten von Ihnen. Es sind keine Rückschlüsse auf Ihre Teilnahme möglich. Nur Projektbeteiligte haben Zugriff auf die von Ihnen innerhalb des Fragebogens gemachten Angaben und sind zur Verschwiegenheit verpflichtet. Die gemachten Angaben werden ausschließlich für Forschungszwecke genutzt. Sie können die Umfrage jederzeit ohne Angabe von Gründen abbrechen.

1. Wie gut gelangen Ihnen Handling, Fixieren und Markieren an der lebenden Ratte?

**Versuchstierkundliche Methoden**	**Sehr gut 1**	**Ziemlich gut 2**	**Eher gut 3**	**Eher schlecht 4**	**Ziemlich schlecht 5**	**Sehr schlecht 6**	**Methode nicht trainiert.**
Handling - Transfer von Käfig zu Käfig	O	O	O	O	O	O	O
Fixieren mit Nackengriff	O	O	O	O	O	O	O
Fixieren mit oberem Schultergriff	O	O	O	O	O	O	O
Fixieren mit mittlerem Schultergriff	O	O	O	O	O	O	O
Fixieren mit unterem Schultergriff	O	O	O	O	O	O	O
Ohrlochmarkierung	O	O	O	O	O	O	O
Sonstiges: ___________________________	O	O	O	O	O	O	O
___________________________	O	O	O	O	O	O	O

2. Wie gut gelangen Ihnen die folgenden versuchstierkundlichen Methoden an der lebenden Ratte?
Bitte kreuzen Sie zusätzlich das Kästchen der letzten Spalte an, wenn die Ratte für die Methode narkotisiert war.

**Versuchstierkundliche Methoden**	**Sehr gut 1**	**Ziemlich gut 2**	**Eher gut 3**	**Eher schlecht 4**	**Ziemlich schlecht 5**	**Sehr schlecht 6**	**Nicht trainiert.**	**Ratte in Narkose.**
Applikation per os ohne Sonde	O	O	O	O	O	O	O	☐
Applikation per os mit Sonde	O	O	O	O	O	O	O	☐
Applikation subkutan Nackenhautfalte	O	O	O	O	O	O	O	☐
Applikation subkutan seitliche Bauchhautfalte (Flanke)	O	O	O	O	O	O	O	☐
Applikation intramuskulär	O	O	O	O	O	O	O	☐
Applikation intraperitoneal	O	O	O	O	O	O	O	☐
Applikation intravenös in die dorsale Penisvene	O	O	O	O	O	O	O	☐
Applikation intravenös in die laterale Schwanzvene	O	O	O	O	O	O	O	☐
Blutentnahme aus der Vena sublingualis	O	O	O	O	O	O	O	☐
Blutentnahme aus retrobulbärem Venenplexus	O	O	O	O	O	O	O	☐
Blutentnahme aus der Vena saphena	O	O	O	O	O	O	O	☐
Blutentnahme aus der lateralen Schwanzvene	O	O	O	O	O	O	O	☐
Blutentnahme kardial	O	O	O	O	O	O	O	☐
Sonstiges: ___________________________	O	O	O	O	O	O	O	☐
___________________________	O	O	O	O	O	O	O	☐

3. Welche <u>3 Methoden</u> an der lebenden Ratte sind Ihrer Meinung nach besonders anspruchsvoll für die/den Durchzuführende(n)?

Bitte nennen Sie 3 Methoden aus der <u>1.und 2. Frage</u>. Bitte geben Sie diese <u>vollständig</u> an.

1. ____

2. ____

3. ____

4. Bitte wählen Sie aus der unten stehenden Liste <u>die 5 Methoden</u> an der lebenden Ratte, für die Sie ein vorbereitendes Training an Simulatoren für besonders sinnvoll einschätzen.

Sie können bis zu <u>5 Methoden</u> auswählen.

☐ Handling - Transfer von Käfig zu Käfig
☐ Fixieren (mit verschiedenen Griffen)
☐ Ohrlochmarkierung
☐ Applikation per os ohne Sonde
☐ Applikation per os mit Sonde
☐ Applikation subkutan Nackenhautfalte
☐ Applikation subkutan seitliche Bauchhautfalte (Flanke)
☐ Applikation intramuskulär
☐ Applikation intraperitoneal
☐ Applikation intravenös in die dorsale Penisvene
☐ Applikation intravenös in die laterale Schwanzvene
☐ Blutentnahme aus der Vena sublingualis
☐ Blutentnahme aus retrobulbärem Venenplexus
☐ Blutentnahme aus der Vena saphena
☐ Blutentnahme aus der lateralen Schwanzvene
☐ Blutentnahme kardial

5. Würden Sie sich ein Simulator-Training auch für <u>andere</u> Methoden an der Ratte wünschen?
Falls ja, beschreiben Sie uns bitte, welche Methode(n) Ihnen wichtig wäre(n).

Ihr persönliches Feedback zur Rat Simulator C

6. Wie realitätsnah sind folgende Merkmale an der Rat Simulator C im Vergleich zu einer echten Ratte?

**Merkmale**	**Sehr realistisch 1**	**Ziemlich realistisch 2**	**Eher realistisch 3**	**Eher unrealistisch 4**	**Ziemlich unrealistisch 5**	**Sehr unrealistisch 6**
Gesamterscheinung	○	○	○	○	○	○
Haptik	○	○	○	○	○	○
Verschieblichkeit der Haut im Nacken	○	○	○	○	○	○
Verschieblichkeit der Haut an der seitlichen Bauchwand (Flanke)	○	○	○	○	○	○
Konsistenz der Hautoberfläche	○	○	○	○	○	○
Körpergröße	○	○	○	○	○	○
Gewicht	○	○	○	○	○	○
Körperform	○	○	○	○	○	○
Proportionen	○	○	○	○	○	○

7. Wie realitätsnah war für Sie das Training an der Rat Simulator C im Vergleich zu einer echten Ratte?

**Versuchstierkundliche Methoden**	**Sehr realistisch 1**	**Ziemlich realistisch 2**	**Eher realistisch 3**	**Eher unrealistisch 4**	**Ziemlich unrealistisch 5**	**Sehr unrealistisch 6**	**Methode nicht trainiert.**
Handling - Transfer von Käfig zu Käfig	○	○	○	○	○	○	○
Fixieren mit Nackengriff	○	○	○	○	○	○	○
Fixieren mit oberem Schultergriff	○	○	○	○	○	○	○
Fixieren mit mittlerem Schultergriff	○	○	○	○	○	○	○
Fixieren mit unterem Schultergriff	○	○	○	○	○	○	○
Applikation intravenös in die laterale Schwanzvene	○	○	○	○	○	○	○
Blutentnahme aus der Vena saphena	○	○	○	○	○	○	○
Blutentnahme aus der lateralen Schwanzvene	○	○	○	○	○	○	○
Blutentnahme kardial	○	○	○	○	○	○	○
Sonstiges: ________________________________	○	○	○	○	○	○	○
________________________________	○	○	○	○	○	○	○

8. Wie gut treffen folgende Aussagen über das Training an der Rat Simulator C zu?

	**Trifft voll und ganz zu 1**	**Trifft weitgehend zu 2**	**Trifft eher zu 3**	**Trifft eher nicht zu 4**	**Trifft weitgehend nicht zu 5**	**Trifft ganz und gar nicht zu 6**
Ich habe mich beim **Handling und Fixieren** an der lebenden Ratte sicherer gefühlt.	○	○	○	○	○	○
Ich habe mich bei den **versuchstierkundlichen Methoden** an der lebenden Ratte sicherer gefühlt.	○	○	○	○	○	○

9. Wie gut hat Sie die Rat Simulator C auf das gesamte Kurstraining an der lebenden Ratte vorbereitet?

Sehr gut	Ziemlich gut	Eher gut	Eher schlecht	Ziemlich schlecht	Sehr schlecht
1	2	3	4	5	6
o	o	o	o	o	o

10. Wie gut hat Sie die Rat Simulator C auf die folgenden Methoden im Kurstraining an der lebenden Ratte vorbereitet?

Versuchstierkundliche Methoden	Sehr gut 1	Ziemlich gut 2	Eher gut 3	Eher schlecht 4	Ziemlich schlecht 5	Sehr schlecht 6	Methode nicht trainiert.
Handling - Transfer von Käfig zu Käfig	o	o	o	o	o	o	o
Fixieren mit Nackengriff	o	o	o	o	o	o	o
Fixieren mit oberem Schultergriff	o	o	o	o	o	o	o
Fixieren mit mittlerem Schultergriff	o	o	o	o	o	o	o
Fixieren mit unterem Schultergriff	o	o	o	o	o	o	o
Applikation intravenös in die laterale Schwanzvene	o	o	o	o	o	o	o
Blutentnahme aus der Vena saphena	o	o	o	o	o	o	o
Blutentnahme aus der lateralen Schwanzvene	o	o	o	o	o	o	o
Blutentnahme kardial	o	o	o	o	o	o	o
Sonstiges: ________________________________	o	o	o	o	o	o	o
________________________________	o	o	o	o	o	o	o

11. Was hat Ihnen an der Rat Simulator C besonders gut gefallen?

12. Was hat Ihnen an der Rat Simulator C nicht gefallen?

13. Was würden Sie an der Rat Simulator C gerne verbessern?

14. Mit welchen Tierarten werden Sie nach bestandenem Kurs in den nächsten 6 Monaten voraussichtlich versuchstierkundlich arbeiten?
Mehrfachantworten sind bei den Tierarten möglich.

- ☐ Ratte
- ☐ Maus
- ☐ Kaninchen
- ☐ Meerschwein
- ☐ Hamster
- ☐ Schwein
- ☐ Schaf
- ☐ Ziege
- ☐ Rind
- ☐ Pferd
- ☐ Hund
- ☐ Katze
- ☐ Sonstiges ________________________
- ☐ Keine
- ☐ Kann ich nicht beurteilen.

15. Wie alt sind Sie?

________ Jahre

16. Welches Geschlecht haben Sie?

- ☐ Männlich
- ☐ Weiblich
- ☐ Divers
- ☐ Keine Angabe

17. Bitte kreuzen Sie Ihren <u>höchsten abgeschlossenen</u> Bildungsabschluss an.
Nur eine Antwort ist möglich

- ☐ (Noch) kein beruflicher Abschluss
- ☐ Abgeschlossene berufliche-/ betriebliche-/ schulische Ausbildung
- ☐ Abgeschlossene Ausbildung an Fachschule, Meister-, Technikerschule, Berufs- oder Fachakademie
- ☐ Fachhochschulabschluss
- ☐ Bachelorabschluss
- ☐ Master-, Magister-, Diplom- oder Staatsexamensabschluss
- ☐ Promotion
- ☐ Sonstiges: ____

18. Bitte vervollständigen Sie den Satz:
Nur eine Antwort ist möglich.

Ich besuche den Kurs als …

- ☐ …wissenschaftlich Beschäftigte/r.
- ☐ …wissenschaftlich-technische/r Mitarbeiter/in.
- ☐ …Auszubildende/r.
- ☐ …Studierende/r.
- ☐ …Sonstiges: ____.

19. Aus welcher Fachrichtung kommen Sie?
Nur eine Antwort ist möglich.

- ☐ Humanmedizin
- ☐ Veterinärmedizin
- ☐ Zahnmedizin
- ☐ Pharmazie
- ☐ Biologie
- ☐ Chemie
- ☐ Physik
- ☐ Biotechnologie
- ☐ Ernährungswissenschaft
- ☐ Sonstiges: ____

20. Hatten Sie vor dem Kurs bereits Erfahrung …
Nur eine Antwort ist möglich.

…im Handling von <u>Ratten</u>?

- ☐ Ja, viel.
- ☐ Ja, etwas.
- ☐ Nein, gar keine.

21. Hatten Sie vor dem Kurs bereits Erfahrung …
Nur eine Antwort ist möglich.

…im Handling von <u>Mäusen</u>?

- ☐ Ja, viel.
- ☐ Ja, etwas.
- ☐ Nein, gar keine.

22. Haben Sie vor dem Kurs schon einmal mit Simulatoren gearbeitet?
Mehrere Antworten für Simulatoren sind möglich. Bitte beschreiben Sie das Modell kurz.

- ☐ Ja, …
  - ☐ …mit Ratten-Simulatoren: Modell: _______________________________________
  - ☐ …mit Maus-Simulatoren: Modell: _______________________________________
  - ☐ …mit sonstigen Simulatoren: Modell: _______________________________________
- ☐ Nein.

Haben Sie weitere Anmerkungen, Lob oder Kritik? Wir freuen uns über Ihr Feedback!

Herzlichen Dank für Ihre Teilnahme!

Das SimulRATor-Team

web: www.simulator.de mail: kontakt@simulrator.de

Herzlich willkommen bei „SimulRATor“

Wir laden Sie ein, an unserer Umfrage über versuchstierkundliche Simulatoren teilzunehmen.

Wir sind ein Team aus Wissenschaftler/innen der Institute für Veterinär-Anatomie, für Tierschutz, Tierverhalten und Versuchstierkunde und für Veterinär-Epidemiologie und Biometrie am Fachbereich Veterinärmedizin der Freien Universität Berlin **und evaluieren in einem Forschungsprojekt alle derzeit kommerziell erhältlichen Ratten- und Maussimulatoren** in versuchstierkundlichen Kursen.

Da Sie als Kursteilnehmer/in am besten bewerten können, wie gut man an einem Simulator trainieren und sich auf die praktischen Übungen am Tier im Kurs vorbereiten kann, bitten wir Sie um **Ihr persönliches Feedback über die Stärken und Schwächen der Simulatoren** mit denen Sie im Kurs trainiert haben.

Danke für Ihre Unterstützung!

Das SimulRATor-Team

Weitere Informationen finden Sie auf www.simulrator.de oder schreiben Sie uns auf kontakt@simulrator.de.

Teil 1 von 2 Rat Simulator D

ID: ____________________

➔ **Bitte merken Sie sich Ihre ID für Teil 2. Danke!**

Hinweis zum Datenschutz: Ihre Teilnahme an der Umfrage ist freiwillig. Die Umfragedaten werden anonymisiert gespeichert und verwaltet. Keinesfalls werden Daten an Dritte weitergegeben. Wir erheben keine personenbezogenen Daten von Ihnen. Es sind keine Rückschlüsse auf Ihre Teilnahme möglich. Nur Projektbeteiligte haben Zugriff auf die von Ihnen innerhalb des Fragebogens gemachten Angaben und sind zur Verschwiegenheit verpflichtet. Die gemachten Angaben werden ausschließlich für Forschungszwecke genutzt. Sie können die Umfrage jederzeit ohne Angabe von Gründen abbrechen.

1. Wie gut gelangen Ihnen die versuchstierkundlichen Methoden an der Rat Simulator D?

Versuchstierkundliche Methoden	Sehr schlecht 1	Ziemlich schlecht 2	Eher schlecht 3	Eher gut 4	Ziemlich gut 5	Sehr gut 6	Methode nicht trainiert.
Handling - Transfer von Käfig zu Käfig	o	o	o	o	o	o	o
Fixieren mit Nackengriff	o	o	o	o	o	o	o
Fixieren mit oberem Schultergriff	o	o	o	o	o	o	o
Fixieren mit mittlerem Schultergriff	o	o	o	o	o	o	o
Fixieren mit unterem Schultergriff	o	o	o	o	o	o	o
Ohrlochmarkierung	o	o	o	o	o	o	o
Applikation intravenös in die laterale Schwanzvene	o	o	o	o	o	o	o
Blutentnahme aus der lateralen Schwanzvene	o	o	o	o	o	o	o
Sonstiges: ______________________________	o	o	o	o	o	o	o
______________________________	o	o	o	o	o	o	o

2. Wie gut treffen folgenden Aussagen über das Training an der Rat Simulator D zu?

	Trifft voll und ganz zu 1	Trifft weitgehend zu 2	Trifft eher zu 3	Trifft eher nicht zu 4	Trifft weitgehend nicht zu 5	Trifft ganz und gar nicht zu 6
Die Anzahl an Teilnehmer/innen pro Rat Simulator D war angemessen.	o	o	o	o	o	o
Die Trainingsdauer an der Rat Simulator D war ausreichend.	o	o	o	o	o	o
Die Methoden lassen sich an der Rat Simulator D zufriedenstellend trainieren.	o	o	o	o	o	o
Durch das Training an der Rat Simulator D fühle ich mich für das Kurstraining an der lebenden Ratte besser vorbereitet.	o	o	o	o	o	o

3. Falls es zu materialbedingten Schwierigkeiten bei der Verwendung der Rat Simulator D kam, bitte nennen Sie uns diese.

Versuchstierkundliche Methode: ______________________________

Beschreibung der Komplikation: ______________________________

____

____

Versuchstierkundliche Methode: ______________________________

Beschreibung der Komplikation: ______________________________

____

____

Haben Sie weitere Anmerkungen, Lob oder Kritik? Wir freuen uns über Ihr Feedback!

Herzlichen Dank für Ihre Teilnahme!

Das SimulRATor-Team kontakt@simulrator.de

DEUTSCHES ZENTRUM ZUM SCHUTZ VON VERSUCHSTIEREN

Freie Universität Berlin

Herzlich willkommen bei „SimulRATor“

Wir laden Sie ein, an unserer Umfrage über versuchstierkundliche Simulatoren teilzunehmen.

Wir sind ein Team aus Wissenschaftler/innen der Institute für Veterinär-Anatomie, für Tierschutz, Tierverhalten und Versuchstierkunde und für Veterinär-Epidemiologie und Biometrie am Fachbereich Veterinärmedizin der Freien Universität Berlin **und evaluieren in einem Forschungsprojekt alle derzeit kommerziell erhältlichen Ratten- und Maussimulatoren** in versuchstierkundlichen Kursen.

Da Sie als Kursteilnehmer/in am besten bewerten können, wie gut man an einem Simulator trainieren und sich auf die praktischen Übungen am Tier im Kurs vorbereiten kann, bitten wir Sie um **Ihr persönliches Feedback über die Stärken und Schwächen der Simulatoren** mit denen Sie im Kurs trainiert haben.

Danke für Ihre Unterstützung!

Das SimulRATor-Team

Weitere Informationen finden Sie auf www.simulrator.de oder schreiben Sie uns auf kontakt@simulrator.de.

Teil 2 von 2 Rat Simualtor D

ID: ________________

Hinweis zum Datenschutz: Ihre Teilnahme an der Umfrage ist freiwillig. Die Umfragedaten werden anonymisiert gespeichert und verwaltet. Keinesfalls werden Daten an Dritte weitergegeben. Wir erheben keine personenbezogenen Daten von Ihnen. Es sind keine Rückschlüsse auf Ihre Teilnahme möglich. Nur Projektbeteiligte haben Zugriff auf die von Ihnen innerhalb des Fragebogens gemachten Angaben und sind zur Verschwiegenheit verpflichtet. Die gemachten Angaben werden ausschließlich für Forschungszwecke genutzt. Sie können die Umfrage jederzeit ohne Angabe von Gründen abbrechen.

1. Wie gut gelangen Ihnen Handling, Fixieren und Markieren an der lebenden Ratte?

Versuchstierkundliche Methoden	Sehr gut 1	Ziemlich gut 2	Eher gut 3	Eher schlecht 4	Ziemlich schlecht 5	Sehr schlecht 6	Methode nicht trainiert.
Handling - Transfer von Käfig zu Käfig	○	○	○	○	○	○	○
Fixieren mit Nackengriff	○	○	○	○	○	○	○
Fixieren mit oberem Schultergriff	○	○	○	○	○	○	○
Fixieren mit mittlerem Schultergriff	○	○	○	○	○	○	○
Fixieren mit unterem Schultergriff	○	○	○	○	○	○	○
Ohrlochmarkierung	○	○	○	○	○	○	○
Sonstiges: ___________________________________	○	○	○	○	○	○	○
___________________________________	○	○	○	○	○	○	○

2. Wie gut gelangen Ihnen die folgenden versuchstierkundlichen Methoden an der lebenden Ratte?

Bitte kreuzen Sie zusätzlich das Kästchen der letzten Spalte an, wenn die Ratte für die Methode narkotisiert war.

Versuchstierkundliche Methoden	Sehr gut 1	Ziemlich gut 2	Eher gut 3	Eher schlecht 4	Ziemlich schlecht 5	Sehr schlecht 6	Nicht trainiert.	Ratte in Narkose.
Applikation per os ohne Sonde	○	○	○	○	○	○	○	☐
Applikation per os mit Sonde	○	○	○	○	○	○	○	☐
Applikation subkutan Nackenhautfalte	○	○	○	○	○	○	○	☐
Applikation subkutan seitliche Bauchhautfalte (Flanke)	○	○	○	○	○	○	○	☐
Applikation intramuskulär	○	○	○	○	○	○	○	☐
Applikation intraperitoneal	○	○	○	○	○	○	○	☐
Applikation intravenös in die dorsale Penisvene	○	○	○	○	○	○	○	☐
Applikation intravenös in die laterale Schwanzvene	○	○	○	○	○	○	○	☐
Blutentnahme aus der Vena sublingualis	○	○	○	○	○	○	○	☐
Blutentnahme aus retrobulbärem Venenplexus	○	○	○	○	○	○	○	☐
Blutentnahme aus der Vena saphena	○	○	○	○	○	○	○	☐
Blutentnahme aus der lateralen Schwanzvene	○	○	○	○	○	○	○	☐
Blutentnahme kardial	○	○	○	○	○	○	○	☐
Sonstiges: ___________________________________	○	○	○	○	○	○	○	☐
___________________________________	○	○	○	○	○	○	○	☐

3. Welche <u>3 Methoden</u> an der lebenden Ratte sind Ihrer Meinung nach besonders anspruchsvoll für die/den Durchzuführende(n)?

Bitte nennen Sie 3 Methoden aus der <u>1.und 2. Frage</u>. Bitte geben Sie diese <u>vollständig</u> an.

1. ____

2. ____

3. ____

4. Bitte wählen Sie aus der unten stehenden Liste <u>die 5 Methoden</u> an der lebenden Ratte, für die Sie ein vorbereitendes Training an Simulatoren für besonders sinnvoll einschätzen.

Sie können bis zu <u>5 Methoden</u> auswählen.

☐ Handling - Transfer von Käfig zu Käfig
☐ Fixieren (mit verschiedenen Griffen)
☐ Ohrlochmarkierung
☐ Applikation per os ohne Sonde
☐ Applikation per os mit Sonde
☐ Applikation subkutan Nackenhautfalte
☐ Applikation subkutan seitliche Bauchhautfalte (Flanke)
☐ Applikation intramuskulär
☐ Applikation intraperitoneal
☐ Applikation intravenös in die dorsale Penisvene
☐ Applikation intravenös in die laterale Schwanzvene
☐ Blutentnahme aus der Vena sublingualis
☐ Blutentnahme aus retrobulbärem Venenplexus
☐ Blutentnahme aus der Vena saphena
☐ Blutentnahme aus der lateralen Schwanzvene
☐ Blutentnahme kardial

5. Würden Sie sich ein Simulator-Training auch für <u>andere</u> Methoden an der Ratte wünschen?

Falls ja, beschreiben Sie uns bitte, welche Methode(n) Ihnen wichtig wäre(n).

Ihr persönliches Feedback zur Rat Simulator D

6. Wie realitätsnah sind folgende Merkmale an der Rat Simulator D im Vergleich zu einer echten Ratte?

**Merkmale**	**Sehr realistisch** 1	**Ziemlich realistisch** 2	**Eher realistisch** 3	**Eher unrealistisch** 4	**Ziemlich unrealistisch** 5	**Sehr unrealistisch** 6
Gesamterscheinung	o	o	o	o	o	o
Haptik	o	o	o	o	o	o
Verschieblichkeit der Haut im Nacken	o	o	o	o	o	o
Verschieblichkeit der Haut an der seitlichen Bauchwand (Flanke)	o	o	o	o	o	o
Konsistenz der Hautoberfläche	o	o	o	o	o	o
Körpergröße	o	o	o	o	o	o
Gewicht	o	o	o	o	o	o
Körperform	o	o	o	o	o	o
Proportionen	o	o	o	o	o	o

7. Wie realitätsnah war für Sie das Training an der Rat Simulator D im Vergleich zu einer echten Ratte?

**Versuchstierkundliche Methoden**	**Sehr realistisch** 1	**Ziemlich realistisch** 2	**Eher realistisch** 3	**Eher unrealistisch** 4	**Ziemlich unrealistisch** 5	**Sehr unrealistisch** 6	**Methode nicht trainiert.**
Handling - Transfer von Käfig zu Käfig	o	o	o	o	o	o	o
Fixieren mit Nackengriff	o	o	o	o	o	o	o
Fixieren mit oberem Schultergriff	o	o	o	o	o	o	o
Fixieren mit mittlerem Schultergriff	o	o	o	o	o	o	o
Fixieren mit unterem Schultergriff	o	o	o	o	o	o	o
Ohrlochmarkierung	o	o	o	o	o	o	o
Applikation intravenös in die laterale Schwanzvene	o	o	o	o	o	o	o
Blutentnahme aus der lateralen Schwanzvene	o	o	o	o	o	o	o
Sonstiges: ________________________________	o	o	o	o	o	o	o
________________________________	o	o	o	o	o	o	o

8. Wie gut treffen folgende Aussagen über das Training an der Rat Simulator D zu?

	**Trifft voll und ganz zu** 1	**Trifft weitgehend zu** 2	**Trifft eher zu** 3	**Trifft eher nicht zu** 4	**Trifft weitgehend nicht zu** 5	**Trifft ganz und gar nicht zu** 6
Ich habe mich beim **Handling und Fixieren** an der lebenden Ratte sicherer gefühlt.	o	o	o	o	o	o
Ich habe mich bei den **versuchstierkundlichen Methoden** an der lebenden Ratte sicherer gefühlt.	o	o	o	o	o	o

9. Wie gut hat Sie die Rat Simulator D auf das gesamte Kurstraining an der lebenden Ratte vorbereitet?					
Sehr gut	Ziemlich gut	Eher gut	Eher schlecht	Ziemlich schlecht	Sehr schlecht
1	2	3	4	5	6
o	o	o	o	o	o

10. Wie gut hat Sie die Rat Simulator D auf die folgenden Methoden im Kurstraining an der lebenden Ratte vorbereitet?

Versuchstierkundliche Methoden	Sehr gut 1	Ziemlich gut 2	Eher gut 3	Eher schlecht 4	Ziemlich schlecht 5	Sehr schlecht 6	Methode nicht trainiert.
Handling - Transfer von Käfig zu Käfig	o	o	o	o	o	o	o
Fixieren mit Nackengriff	o	o	o	o	o	o	o
Fixieren mit oberem Schultergriff	o	o	o	o	o	o	o
Fixieren mit mittlerem Schultergriff	o	o	o	o	o	o	o
Fixieren mit unterem Schultergriff	o	o	o	o	o	o	o
Ohrlochmarkierung	o	o	o	o	o	o	o
Applikation intravenös in die laterale Schwanzvene	o	o	o	o	o	o	o
Blutentnahme aus der lateralen Schwanzvene	o	o	o	o	o	o	o
Sonstiges: ___________________________________	o	o	o	o	o	o	o
___________________________________	o	o	o	o	o	o	o

11. Was hat Ihnen an der Rat Simulator D besonders gut gefallen?

12. Was hat Ihnen an der Rat Simulator D nicht gefallen?

13. Was würden Sie an der Rat Simulator D gerne verbessern?

14. Mit welchen Tierarten werden Sie nach bestandenem Kurs in den nächsten 6 Monaten voraussichtlich versuchstierkundlich arbeiten?
Mehrfachantworten sind bei den Tierarten möglich.

- ☐ Ratte
- ☐ Maus
- ☐ Kaninchen
- ☐ Meerschwein
- ☐ Hamster
- ☐ Schwein
- ☐ Schaf
- ☐ Ziege
- ☐ Rind
- ☐ Pferd
- ☐ Hund
- ☐ Katze
- ☐ Sonstiges ___________________________
- ☐ Keine
- ☐ Kann ich nicht beurteilen.

15. Wie alt sind Sie?

________ Jahre

16. Welches Geschlecht haben Sie?

- ☐ Männlich
- ☐ Weiblich
- ☐ Divers
- ☐ Keine Angabe

17. Bitte kreuzen Sie Ihren höchsten abgeschlossenen Bildungsabschluss an.
Nur eine Antwort ist möglich

- ☐ (Noch) kein beruflicher Abschluss
- ☐ Abgeschlossene berufliche-/ betriebliche-/ schulische Ausbildung
- ☐ Abgeschlossene Ausbildung an Fachschule, Meister-, Technikerschule, Berufs- oder Fachakademie
- ☐ Fachhochschulabschluss
- ☐ Bachelorabschluss
- ☐ Master-, Magister-, Diplom- oder Staatsexamensabschluss
- ☐ Promotion
- ☐ Sonstiges: ____

18. Bitte vervollständigen Sie den Satz:
Nur eine Antwort ist möglich.

Ich besuche den Kurs als …

- ☐ …wissenschaftlich Beschäftigte/r.
- ☐ …wissenschaftlich-technische/r Mitarbeiter/in.
- ☐ …Auszubildende/r.
- ☐ …Studierende/r.
- ☐ …Sonstiges: ____.

19. Aus welcher Fachrichtung kommen Sie?
Nur eine Antwort ist möglich.

- ☐ Humanmedizin
- ☐ Veterinärmedizin
- ☐ Zahnmedizin
- ☐ Pharmazie
- ☐ Biologie
- ☐ Chemie
- ☐ Physik
- ☐ Biotechnologie
- ☐ Ernährungswissenschaft
- ☐ Sonstiges: ____

20. Hatten Sie vor dem Kurs bereits Erfahrung …
Nur eine Antwort ist möglich.

…im Handling von Ratten?

- ☐ Ja, viel.
- ☐ Ja, etwas.
- ☐ Nein, gar keine.

21. Hatten Sie vor dem Kurs bereits Erfahrung …
Nur eine Antwort ist möglich.

…im Handling von Mäusen?

- ☐ Ja, viel.
- ☐ Ja, etwas.
- ☐ Nein, gar keine.

22. Haben Sie vor dem Kurs schon einmal mit Simulatoren gearbeitet?
Mehrere Antworten für Simulatoren sind möglich. Bitte beschreiben Sie das Modell kurz.

- ☐ Ja, …
  - ☐ …mit Ratten-Simulatoren: Modell: ____
  - ☐ …mit Maus-Simulatoren: Modell: ____
  - ☐ …mit sonstigen Simulatoren: Modell: ____
- ☐ Nein.

Haben Sie weitere Anmerkungen, Lob oder Kritik? Wir freuen uns über Ihr Feedback!

Herzlichen Dank für Ihre Teilnahme!

Das SimulRATor-Team

web: www.simulator.de mail: kontakt@simulrator.de

DEUTSCHES ZENTRUM ZUM SCHUTZ VON VERSUCHSTIEREN

Freie Universität Berlin

Herzlich willkommen bei „SimulRATor“

Wir laden Sie ein, an unserer Umfrage über versuchstierkundliche Simulatoren teilzunehmen.

Wir sind ein Team aus Wissenschaftler/innen der Institute für Veterinär-Anatomie, für Tierschutz, Tierverhalten und Versuchstierkunde und für Veterinär-Epidemiologie und Biometrie am Fachbereich Veterinärmedizin der Freien Universität Berlin **und evaluieren in einem Forschungsprojekt alle derzeit kommerziell erhältlichen Ratten- und Maussimulatoren** in versuchstierkundlichen Kursen.

Da Sie als Kursteilnehmer/in am besten bewerten können, wie gut man an einem Simulator trainieren und sich auf die praktischen Übungen am Tier im Kurs vorbereiten kann, bitten wir Sie um **Ihr persönliches Feedback über die Stärken und Schwächen der Simulatoren** mit denen Sie im Kurs trainiert haben.

Danke für Ihre Unterstützung!

Das SimulRATor-Team

Weitere Informationen finden Sie auf www.simulrator.de oder schreiben Sie uns auf kontakt@simulrator.de.

Teil 1 von 2 Rat Simulator E

ID: ____________________

➔ **Bitte merken Sie sich Ihre ID für Teil 2. Danke!**

Hinweis zum Datenschutz: Ihre Teilnahme an der Umfrage ist freiwillig. Die Umfragedaten werden anonymisiert gespeichert und verwaltet. Keinesfalls werden Daten an Dritte weitergegeben. Wir erheben keine personenbezogenen Daten von Ihnen. Es sind keine Rückschlüsse auf Ihre Teilnahme möglich. Nur Projektbeteiligte haben Zugriff auf die von Ihnen innerhalb des Fragebogens gemachten Angaben und sind zur Verschwiegenheit verpflichtet. Die gemachten Angaben werden ausschließlich für Forschungszwecke genutzt. Sie können die Umfrage jederzeit ohne Angabe von Gründen abbrechen.

1. Wie gut gelangen Ihnen die versuchstierkundlichen Methoden an der Rat Simulator E?

Versuchstierkundliche Methoden	Sehr schlecht 1	Ziemlich schlecht 2	Eher schlecht 3	Eher gut 4	Ziemlich gut 5	Sehr gut 6	Methode nicht trainiert.
Handling - Transfer von Käfig zu Käfig	o	o	o	o	o	o	o
Fixieren mit Nackengriff	o	o	o	o	o	o	o
Fixieren mit oberem Schultergriff	o	o	o	o	o	o	o
Fixieren mit mittlerem Schultergriff	o	o	o	o	o	o	o
Fixieren mit unterem Schultergriff	o	o	o	o	o	o	o
Applikation per os mit Sonde	o	o	o	o	o	o	o
Applikation subkutan Nackenhautfalte	o	o	o	o	o	o	o
Applikation subkutan seitliche Bauchhautfalte (Flanke)	o	o	o	o	o	o	o
Applikation intramuskulär	o	o	o	o	o	o	o
Applikation intravenös in die laterale Schwanzvene	o	o	o	o	o	o	o
Blutentnahme aus der lateralen Schwanzvene	o	o	o	o	o	o	o
Sonstiges: ___________________________________	o	o	o	o	o	o	o
___________________________________	o	o	o	o	o	o	o

2. Wie gut treffen folgenden Aussagen über das Training an der Rat Simulator E zu?

	Trifft voll und ganz zu 1	Trifft weitgehend zu 2	Trifft eher zu 3	Trifft eher nicht zu 4	Trifft weitgehend nicht zu 5	Trifft ganz und gar nicht zu 6
Die Anzahl an Teilnehmer/innen pro Rat Simulator E war angemessen.	o	o	o	o	o	o
Die Trainingsdauer an der Rat Simulator E war ausreichend.	o	o	o	o	o	o
Die Methoden lassen sich an der Rat Simulator E zufriedenstellend trainieren.	o	o	o	o	o	o
Durch das Training an der Rat Simulator E fühle ich mich für das Kurstraining an der lebenden Ratte besser vorbereitet.	o	o	o	o	o	o

3. Falls es zu materialbedingten Schwierigkeiten bei der Verwendung der Rat Simulator E kam, bitte nennen Sie uns diese.

Versuchstierkundliche Methode: ____

Beschreibung der Komplikation: ____

____

____

Versuchstierkundliche Methode: ____

Beschreibung der Komplikation: ____

____

____

Haben Sie weitere Anmerkungen, Lob oder Kritik? Wir freuen uns über Ihr Feedback!

Herzlichen Dank für Ihre Teilnahme!

Das SimulRATor-Team kontakt@simulrator.de

DEUTSCHES ZENTRUM ZUM SCHUTZ VON VERSUCHSTIEREN

Freie Universität Berlin

Herzlich willkommen bei „SimulRATor"

Wir laden Sie ein, an unserer Umfrage über versuchstierkundliche Simulatoren teilzunehmen.

Wir sind ein Team aus Wissenschaftler/innen der Institute für Veterinär-Anatomie, für Tierschutz, Tierverhalten und Versuchstierkunde und für Veterinär-Epidemiologie und Biometrie am Fachbereich Veterinärmedizin der Freien Universität Berlin **und evaluieren in einem Forschungsprojekt alle derzeit kommerziell erhältlichen Ratten- und Maussimulatoren** in versuchstierkundlichen Kursen.

Da Sie als Kursteilnehmer/in am besten bewerten können, wie gut man an einem Simulator trainieren und sich auf die praktischen Übungen am Tier im Kurs vorbereiten kann, bitten wir Sie um **Ihr persönliches Feedback über die Stärken und Schwächen der Simulatoren** mit denen Sie im Kurs trainiert haben.

Danke für Ihre Unterstützung!

Das SimulRATor-Team

Weitere Informationen finden Sie auf www.simulrator.de oder schreiben Sie uns auf kontakt@simulrator.de.

Teil 2 von 2 Rat Simulator E

ID: ________________

Hinweis zum Datenschutz: Ihre Teilnahme an der Umfrage ist freiwillig. Die Umfragedaten werden anonymisiert gespeichert und verwaltet. Keinesfalls werden Daten an Dritte weitergegeben. Wir erheben keine personenbezogenen Daten von Ihnen. Es sind keine Rückschlüsse auf Ihre Teilnahme möglich. Nur Projektbeteiligte haben Zugriff auf die von Ihnen innerhalb des Fragebogens gemachten Angaben und sind zur Verschwiegenheit verpflichtet. Die gemachten Angaben werden ausschließlich für Forschungszwecke genutzt. Sie können die Umfrage jederzeit ohne Angabe von Gründen abbrechen.

1. Wie gut gelangen Ihnen Handling, Fixieren und Markieren an der lebenden Ratte?

Versuchstierkundliche Methoden	Sehr gut 1	Ziemlich gut 2	Eher gut 3	Eher schlecht 4	Ziemlich schlecht 5	Sehr schlecht 6	Methode nicht trainiert.
Handling - Transfer von Käfig zu Käfig	o	o	o	o	o	o	o
Fixieren mit Nackengriff	o	o	o	o	o	o	o
Fixieren mit oberem Schultergriff	o	o	o	o	o	o	o
Fixieren mit mittlerem Schultergriff	o	o	o	o	o	o	o
Fixieren mit unterem Schultergriff	o	o	o	o	o	o	o
Ohrlochmarkierung	o	o	o	o	o	o	o
Sonstiges: ___________________________________	o	o	o	o	o	o	o
___________________________________	o	o	o	o	o	o	o

2. Wie gut gelangen Ihnen die folgenden versuchstierkundlichen Methoden an der lebenden Ratte?

Bitte kreuzen Sie zusätzlich das Kästchen der letzten Spalte an, wenn die Ratte für die Methode narkotisiert war.

Versuchstierkundliche Methoden	Sehr gut 1	Ziemlich gut 2	Eher gut 3	Eher schlecht 4	Ziemlich schlecht 5	Sehr schlecht 6	Nicht trainiert.	Ratte in Narkose.
Applikation per os ohne Sonde	o	o	o	o	o	o	o	☐
Applikation per os mit Sonde	o	o	o	o	o	o	o	☐
Applikation subkutan Nackenhautfalte	o	o	o	o	o	o	o	☐
Applikation subkutan seitliche Bauchhautfalte (Flanke)	o	o	o	o	o	o	o	☐
Applikation intramuskulär	o	o	o	o	o	o	o	☐
Applikation intraperitoneal	o	o	o	o	o	o	o	☐
Applikation intravenös in die dorsale Penisvene	o	o	o	o	o	o	o	☐
Applikation intravenös in die laterale Schwanzvene	o	o	o	o	o	o	o	☐
Blutentnahme aus der Vena sublingualis	o	o	o	o	o	o	o	☐
Blutentnahme aus retrobulbärem Venenplexus	o	o	o	o	o	o	o	☐
Blutentnahme aus der Vena saphena	o	o	o	o	o	o	o	☐
Blutentnahme aus der lateralen Schwanzvene	o	o	o	o	o	o	o	☐
Blutentnahme kardial	o	o	o	o	o	o	o	☐
Sonstiges: ___________________________________	o	o	o	o	o	o	o	☐
___________________________________	o	o	o	o	o	o	o	☐

3. Welche 3 Methoden an der lebenden Ratte sind Ihrer Meinung nach besonders anspruchsvoll für die/den Durchzuführende(n)?

Bitte nennen Sie 3 Methoden aus der 1.und 2. Frage. Bitte geben Sie diese vollständig an.

1. ____

2. ____

3. ____

4. Bitte wählen Sie aus der unten stehenden Liste die 5 Methoden an der lebenden Ratte, für die Sie ein vorbereitendes Training an Simulatoren für besonders sinnvoll einschätzen.

Sie können bis zu 5 Methoden auswählen.

- ☐ Handling - Transfer von Käfig zu Käfig
- ☐ Fixieren (mit verschiedenen Griffen)
- ☐ Ohrlochmarkierung
- ☐ Applikation per os ohne Sonde
- ☐ Applikation per os mit Sonde
- ☐ Applikation subkutan Nackenhautfalte
- ☐ Applikation subkutan seitliche Bauchhautfalte (Flanke)
- ☐ Applikation intramuskulär
- ☐ Applikation intraperitoneal
- ☐ Applikation intravenös in die dorsale Penisvene
- ☐ Applikation intravenös in die laterale Schwanzvene
- ☐ Blutentnahme aus der Vena sublingualis
- ☐ Blutentnahme aus retrobulbärem Venenplexus
- ☐ Blutentnahme aus der Vena saphena
- ☐ Blutentnahme aus der lateralen Schwanzvene
- ☐ Blutentnahme kardial

5. Würden Sie sich ein Simulator-Training auch für andere Methoden an der Ratte wünschen?

Falls ja, beschreiben Sie uns bitte, welche Methode(n) Ihnen wichtig wäre(n).

Ihr persönliches Feedback zur Rat Simulator E

6. Wie realitätsnah sind folgende Merkmale an der Rat Simulator E im Vergleich zu einer echten Ratte?

**Merkmale**	**Sehr realistisch 1**	**Ziemlich realistisch 2**	**Eher realistisch 3**	**Eher unrealistisch 4**	**Ziemlich unrealistisch 5**	**Sehr unrealistisch 6**
Gesamterscheinung	○	○	○	○	○	○
Haptik	○	○	○	○	○	○
Verschieblichkeit der Haut im Nacken	○	○	○	○	○	○
Verschieblichkeit der Haut an der seitlichen Bauchwand (Flanke)	○	○	○	○	○	○
Konsistenz der Hautoberfläche	○	○	○	○	○	○
Körpergröße	○	○	○	○	○	○
Gewicht	○	○	○	○	○	○
Körperform	○	○	○	○	○	○
Proportionen	○	○	○	○	○	○

7. Wie realitätsnah war für Sie das Training an der Rat Simulator E im Vergleich zu einer echten Ratte?

**Versuchstierkundliche Methoden**	**Sehr realistisch 1**	**Ziemlich realistisch 2**	**Eher realistisch 3**	**Eher unrealistisch 4**	**Ziemlich unrealistisch 5**	**Sehr unrealistisch 6**	**Methode nicht trainiert.**
Handling - Transfer von Käfig zu Käfig	○	○	○	○	○	○	○
Fixieren mit Nackengriff	○	○	○	○	○	○	○
Fixieren mit oberem Schultergriff	○	○	○	○	○	○	○
Fixieren mit mittlerem Schultergriff	○	○	○	○	○	○	○
Fixieren mit unterem Schultergriff	○	○	○	○	○	○	○
Applikation per os mit Sonde	○	○	○	○	○	○	○
Applikation subkutan Nackenhautfalte	○	○	○	○	○	○	○
Applikation subkutan seitliche Bauchhautfalte (Flanke)	○	○	○	○	○	○	○
Applikation intramuskulär	○	○	○	○	○	○	○
Applikation intravenös in die laterale Schwanzvene	○	○	○	○	○	○	○
Blutentnahme aus der lateralen Schwanzvene	○	○	○	○	○	○	○
Sonstiges: ________________	○	○	○	○	○	○	○
________________	○	○	○	○	○	○	○

8. Wie gut treffen folgende Aussagen über das Training an der Rat Simulator E zu?

	**Trifft voll und ganz zu 1**	**Trifft weitgehend zu 2**	**Trifft eher zu 3**	**Trifft eher nicht zu 4**	**Trifft weitgehend nicht zu 5**	**Trifft ganz und gar nicht zu 6**
Ich habe mich beim **Handling und Fixieren** an der lebenden Ratte sicherer gefühlt.	○	○	○	○	○	○
Ich habe mich bei den **versuchstierkundlichen Methoden** an der lebenden Ratte sicherer gefühlt.	○	○	○	○	○	○

9. Wie gut hat Sie die Rat Simulator E auf das gesamte Kurstraining an der lebenden Ratte vorbereitet?

Sehr gut	Ziemlich gut	Eher gut	Eher schlecht	Ziemlich schlecht	Sehr schlecht
1	2	3	4	5	6
○	○	○	○	○	○

10. Wie gut hat Sie die Rat Simulator E auf die folgenden Methoden im Kurstraining an der lebenden Ratte vorbereitet?

Versuchstierkundliche Methoden	Sehr gut 1	Ziemlich gut 2	Eher gut 3	Eher schlecht 4	Ziemlich schlecht 5	Sehr schlecht 6	Methode nicht trainiert.
Handling - Transfer von Käfig zu Käfig	○	○	○	○	○	○	○
Fixieren mit Nackengriff	○	○	○	○	○	○	○
Fixieren mit oberem Schultergriff	○	○	○	○	○	○	○
Fixieren mit mittlerem Schultergriff	○	○	○	○	○	○	○
Fixieren mit unterem Schultergriff	○	○	○	○	○	○	○
Applikation per os mit Sonde	○	○	○	○	○	○	○
Applikation subkutan Nackenhautfalte	○	○	○	○	○	○	○
Applikation subkutan seitliche Bauchhautfalte (Flanke)	○	○	○	○	○	○	○
Applikation intramuskulär	○	○	○	○	○	○	○
Applikation intravenös in die laterale Schwanzvene	○	○	○	○	○	○	○
Blutentnahme aus der lateralen Schwanzvene	○	○	○	○	○	○	○
Sonstiges: ________________________________	○	○	○	○	○	○	○
________________________________	○	○	○	○	○	○	○

11. Was hat Ihnen an der Rat Simulator E besonders gut gefallen?

12. Was hat Ihnen an der Rat Simulator E nicht gefallen?

13. Was würden Sie an der Rat Simulator E gerne verbessern?

14. Mit welchen Tierarten werden Sie nach bestandenem Kurs in den nächsten 6 Monaten voraussichtlich versuchstierkundlich arbeiten?
Mehrfachantworten sind bei den Tierarten möglich.

- ☐ Ratte
- ☐ Maus
- ☐ Kaninchen
- ☐ Meerschwein
- ☐ Hamster
- ☐ Schwein
- ☐ Schaf
- ☐ Ziege
- ☐ Rind
- ☐ Pferd
- ☐ Hund
- ☐ Katze
- ☐ Sonstiges __________________________
- ☐ Keine
- ☐ Kann ich nicht beurteilen.

15. Wie alt sind Sie?

________ Jahre

16. Welches Geschlecht haben Sie?

- ☐ Männlich
- ☐ Weiblich
- ☐ Divers
- ☐ Keine Angabe

17. Bitte kreuzen Sie Ihren <u>höchsten abgeschlossenen</u> Bildungsabschluss an.
Nur eine Antwort ist möglich

- ☐ (Noch) kein beruflicher Abschluss
- ☐ Abgeschlossene berufliche-/ betriebliche-/ schulische Ausbildung
- ☐ Abgeschlossene Ausbildung an Fachschule, Meister-, Technikerschule, Berufs- oder Fachakademie
- ☐ Fachhochschulabschluss
- ☐ Bachelorabschluss
- ☐ Master-, Magister-, Diplom- oder Staatsexamensabschluss
- ☐ Promotion
- ☐ Sonstiges: ____

18. Bitte vervollständigen Sie den Satz:
Nur eine Antwort ist möglich.

Ich besuche den Kurs als …

- ☐ …wissenschaftlich Beschäftigte/r.
- ☐ …wissenschaftlich-technische/r Mitarbeiter/in.
- ☐ …Auszubildende/r.
- ☐ …Studierende/r.
- ☐ …Sonstiges: ____.

19. Aus welcher Fachrichtung kommen Sie?
Nur eine Antwort ist möglich.

- ☐ Humanmedizin
- ☐ Veterinärmedizin
- ☐ Zahnmedizin
- ☐ Pharmazie
- ☐ Biologie
- ☐ Chemie
- ☐ Physik
- ☐ Biotechnologie
- ☐ Ernährungswissenschaft
- ☐ Sonstiges: ____

20. Hatten Sie vor dem Kurs bereits Erfahrung …
Nur eine Antwort ist möglich.

…im Handling von <u>Ratten</u>?

- ☐ Ja, viel.
- ☐ Ja, etwas.
- ☐ Nein, gar keine.

21. Hatten Sie vor dem Kurs bereits Erfahrung …
Nur eine Antwort ist möglich.

…im Handling von <u>Mäusen</u>?

- ☐ Ja, viel.
- ☐ Ja, etwas.
- ☐ Nein, gar keine.

22. Haben Sie vor dem Kurs schon einmal mit Simulatoren gearbeitet?
Mehrere Antworten für Simulatoren sind möglich. Bitte beschreiben Sie das Modell kurz.

- ☐ Ja, …
  - ☐ …mit Ratten-Simulatoren: Modell: ____
  - ☐ …mit Maus-Simulatoren: Modell: ____
  - ☐ …mit sonstigen Simulatoren: Modell: ____
- ☐ Nein.

Haben Sie weitere Anmerkungen, Lob oder Kritik? Wir freuen uns über Ihr Feedback!

Herzlichen Dank für Ihre Teilnahme!

Das SimulRATor-Team

web: www.simulator.de mail: kontakt@simulrator.de
