# Supplementary material for: Alternatives in Education—Evaluation of Rat Simulators in Laboratory Animal Training Courses from Participants’ Perspective
Source: Animals (Basel). 2021 Dec 5;11(12):3462. doi: 10.3390/ani11123462 (PMC8698197; doi:10.3390/ani11123462)
Supplement: Supplementary file 1 [file animals-11-03462-s001.zip › Table S2_Animals_28.10.21.pdf]

Table S2: Booklet designed for the simulator training workshop for the practical evaluation of currently available rat simulators.

Original PDF document of the booklets which were displayed at the simulator stations during the simulator training workshop for the practical evaluation in laboratory animal science (LAS) courses. Simulator product names were anonymized via codes (Rat simulator A -E) and illustrations were removed. For illustrations of the simulators, see Table 1.

Training with Simulators

The Simulator A

Special features

- Transparent thorax and abdomen for monitoring sucess

Practical exercises in

- Handling
- Restraint (4 grips)
- Oral gavage
- Intravenous administration via the lateral tail vein
- Blood collection from the lateral tail vein

Handling Rats – Transfer from cage to cage

Transfer from cage to cage – Do not suspend the rat by its tail!

**Material:**	**Preparation:**
➔ Gloves	➔ Put on gloves.<br>➔ Open the cage.<br>➔ Set the water bottle and cage top aside.<br>➔ Allow the rat to become aware of your presence.
**Techniques**	
➔ Grasp the rat around its shoulders:<br>○ Thumb under the shoulder on one side.<br>○ Remaining fingers on the other side. Index finger over the shoulder.<br>➔ Lift the rat up carefully with one hand and support the body with your fingertips.<br>➔ Set it down onto your other forearm/chest/ appropriate surface.	

**<u>If the animal is very active</u>**<br><br>➔ **Restrain the base of its tail between thumb and index and middle finger.**<br><br>➔ **Grasp the rat around its shoulders with the other hand.**<br><br>○ **Thumb under the shoulder on one side.**<br><br>○ **Remaining fingers on the other side. Index finger over the shoulder.**<br><br>➔ **Lift the rat up carefully, supporting the body with your fingertips.**<br><br>➔ **Set it down onto your other forearm/ chest/ appropriate surface.**	

Freie Universität Berlin

Restraining Rats I – "Scruffing"

Rats are not likely to accept this method – not commonly used for rats

**Material:**	**Preparation:**
➔ Gloves	➔ Put on gloves.<br>➔ Open the cage.<br>➔ Set the water bottle and cage top aside.<br>➔ Allow the rat to become aware of your presence.

Techniques

- ➔ Grasp the rat's base of the tail with the dominant hand.
- ➔ Gently pull backwards on a rough surface.
- ➔ Approach the scruff from the rear:
    - Apply gentle pressure to the back of the rat, over the shoulder blades.
        - Place your thumb on one side and the remaining fingers on the other side.
    - Gather up the flexible skin over the neck directly behind the ear.
    - Then gather up the flexible skin over the back with your remaining fingers.
- ➔ Control of the head is important to prevent bites.
- ➔ Rats may vocalize when restrained in this fashion.

Freie Universität Berlin

Restraining Rats II – “over the shoulder grip“

Common restraint for procedural techniques

**Material**	**Preparation**
➔ Gloves	➔ Put on gloves.<br>➔ Open the cage.<br>➔ Set the water bottle and cage top aside.<br>➔ Allow the rat to become aware of your presence.
**Techniques**	

➔ Grasp the rat’s base of the tail with the dominant hand.

➔ Gently pull backwards on a rough surface.

➔ Grasp the rat over its shoulders with your non-dominant hand approaching from the rear:

- The rat’s head is between index and middle finger, as close to the base of the fingers as possible.
- Support the chest with your ring finger, pinkie finger, and thumb.

➔ Control breathing, do not compress thorax.

➔ Stabilize the body with your other hand.

  - Grasp around the hindquarters.
  - Gently push down the hindlegs with thumb and index finger.

Restraining Rats III – "middle shoulder grip"

Common restraint for procedural techniques

Material	Preparation
➔ Gloves	➔ Put on gloves.<br>➔ Open the cage.<br>➔ Set the water bottle and cage top aside.<br>➔ Allow the rat to become aware of your presence.
**Techniques**	

➔ Grasp the rat's base of the tail with the dominant hand.

➔ Gently pull backwards on a rough surface.

➔ Grasp the rat around its shoulders with your non-dominant hand approaching from the rear:

- Thumb under the shoulder, directly under the mandible on one side.
- Remaining fingers on the other side. Index finger over the shoulder.

➔ Circle your thumb and index finger under the jaw to control the head.

➔ Remaining fingertips support the chest behind the forelegs.

➔ Control breathing. Do not compress!

➔ **Stabilize the body with your other hand.**<br><br>○ **Grasp rat around the hindquarters**<br><br>○ **Gently push down the hindlegs with thumb and index finger.**	

Restraining Rats IV – “under the shoulder grip“

Common restraint for procedural techniques

Material:	Preparation:
➔ Gloves	➔ Put on gloves.<br>➔ Open the cage.<br>➔ Set the water bottle and cage top aside.<br>➔ Allow the rat to become aware of your presence.

Techniques

➔ Grasp the base of the tail with the dominant hand.

➔ Gently pull backwards on a rough surface.

➔ Grasp the rat around its shoulders with your non-dominant hand approaching from the rear:

- Thumb on one side, under the foreleg.
- Remaining fingers on the other side under the other foreleg.

➔ Gently push up the rat's forearms with your thumb and index finger.

➔ The forearms should cross under the rat's chin, preventing it from biting.

➔ Control breathing! Do not compress!

➔ Stabilize the body with your other hand:

- Grasp around the hindquarters.
- Gently push down the hindlegs with thumb and index finger.

Oral Gavage

Enteral administration

**Material:**	**Preparation**
➔ Gloves<br>➔ Feeding tube (16-20 gauge x2-inch)<br>➔ 1ml syringe<br>➔ Media (*__only__ use air for the Simulator E!*)<br>➔ (Marker)	➔ Put on gloves.<br>Already prepared:<br>✓ Fill 1 ml syringe with medium<br>✓ CAVE: avoid air bubbles
**Techniques**	
**Measurement of the appropriate length for the feeding tube.**<br><br>➔ Restrain the animal (shoulder grip)<br>o Correct length = Distance between nose tip and last rib.<br><br>➔ Mark distance if feeding tube is longer (CAVE! Chemical composition of markers).<br><br>**Insertion of the feeding tube**<br><br>➔ Restrain the animal properly(!) in a vertical position.<br><br>➔ Place the feeding tube next to incisors and advance it along the upper palate toward the larynx.<br>(real animal: swallowing reflex)<br><br>➔ Gently straighten the animal's head.<br>o The feeding tubes slides into the esophagus by itself.<br><br>➔ Advance the feeding tube into the stomach:<br>o The needle slides down in the esophagus almost by itself.	

CAVE!

➔ Never force!

➔ In case of resistance: Stop! Pull out the needle, try again!

➔ Always observe the breathing!

➔ In case of irregular breathing:

- Stop procedure immediately.
- Set the animal back into its cage, try again after it has recovered.

Oral administration and removal of the needle.

If breathing is evenly and feeding needle is located in the stomach:

➔ Administer medium (max. 10 ml/kg) slowly and steadily.
(only use air for the Simulator E!)

➔ Remove the needle following its shape.

➔ Carefully set the animal back into its cage.

➔ Observe the animal and its breathing.

Intravenous administration via the lateral tail vein

<table>
<tr><td>
<u>Material</u>
➔ Gloves
➔ Needle (27G)
➔ 1 ml syringe
➔ Media
➔ Warm water
➔ Water bin
➔ Sharps container
➔ Restrainer/ anesthesia
➔ Gauze
➔ Alcohol/ antiseptic solution
</td><td>
<u>Preparation</u>
➔ Put on gloves.
<u>Already prepared</u>
✓ Fill 1 ml syringe with medium: avoid air bubbles.
✓ Warm up water in bin.
    o CAVE: do not heat too much!
✓ Anesthetise/ Restrain animal.
✓ Warm up the tail in the water. → veins dilate.
✓ Dry the tail.
✓ Clean the tail with an alcoholic gauze. (Use each gauze only once to wipe distally.)
</td></tr>
<tr><td><u>Techniques</u></td><td></td></tr>
<tr><td>
<u>Puncture of the lateral tail vein</u>

➔ Hold the tail between your non-dominant hand ‘s thumb and index finger.

➔ Visualize the lateral tail veins.

➔ Stabilize the vein with your thumb and index finger and use them as a guideline for puncture and injection.

➔ Start the first puncture in the distal third of the tail.

➔ Insert the needle parallel to the tail, bevel of the needle facing upwards.

➔ Penetrate 2-4 mm into the lumen of the visualized lateral tail vein.
</td><td></td></tr>
</table>

In the live animal:

- **If no blood flows, try again.**
- **If you entered the vein correctly, blood will flow back into the needle hub → intravenous administration is possible.**

Intravenous administration into the tail vein

- **Slowly inject max. 5 ml/kg weight.**
  - **There should not be any resistance!**
- **Control injection site:**
  - **If paravenous: whitish bubble appears under the skin. → New attempt proximal to current site.**
  - **If intravenous: blood proximal to the injection site gives way to the injected fluid.**

→ **Remove needle, apply pressure with a clean gauze to stop bleeding, throw needle into the sharps container.**

→ **Observe injection site and animal closely.**

Blood sampling from the lateral tail vein

Material

- ➔ Gloves
- ➔ Needle (27G)
- ➔ 1 ml syringe
- ➔ Warm water
- ➔ Water bin
- ➔ Sharps container
- ➔ Restrainer/ anesthesia
- ➔ Gauze
- ➔ Alcohol/ antiseptic solution

Preparation

- ➔ Put on gloves.

Already prepared

- ✓ Warm up water in bin,
  - CAVE: do not heat too much!
- ✓ Anesthetise/ Restrain animal
- ✓ Warm up the tail in the water. ➔ veins dilate
- ✓ Dry the tail (gauze).
- ✓ Clean the tail with an alcoholic gauze (Use each gauze only once to wipe distally.)

Techniques

Puncture of the lateral tail vein

- ➔ Hold the tail between your non-dominant hand ‘s thumb and index finger.
- ➔ Visualize the lateral tail veins.
- ➔ Stabilize the vein with your thumb and index finger and use them as a guideline for puncture and injection.
- ➔ Start the first puncture in the distal third of the tail.
- ➔ Insert the needle parallel to the tail, bevel of the needle facing upwards
- ➔ Penetrate 2-4 mm into the lumen of the visualized lateral tail vein.

Empty syringe for blood sampling!

**In the live animal:**<br><br>○ **If no blood flows, try again.**<br><br>○ **If you entered the vein correctly, blood will flow back into the needle hub. → Blood sampling is possible.**	
**<u>Blood sampling from the lateral tail vein</u>**<br><br>➔ **Blood can be sampled with a syringe or capillary tube:**<br>*(For the simulator, a syringe is recommended.)*<br><br>**CAVE: Aspiration may cause the vein to collapse!**<br>➔ **Work very slowly and carefully!**<br><br>○ **Max. volume for daily blood sampling: 0,7 ml/kg/day.**<br><br>➔ **Remove needle, apply pressure with a clean gauze to stop bleeding, throw needle into the sharps container.**<br><br>➔ **Observe injection site and animal closely.**	

Training with Simulators

The Simulator B

Special features

- Transparent thorax and abdomen for monitoring sucess

Practical exercises in

- Handling
- Restraint (4 grips)
- Oral gavage
- Intravenous administration via the lateral tail vein
- Blood collection from the lateral tail vein

Handling Rats – Transfer from cage to cage

Transfer from cage to cage – Do not suspend the rat by its tail!

**Material:**	**Preparation:**
➔ Gloves	➔ Put on gloves.<br>➔ Open the cage.<br>➔ Set the water bottle and cage top aside.<br>➔ Allow the rat to become aware of your presence.
**Techniques**	
➔ Grasp the rat around its shoulders:<br>○ Thumb under the shoulder on one side.<br>○ Remaining fingers on the other side. Index finger over the shoulder.<br>➔ Lift the rat up carefully with one hand and support the body with your fingertips.<br>➔ Set it down onto your other forearm/ chest/appropriate surface.	

<u>If the animal is very active</u>

- ➔ **Restrain the base of its tail between thumb and index and middle finger.**
- ➔ **Grasp the rat around its shoulders with the other hand.**
  - **Thumb under the shoulder on one side.**
  - **Remaining fingers on the other side. Index finger over the shoulder.**
- ➔ **Lift the rat up carefully, supporting the body with your fingertips.**
- ➔ **Set it down onto your other forearm/ chest/ appropriate surface.**

Restraining Rats I – "Scruffing"

Rats are not likely to accept this method – not commonly used for rats

**Material:**	**Preparation:**
➔ Gloves	➔ Put on gloves.<br>➔ Open the cage.<br>➔ Set the water bottle and cage top aside.<br>➔ Allow the rat to become aware of your presence.

Techniques

- ➔ Grasp the rat's base of the tail with the dominant hand.
- ➔ Gently pull backwards on a rough surface.
- ➔ Approach the scruff from the rear:
    - Apply gentle pressure to the back of the rat, over the shoulder blades.
        - Place your thumb on one side and the remaining fingers on the other side.
    - Gather up the flexible skin over the <u>neck</u> directly behind the ear.
    - Then gather up the flexible skin over the <u>back</u> with your remaining fingers.
- ➔ Control of the head is important to prevent bites.
- ➔ Rats may vocalize when restrained in this fashion.

Restraining Rats II – "over the shoulder grip"

Common restraint for procedural techniques

Material	Preparation
→ Gloves	→ Put on gloves.<br>→ Open the cage.<br>→ Set the water bottle and cage top aside.<br>→ Allow the rat to become aware of your presence.

Techniques

→ Grasp the rat's base of the tail with the dominant hand.

→ Gently pull backwards on a rough surface.

→ Grasp the rat over its shoulders with your non-dominant hand approaching from the rear:

- The rat's head is between index and middle finger, as close to the base of the fingers as possible.
- Support the chest with your ring finger, pinkie finger, and thumb.

→ Control breathing, do not compress thorax.

→ Stabilize the body with your other hand.

- Grasp around the hindquarters.
- Gently push down the hindlegs with thumb and index finger.

Freie Universität Berlin

Restraining Rats III – "middle shoulder grip"

Common restraint for procedural techniques

**Material**	**Preparation**
➔ Gloves	➔ Put on gloves.<br>➔ Open the cage.<br>➔ Set the water bottle and cage top aside.<br>➔ Allow the rat to become aware of your presence.
**Techniques**	

➔ Grasp the rat's base of the tail with the dominant hand.

➔ Gently pull backwards on a rough surface.

➔ Grasp the rat around its shoulders with your non-dominant hand approaching from the rear:

- Thumb under the shoulder, directly under the mandible on one side.
- Remaining fingers on the other side. Index finger over the shoulder.

➔ Circle your thumb and index finger under the jaw to control the head.

➔ Remaining fingertips support the chest behind the forelegs.

➔ Control breathing. Do not compress!

➔ **Stabilize the body with your other hand.**<br><br>○ **Grasp rat around the hindquarters**<br><br>○ **Gently push down the hindlegs with thumb and index finger.**	

Freie Universität Berlin

Restraining Rats IV – “under the shoulder grip“

Common restraint for procedural techniques

Material:	Preparation:
➔ Gloves	➔ Put on gloves.<br>➔ Open the cage.<br>➔ Set the water bottle and cage top aside.<br>➔ Allow the rat to become aware of your presence.

Techniques

➔ Grasp the base of the tail with the dominant hand.

➔ Gently pull backwards on a rough surface.

➔ Grasp the rat around its shoulders with your non-dominant hand approaching from the rear:

- Thumb on one side, under the foreleg.
- Remaining fingers on the other side under the other foreleg.

➔ Gently push up the rat's forearms with your thumb and index finger.

➔ The forearms should cross under the rat's chin, preventing it from biting.

➔ Control breathing! Do not compress!

➔ Stabilize the body with your other hand:

- Grasp around the hindquarters.
- Gently push down the hindlegs with thumb and index finger.

Oral Gavage

Enteral administration

Material:	Preparation
➔ Gloves<br>➔ Feeding tube (16-20 gauge x2-inch)<br>➔ 1 ml syringe<br>➔ Media (*only use air for the Simulator E!*)<br>➔ (Marker)	➔ Put on gloves.<br>Already prepared:<br>✓ Fill 1 ml syringe with medium<br>✓ CAVE: avoid air bubbles
**Techniques**	
**Measurement of the appropriate length for the feeding tube.**<br><br>➔ Restrain the animal (shoulder grip)<br>o Correct length = Distance between nose tip and last rib.<br><br>➔ Mark distance if feeding tube is longer (CAVE! Chemical composition of markers).<br><br>**Insertion of the feeding tube**<br><br>➔ Restrain the animal properly(!) in a vertical position.<br><br>➔ Place the feeding tube next to incisors and advance it along the upper palate toward the larynx.<br>(real animal: swallowing reflex)<br><br>➔ Gently straighten the animal's head.<br>o The feeding tubes slides into the esophagus by itself.<br><br>➔ Advance the feeding tube into the stomach:<br>o The needle slides down in the esophagus almost by itself.	

CAVE!

➔ **Never force!**

➔ **In case of resistance: Stop! Pull out the needle, try again!**

➔ **Always observe the breathing!**

➔ **In case of irregular breathing:**

- **Stop procedure immediately.**
- **Set the animal back into its cage, try again after it has recovered.**

Oral administration and removal of the needle.

If breathing is evenly and feeding needle is located in the stomach:

➔ **Administer medium (max. 10 ml/kg) slowly and steadily.**
(only use air for the Simulator E!)

➔ **Remove the needle following its shape.**

➔ **Carefully set the animal back into its cage.**

➔ **Observe the animal and its breathing.**

<u>Intravenous administration via the lateral tail vein</u>

**<u>Material</u>**	**<u>Preparation</u>**
➔ Gloves<br>➔ Needle (27G)<br>➔ 1 ml syringe<br>➔ Media<br>➔ Warm water<br>➔ Water bin<br>➔ Sharps container<br>➔ Restrainer/ anesthesia<br>➔ Gauze<br>➔ Alcohol/ antiseptic solution	➔ Put on gloves.<br><u>Already prepared</u><br>✓ Fill 1 ml syringe with medium: avoid air bubbles.<br>✓ Warm up water in bin.<br>○ CAVE: do not heat too much!<br>✓ Anesthetise/ Restrain animal.<br>✓ Warm up the tail in the water. → veins dilate.<br>✓ Dry the tail.<br>✓ Clean the tail with an alcoholic gauze. (Use each gauze only once to wipe distally.)
**<u>Techniques</u>**	
**<u>Puncture of the lateral tail vein</u>**<br><br>➔ Hold the tail between your non-dominant hand 's thumb and index finger.<br><br>➔ Visualize the lateral tail veins.<br><br>➔ Stabilize the vein with your thumb and index finger and use them as a guideline for puncture and injection.<br><br>➔ Start the first puncture in the distal third of the tail.<br><br>➔ Insert the needle parallel to the tail, bevel of the needle facing upwards.<br><br>➔ Penetrate 2-4 mm into the lumen of the visualized lateral tail vein.	

**In the live animal:**<br><br>○ **If no blood flows, try again.**<br><br>○ **If you entered the vein correctly, blood will flow back into the needle hub → intravenous administration is possible.**	
**<u>Intravenous administration into the tail vein</u>**<br><br>○ **Slowly inject max. 5 ml/kg weight.**<br><br>○ **There should not be any resistance!**<br><br>○ **Control injection site:**<br><br>○ **If paravenous: whitish bubble appears under the skin. → New attempt proximal to current site.**<br><br>○ **If intravenous: blood proximal to the injection site gives way to the injected fluid.**<br><br>➔ **Remove needle, apply pressure with a clean gauze to stop bleeding, throw needle into the sharps container.**<br><br>➔ **Observe injection site and animal closely.**	

Blood sampling from the lateral tail vein

Material	Preparation
➔ Gloves<br>➔ Needle (27G)<br>➔ 1 ml syringe<br>➔ Warm water<br>➔ Water bin<br>➔ Sharps container<br>➔ Restrainer/ anesthesia<br>➔ Gauze<br>➔ Alcohol/Antiseptic solution	➔ Put on gloves.<br>Already prepared<br>✓ Warm up water in bin,<br>○ CAVE: do not heat too much!<br>✓ Anesthetise/ Restrain animal<br>✓ Warm up the tail in the water. ➔ veins dilate<br>✓ Dry the tail (gauze).<br>✓ Clean the tail with an alcoholic gauze (Use each gauze only once to wipe distally.)
**Techniques**	
**Puncture of the lateral tail vein**<br><br>➔ Hold the tail between your non-dominant hand 's thumb and index finger.<br><br>➔ Visualize the lateral tail veins.<br><br>➔ Stabilize the vein with your thumb and index finger and use them as a guideline for puncture and injection.<br><br>➔ Start the first puncture in the distal third of the tail.<br><br>➔ Insert the needle parallel to the tail, bevel of the needle facing upwards<br><br>➔ Penetrate 2-4 mm into the lumen of the visualized lateral tail vein.	

**In the live animal:**<br><br>- **If no blood flows, try again.**<br>- **If you entered the vein correctly, blood will flow back into the needle hub. → Blood sampling is possible.**	
**<u>Blood sampling from the lateral tail vein</u>**<br><br>➔ **Blood can be sampled with a syringe or capillary tube:**<br>*(For the simulator, a syringe is recommended.)*<br><br>**CAVE: Aspiration may cause the vein to collapse!**<br>➔ **Work very slowly and carefully!**<br><br>- **Max. volume for daily blood sampling: 0,7 ml/kg/day.**<br><br>➔ **Remove needle, apply pressure with a clean gauze to stop bleeding, throw needle into the sharps container.**<br><br>➔ **Observe injection site and animal closely.**	

Training with Simulators

The Simulator C

Practical exercises in

- Handling
- Restraint (4 grips)
- Intravenous administration via the lateral tail vein
- Blood collection from the lateral tail vein
- Blood collection from the saphenous vein
- Blood collection from the heart

Handling Rats – Transfer from cage to cage

Transfer from cage to cage – Do not suspend the rat by its tail!

**Material:**	**Preparation:**
➔ Gloves	➔ Put on gloves.<br>➔ Open the cage.<br>➔ Set the water bottle and cage top aside.<br>➔ Allow the rat to become aware of your presence.
**Techniques**	
➔ Grasp the rat around its shoulders:<br>○ Thumb under the shoulder on one side.<br>○ Remaining fingers on the other side. Index finger over the shoulder.<br>➔ Lift the rat up carefully with one hand and support the body with your fingertips.<br>➔ Set it down onto your other forearm/chest/ appropriate surface.	

**<u>If the animal is very active</u>**<br><br>➔ **Restrain the base of its tail between thumb and index and middle finger.**<br><br>➔ **Grasp the rat around its shoulders with the other hand.**<br><br>○ **Thumb under the shoulder on one side.**<br><br>○ **Remaining fingers on the other side. Index finger over the shoulder.**<br><br>➔ **Lift the rat up carefully, supporting the body with your fingertips.**<br><br>➔ **Set it down onto your other forearm/ chest/ appropriate surface.**	

Restraining Rats I – "Scruffing"

Rats are not likely to accept this method – not commonly used for rats

**Material:**	**Preparation:**
➔ Gloves	➔ Put on gloves.<br>➔ Open the cage.<br>➔ Set the water bottle and cage top aside.<br>➔ Allow the rat to become aware of your presence.

Techniques

- ➔ Grasp the rat's base of the tail with the dominant hand.
- ➔ Gently pull backwards on a rough surface.
- ➔ Approach the scruff from the rear:
  - o Apply gentle pressure to the back of the rat, over the shoulder blades.
    - ▪ Place your thumb on one side and the remaining fingers on the other side.
  - o Gather up the flexible skin over the neck directly behind the ear.
  - o Then gather up the flexible skin over the back with your remaining fingers.
- ➔ Control of the head is important to prevent bites.
- ➔ Rats may vocalize when restrained in this fashion.

Freie Universität Berlin

Restraining Rats II – "over the shoulder grip"

Common restraint for procedural techniques

Material	Preparation
➔ Gloves	➔ Put on gloves.<br>➔ Open the cage.<br>➔ Set the water bottle and cage top aside.<br>➔ Allow the rat to become aware of your presence.

Techniques

➔ Grasp the rat's base of the tail with the dominant hand.

➔ Gently pull backwards on a rough surface.

➔ Grasp the rat over its shoulders with your non-dominant hand approaching from the rear:

- The rat's head is between index and middle finger, as close to the base of the fingers as possible.
- Support the chest with your ring finger, pinkie finger, and thumb.

➔ Control breathing, do not compress thorax.

➔ Stabilize the body with your other hand.

- Grasp around the hindquarters.
- Gently push down the hindlegs with thumb and index finger.

Freie Universität Berlin

Restraining Rats III – "middle shoulder grip"

Common restraint for procedural techniques

Material	Preparation
➔ Gloves	➔ Put on gloves.<br>➔ Open the cage.<br>➔ Set the water bottle and cage top aside.<br>➔ Allow the rat to become aware of your presence.
**Techniques**	

➔ Grasp the rat's base of the tail with the dominant hand.

➔ Gently pull backwards on a rough surface.

➔ Grasp the rat around its shoulders with your non-dominant hand approaching from the rear:

- Thumb under the shoulder, directly under the mandible on one side.
- Remaining fingers on the other side. Index finger over the shoulder.

➔ Circle your thumb and index finger under the jaw to control the head.

➔ Remaining fingertips support the chest behind the forelegs.

➔ Control breathing. Do not compress!

➔ **Stabilize the body with your other hand.**<br><br>○ **Grasp rat around the hindquarters**<br><br>○ **Gently push down the hindlegs with thumb and index finger.**	

Restraining Rats IV – "under the shoulder grip"

Common restraint for procedural techniques

Material:	Preparation:
➔ Gloves	➔ Put on gloves.<br>➔ Open the cage.<br>➔ Set the water bottle and cage top aside.<br>➔ Allow the rat to become aware of your presence.

Techniques

- ➔ Grasp the base of the tail with the dominant hand.
- ➔ Gently pull backwards on a rough surface.
- ➔ Grasp the rat around its shoulders with your non-dominant hand approaching from the rear:
  - Thumb on one side, under the foreleg.
  - Remaining fingers on the other side under the other foreleg.
- ➔ Gently push up the rat's forearms with your thumb and index finger.
- ➔ The forearms should cross under the rat's chin, preventing it from biting.
- ➔ Control breathing! Do not compress!
- ➔ Stabilize the body with your other hand:
  - Grasp around the hindquarters.
  - Gently push down the hindlegs with thumb and index finger.

Freie Universität Berlin

Blood sampling from saphenous vein

for collecting small volumes

**Material:**	**Preparation**
➔ Gloves<br>➔ Electric trimmer (live animal)<br>➔ Alcoholic gauze (live animal)<br>➔ Needle (25G) / Lancet (28G)<br>➔ Heparinized (capillary) tube (live animal)<br>➔ (Anesthesia for live animal)<br>➔ Dry Gauze	✓ Put on gloves.<br>**Already done:**<br>✓ Find assisting person/Anesthetise animal.<br>✓ Shave the lateral surface of the hind leg.<br>✓ Sterilize the injection site using an alcoholic gauze.
**Techniques**	
✓ Compress the vein above the knee (not necessary for the simulator).<br><br>➔ Visualize the lateral saphenous vein.	

➔ **Puncture the vein at a 90° angle to the vein.**<br>○ **CAVE: do not pierce through!**<br>○ **Max. 3 attempts. Then, give the animal recovery time!**<br><br>➔ **If you have punctured the vein, a blood drop will become visible.**<br><br>➔ **Sample surfacing blood with a (capillary) tube:**<br>○ **Max. volume: 10 % of entire blood volume.**<br><br>✓ **Release venous compression (not necessary for the simulator).**<br><br>➔ **Wipe injection site with the dry gauze, apply momentary pressure to stop bleeding.**<br><br>➔ **Observe the injection site and animal.**	

Cardiac puncture

Final blood sampling for large volumes.
Deep anesthesia and euthanasia immediately after sampling are compulsory.

**Material:**	**Preparation**
➔ Gloves<br>➔ (Anesthesia for live animal)<br>➔ Needle (25G)<br>➔ 3 ml syringe	➔ Put on gloves.<br>➔ Anesthetise animal.<br>(Deep anesthesia ➔ Check reflexes!)
**Techniques**	
➔ Restrain the animal on its back on a solid surface (e.g. operation desk).<br><br>➔ Palpate the heart and the xyphoid process (heart is 1 cm cranial to the xyphoid process).<br><br>**Puncture from the top of the sternum**<br><br>➔ Hold the rib cage between the thumb and index finger of your non-dominant hand.<br><br>➔ Insert the needle at base of sternum bevel facing upwards in a 20-30° angle under or just left to the xyphoid process. Aim for the left shoulder.<br><br>➔ Aspirate slowly while advancing the needle.<br><br>➔ Stop needle advance when blood flows back into needle hub.<br><br>➔ Hold needle and syringe steadily, aspirate slowly until all blood is collected:<br>o Max. volume for sampling: 50 % of entire blood volume	

➔ Immediately euthanize the animal and confirm death

➔ If no blood appears:

➔

- Pull back the needle a bit and change penetration angle slightly.

- Aspirate slowly while advancing the needle.

Puncture via the left side of the chest

➔ Hold the rib cage with your index finger of your non-dominant hand.

➔ Insert the needle bevel facing upwards at a 45° angle into the intercostal space at the level of the left elbow.

➔ Aspirate slowly while advancing the needle.

➔ Stop needle advance when blood flows back into the needle hub.

➔ Hold needle and syringe steadily, aspirate slowly until all blood is collected.
- Max. volume for sampling: 50 % of entire blood volume

➔ Immediately euthanize the animal and confirm death.

➔ If no blood appears:
- Pull back the needle a bit and change penetration angle slightly.

- Aspirate slowly while advancing the needle.

Intravenous administration via the lateral tail vein

Material	Preparation
**➔ Gloves**<br>**➔ Needle (27G)**<br>**➔ 1 ml syringe**<br>**➔ Media**<br>**➔ Warm water**<br>**➔ Water bin**<br>**➔ Sharps container**<br>**➔ Restrainer/ anesthesia**<br>**➔ Gauze**<br>**➔ Alcohol/ antiseptic solution**	**➔ Put on gloves.**<br>**Already prepared**<br>✓ **Fill 1 ml syringe with medium: avoid air bubbles.**<br>✓ **Warm up water in bin.**<br>○ **CAVE: do not heat too much!**<br>✓ **Anesthetise/ Restrain animal.**<br>✓ **Warm up the tail in the water. ➔ veins dilate.**<br>✓ **Dry the tail.**<br>✓ **Clean the tail with an alcoholic gauze. (Use each gauze only once to wipe distally.)**
**Techniques**	
**Puncture of the lateral tail vein**<br><br>**➔ Hold the tail between your non-dominant hand 's thumb and index finger.**<br><br>**➔ Visualize the lateral tail veins.**<br><br>**➔ Stabilize the vein with your thumb and index finger and use them as a guideline for puncture and injection.**<br><br>**➔ Start the first puncture in the distal third of the tail.**<br><br>**➔ Insert the needle parallel to the tail, bevel of the needle facing upwards.**<br><br>**➔ Penetrate 2-4 mm into the lumen of the visualized lateral tail vein.**	

In the live animal:

- **If no blood flows, try again.**
- **If you entered the vein correctly, blood will flow back into the needle hub → intravenous administration is possible.**

Intravenous administration into the tail vein

- **Slowly inject max. 5 ml/kg weight.**
  - **There should not be any resistance!**
- **Control injection site:**
  - **If paravenous: whitish bubble appears under the skin. → New attempt proximal to current site.**
  - **If intravenous: blood proximal to the injection site gives way to the injected fluid.**

→ Remove needle, apply pressure with a clean gauze to stop bleeding, throw needle into the sharps container.

→ Observe injection site and animal closely.

Blood sampling from the lateral tail vein

**Material**	**Preparation**
→ Gloves<br>→ Needle (27G)<br>→ 1 ml syringe<br>→ Warm water<br>→ Water bin<br>→ Sharps container<br>→ Restrainer/ anesthesia<br>→ Gauze<br>→ Alcohol/Antiseptic solution	→ Put on gloves.<br>Already prepared<br>✓ Warm up water in bin,<br>○ CAVE: do not heat too much!<br>✓ Anesthetise/ Restrain animal<br>✓ Warm up the tail in the water. → veins dilate<br>✓ Dry the tail (gauze).<br>✓ Clean the tail with an alcoholic gauze (Use each gauze only once to wipe distally.)
**Techniques**	
**Puncture of the lateral tail vein**<br><br>→ Hold the tail between your non-dominant hand 's thumb and index finger.<br><br>→ Visualize the lateral tail veins.<br><br>→ Stabilize the vein with your thumb and index finger and use them as a guideline for puncture and injection.<br><br>→ Start the first puncture in the distal third of the tail.<br><br>→ Insert the needle parallel to the tail, bevel of the needle facing upwards<br><br>→ Penetrate 2-4 mm into the lumen of the visualized lateral tail vein.	Empty syringe for blood sampling!

**In the live animal:**<br><br>○ **If no blood flows, try again.**<br><br>○ **If you entered the vein correctly, blood will flow back into the needle hub. → Blood sampling is possible.**	
**<u>Blood sampling from the lateral tail vein</u>**<br><br>➔ **Blood can be sampled with a syringe or capillary tube:**<br>*(For the simulator, a syringe is recommended.)*<br><br>**CAVE: Aspiration may cause the vein to collapse!**<br>➔ **Work very slowly and carefully!**<br><br>○ **Max. volume for daily blood sampling: 0,7 ml/kg/day.**<br><br>➔ **Remove needle, apply pressure with a clean gauze to stop bleeding, throw needle into the sharps container.**<br><br>➔ **Observe injection site and animal closely.**	

Training with Simulators

The Simulator D

Practical exercises in

- Handling
- Restraint (4 grips)
- Intravenous administration via the lateral tail vein
- Blood collection from the lateral tail vein
- Ear punching

Freie Universität Berlin

Handling Rats – Transfer from cage to cage

Transfer from cage to cage – Do not suspend the rat by its tail!

**Material:**	**Preparation:**
➔ Gloves	➔ Put on gloves.<br>➔ Open the cage.<br>➔ Set the water bottle and cage top aside.<br>➔ Allow the rat to become aware of your presence.
**Techniques**	
➔ Grasp the rat around its shoulders:<br>○ Thumb under the shoulder on one side.<br>○ Remaining fingers on the other side. Index finger over the shoulder.<br>➔ Lift the rat up carefully with one hand and support the body with your fingertips.<br>➔ Set it down onto your other forearm/ chest/ appropriate surface.	

**<u>If the animal is very active</u>**<br><br>➔ **Restrain the base of its tail between thumb and index and middle finger.**<br><br>➔ **Grasp the rat around its shoulders with the other hand.**<br><br>○ **Thumb under the shoulder on one side.**<br><br>○ **Remaining fingers on the other side. Index finger over the shoulder.**<br><br>➔ **Lift the rat up carefully, supporting the body with your fingertips.**<br><br>➔ **Set it down onto your other forearm/ chest/ appropriate surface.**	

Restraining Rats I – "Scruffing"

Rats are not likely to accept this method – not commonly used for rats

Material:	Preparation:
→ Gloves	→ Put on gloves.<br>→ Open the cage.<br>→ Set the water bottle and cage top aside.<br>→ Allow the rat to become aware of your presence.

Techniques

→ Grasp the rat's base of the tail with the dominant hand.

→ Gently pull backwards on a rough surface.

→ Approach the scruff from the rear:

- Apply gentle pressure to the back of the rat, over the shoulder blades.
  - Place your thumb on one side and the remaining fingers on the other side.
- Gather up the flexible skin over the neck directly behind the ear.
- Then gather up the flexible skin over the back with your remaining fingers.

→ Control of the head is important to prevent bites.

→ Rats may vocalize when restrained in this fashion.

Freie Universität Berlin

Restraining Rats II – "over the shoulder grip"

Common restraint for procedural techniques

Material	Preparation
➔ Gloves	➔ Put on gloves.<br>➔ Open the cage.<br>➔ Set the water bottle and cage top aside.<br>➔ Allow the rat to become aware of your presence.

Techniques

➔ Grasp the rat's base of the tail with the dominant hand.

➔ Gently pull backwards on a rough surface.

➔ Grasp the rat over its shoulders with your non-dominant hand approaching from the rear:

- The rat's head is between index and middle finger, as close to the base of the fingers as possible.
- Support the chest with your ring finger, pinkie finger, and thumb.

➔ Control breathing, do not compress thorax.

➔ Stabilize the body with your other hand.

  - Grasp around the hindquarters.
  - Gently push down the hindlegs with thumb and index finger.

Restraining Rats III – "middle shoulder grip"

Common restraint for procedural techniques

Material	Preparation
➔ Gloves	➔ Put on gloves.<br>➔ Open the cage.<br>➔ Set the water bottle and cage top aside.<br>➔ Allow the rat to become aware of your presence.

Techniques

➔ Grasp the rat's base of the tail with the dominant hand.

➔ Gently pull backwards on a rough surface.

➔ Grasp the rat around its shoulders with your non-dominant hand approaching from the rear:

- Thumb under the shoulder, directly under the mandible on one side.
- Remaining fingers on the other side. Index finger over the shoulder.

➔ Circle your thumb and index finger under the jaw to control the head.

➔ Remaining fingertips support the chest behind the forelegs.

➔ Control breathing. Do not compress!

➔ **Stabilize the body with your other hand.**<br>○ **Grasp rat around the hindquarters**<br>○ **Gently push down the hindlegs with thumb and index finger.**	

Freie Universität Berlin

Restraining Rats IV – “under the shoulder grip“

Common restraint for procedural techniques

Material:	Preparation:
➔ Gloves	➔ Put on gloves.<br>➔ Open the cage.<br>➔ Set the water bottle and cage top aside.<br>➔ Allow the rat to become aware of your presence.

Techniques

➔ Grasp the base of the tail with the dominant hand.

➔ Gently pull backwards on a rough surface.

➔ Grasp the rat around its shoulders with your non-dominant hand approaching from the rear:

- Thumb on one side, under the foreleg.
- Remaining fingers on the other side under the other foreleg.

➔ Gently push up the rat's forearms with your thumb and index finger.

➔ The forearms should cross under the rat's chin, preventing it from biting.

➔ Control breathing! Do not compress!

➔ Stabilize the body with your other hand:

- Grasp around the hindquarters.
- Gently push down the hindlegs with thumb and index finger.

Freie Universität Berlin

Ear punching

Permanent labelling (and genotyping)

**Material:**	**Preparation**
➔ Gloves<br>➔ Ear punch	➔ Put on gloves.<br>➔ Check functionality of ear punch.
**Techniques**	
➔ Firmly restrain the rat and its head in your non-dominant hand (e.g. over the shoulder grip).<br><br>➔ Place the flat side of ear punch on the outer auricle so that you can see the area to be punched.<br><br>○ Position the ear punch only on the outer ear and avoid vasculature.<br><br>➔ Control your position.<br><br>➔ Firmly close the ear punch whilst properly (!) restraining the animal and its head.<br><br>➔ Control punches/holes.<br><br>➔ Place animal back into its cage.<br><br>➔ Observe animal closely.	

Freie Universität Berlin

Intravenous administration via the lateral tail vein

Material

- ➔ Gloves
- ➔ Needle (27G)
- ➔ 1 ml syringe
- ➔ Media
- ➔ Warm water
- ➔ Water bin
- ➔ Sharps container
- ➔ Restrainer/ anesthesia
- ➔ Gauze
- ➔ Alcohol/ antiseptic solution

Preparation

- ➔ Put on gloves.

Already prepared

- ✓ Fill 1 ml syringe with medium: avoid air bubbles.
- ✓ Warm up water in bin.
    - CAVE: do not heat too much!
- ✓ Anesthetise/ Restrain animal.
- ✓ Warm up the tail in the water. → veins dilate.
- ✓ Dry the tail.
- ✓ Clean the tail with an alcoholic gauze. (Use each gauze only once to wipe distally.)

Techniques

Puncture of the lateral tail vein

- ➔ Hold the tail between your non-dominant hand ‘s thumb and index finger.
- ➔ Visualize the lateral tail veins.
- ➔ Stabilize the vein with your thumb and index finger and use them as a guideline for puncture and injection.
- ➔ Start the first puncture in the distal third of the tail.
- ➔ Insert the needle parallel to the tail, bevel of the needle facing upwards.
- ➔ Penetrate 2-4 mm into the lumen of the visualized lateral tail vein.

**In the live animal:**<br><br>○ **If no blood flows, try again.**<br><br>○ **If you entered the vein correctly, blood will flow back into the needle hub → intravenous administration is possible.**	
**<u>Intravenous administration into the tail vein</u>**<br><br>○ **Slowly inject max. 5 ml/kg weight.**<br><br>○ **There should not be any resistance!**<br><br>○ **Control injection site:**<br><br>○ **If paravenous: whitish bubble appears under the skin. → New attempt proximal to current site.**<br><br>○ **If intravenous: blood proximal to the injection site gives way to the injected fluid.**<br><br>→ **Remove needle, apply pressure with a clean gauze to stop bleeding, throw needle into the sharps container.**<br><br>→ **Observe injection site and animal closely.**	

Blood sampling from the lateral tail vein

Material

- ➔ Gloves
- ➔ Needle (27G)
- ➔ 1 ml syringe
- ➔ Warm water
- ➔ Water bin
- ➔ Sharps container
- ➔ Restrainer/ anesthesia
- ➔ Gauze
- ➔ Alcohol/Antiseptic solution

Preparation

- ➔ Put on gloves.

Already prepared

- ✓ Warm up water in bin,
    - CAVE: do not heat too much!
- ✓ Anesthetise/ Restrain animal
- ✓ Warm up the tail in the water. ➔ veins dilate
- ✓ Dry the tail (gauze).
- ✓ Clean the tail with an alcoholic gauze (Use each gauze only once to wipe distally.)

Techniques

Puncture of the lateral tail vein

- ➔ Hold the tail between your non-dominant hand ‘s thumb and index finger.
- ➔ Visualize the lateral tail veins.
- ➔ Stabilize the vein with your thumb and index finger and use them as a guideline for puncture and injection.
- ➔ Start the first puncture in the distal third of the tail.
- ➔ Insert the needle parallel to the tail, bevel of the needle facing upwards
- ➔ Penetrate 2-4 mm into the lumen of the visualized lateral tail vein.

**In the live animal:**<br><br>- **If no blood flows, try again.**<br>- **If you entered the vein correctly, blood will flow back into the needle hub. → Blood sampling is possible.**	
**<u>Blood sampling from the lateral tail vein</u>**<br><br>➔ **Blood can be sampled with a syringe or capillary tube:**<br>*(For the simulator, a syringe is recommended.)*<br><br>**CAVE: Aspiration may cause the vein to collapse!**<br>**➔ Work very slowly and carefully!**<br><br>- **Max. volume for daily blood sampling: 0,7 ml/kg/day.**<br><br>➔ **Remove needle, apply pressure with a clean gauze to stop bleeding, throw needle into the sharps container.**<br><br>➔ **Observe injection site and animal closely.**	

Training with Simulators

The Simulator E

Practical exercises in

- Handling
- Restraint (4 grips)
- Oral gavage
- Intravenous administration via the lateral tail vein
- Blood collection from the lateral tail vein
- Subcutaneous administration
- Intramuscular administration

Handling Rats – Transfer from cage to cage

Transfer from cage to cage – Do not suspend the rat by its tail!

**Material:**	**Preparation:**
➔ Gloves	➔ Put on gloves.<br>➔ Open the cage.<br>➔ Set the water bottle and cage top aside.<br>➔ Allow the rat to become aware of your presence.
**Techniques**	
➔ Grasp the rat around its shoulders:<br>○ Thumb under the shoulder on one side.<br>○ Remaining fingers on the other side. Index finger over the shoulder.<br>➔ Lift the rat up carefully with one hand and support the body with your fingertips.<br>➔ Set it down onto your other forearm/ chest/ appropriate surface.	

**If the animal is very active**<br><br>➔ Restrain the base of its tail between thumb and index and middle finger.<br><br>➔ Grasp the rat around its shoulders with the other hand.<br><br>○ Thumb under the shoulder on one side.<br><br>○ Remaining fingers on the other side. Index finger over the shoulder.<br><br>➔ Lift the rat up carefully, supporting the body with your fingertips.<br><br>➔ Set it down onto your other forearm/ chest/ appropriate surface.	

Restraining Rats I – "Scruffing"

Rats are not likely to accept this method – not commonly used for rats

Material:

- ➔ Gloves

Preparation:

- ➔ Put on gloves.
- ➔ Open the cage.
- ➔ Set the water bottle and cage top aside.
- ➔ Allow the rat to become aware of your presence.

Techniques

- ➔ Grasp the rat's base of the tail with the dominant hand.
- ➔ Gently pull backwards on a rough surface.
- ➔ Approach the scruff from the rear:
  - o Apply gentle pressure to the back of the rat, over the shoulder blades.
    - ▪ Place your thumb on one side and the remaining fingers on the other side.
  - o Gather up the flexible skin over the <u>neck</u> directly behind the ear.
  - o Then gather up the flexible skin over the <u>back</u> with your remaining fingers.
- ➔ Control of the head is important to prevent bites.
- ➔ Rats may vocalize when restrained in this fashion.

Restraining Rats II – "over the shoulder grip"

Common restraint for procedural techniques

Material	Preparation
➔ Gloves	➔ Put on gloves.<br>➔ Open the cage.<br>➔ Set the water bottle and cage top aside.<br>➔ Allow the rat to become aware of your presence.

Techniques

➔ Grasp the rat's base of the tail with the dominant hand.

➔ Gently pull backwards on a rough surface.

➔ Grasp the rat over its shoulders with your non-dominant hand approaching from the rear:

- The rat's head is between index and middle finger, as close to the base of the fingers as possible.
- Support the chest with your ring finger, pinkie finger, and thumb.

➔ Control breathing, do not compress thorax.

➔ Stabilize the body with your other hand.

- Grasp around the hindquarters.
- Gently push down the hindlegs with thumb and index finger.

Restraining Rats III – "middle shoulder grip"

Common restraint for procedural techniques

Material	Preparation
➔ Gloves	➔ Put on gloves.<br>➔ Open the cage.<br>➔ Set the water bottle and cage top aside.<br>➔ Allow the rat to become aware of your presence.
**Techniques**	

➔ Grasp the rat's base of the tail with the dominant hand.

➔ Gently pull backwards on a rough surface.

➔ Grasp the rat around its shoulders with your non-dominant hand approaching from the rear:

- Thumb under the shoulder, directly under the mandible on one side.
- Remaining fingers on the other side. Index finger over the shoulder.

➔ Circle your thumb and index finger under the jaw to control the head.

➔ Remaining fingertips support the chest behind the forelegs.

➔ Control breathing. Do not compress!

➔ **Stabilize the body with your other hand.**<br>○ **Grasp rat around the hindquarters**<br>○ **Gently push down the hindlegs with thumb and index finger.**	

Restraining Rats IV – "under the shoulder grip"

Common restraint for procedural techniques

Material:	Preparation:
➔ Gloves	➔ Put on gloves.<br>➔ Open the cage.<br>➔ Set the water bottle and cage top aside.<br>➔ Allow the rat to become aware of your presence.

Techniques

- ➔ Grasp the base of the tail with the dominant hand.
- ➔ Gently pull backwards on a rough surface.
- ➔ Grasp the rat around its shoulders with your non-dominant hand approaching from the rear:
  - Thumb on one side, under the foreleg.
  - Remaining fingers on the other side under the other foreleg.
- ➔ Gently push up the rat's forearms with your thumb and index finger.
- ➔ The forearms should cross under the rat's chin, preventing it from biting.
- ➔ Control breathing! Do not compress!
- ➔ Stabilize the body with your other hand:
  - Grasp around the hindquarters.
  - Gently push down the hindlegs with thumb and index finger.

Oral Gavage

Enteral administration

**Material:**	**Preparation**
➔ Gloves<br>➔ Feeding tube (16-20 gauge x2-inch)<br>➔ 1 ml syringe<br>➔ Media (*only use air for the Simulator E!*)<br>➔ (Marker)	➔ Put on gloves.<br>**Already prepared:**<br>✓ Fill 1 ml syringe with medium<br>✓ CAVE: avoid air bubbles
**Techniques**	
**Measurement of the appropriate length for the feeding tube.**<br><br>➔ Restrain the animal (shoulder grip)<br>o Correct length = Distance between nose tip and last rib.<br><br>➔ Mark distance if feeding tube is longer (CAVE! Chemical composition of markers).<br><br>**Insertion of the feeding tube**<br><br>➔ Restrain the animal properly(!) in a vertical position.<br><br>➔ Place the feeding tube next to incisors and advance it along the upper palate toward the larynx.<br>(real animal: swallowing reflex)<br><br>➔ Gently straighten the animal's head.<br>o The feeding tubes slides into the esophagus by itself.<br><br>➔ Advance the feeding tube into the stomach:<br>o The needle slides down in the esophagus almost by itself.	

CAVE!

➔ **Never force!**

➔ **In case of resistance: Stop! Pull out the needle, try again!**

➔ **Always observe the breathing!**

➔ **In case of irregular breathing:**

- **Stop procedure immediately.**
- **Set the animal back into its cage, try again after it has recovered.**

Oral administration and removal of the needle.

If breathing is evenly and feeding needle is located in the stomach:

➔ **Administer medium (max. 10 ml/kg) slowly and steadily.**
(only use air for the Simulator E!)

➔ **Remove the needle following its shape.**

➔ **Carefully set the animal back into its cage.**

➔ **Observe the animal and its breathing.**

Subcutaneous Administration

Material:	Preparation:
➔ Gloves<br>➔ Needle (26G)<br>➔ 1 ml syringe<br>➔ Media (*only use air for the Simulator E!*)	➔ Put on gloves.<br>**Already done/ not needed for the Simulator E**<br>✓ Fill 1 ml syringe with medium: avoid air bubbles.
**Techniques**	
**Administration into skin fold over the neck**<br><br>➔ Restrain rat by the scruff in the neck.<br><br>➔ Insert the needle into the base of the skin fold parallel to the body axis.<br><br>➔ Gently aspirate:<br>○ Subcutaneous position: No blood will flow back into the needle hub.<br><br>➔ Inject parallel to and away from the fingers holding the skin upwards.<br><br>➔ Max. volume for application: 10 ml/ kg. !!!! Only insert air into the Simulator E!!!!<br><br>➔ Remove the needle and throw it into the sharps container.<br><br>➔ Observe injection site and the animal closely.	

Administration into skin fold over the flank	
✓ Find assisting person:<br>○ Restrain the rat by a shoulder grip.<br>○ Restrain hindquarters and hind legs with your other hand.<br><br>→ Tend the skin over the hindquarters with your non-dominant thumb, index- and middle finger.<br><br>→ Insert the needle at a 30-45° angle into the base of the skin fold<br><br>→ Gently Aspirate:<br>○ Subcutaneous position: No blood will flash back into the needle hub.<br><br>→ Inject parallel to and away from the fingers holding the skin upwards.<br><br>→ Max. volume for application: 10 ml/ kg. !!!! Only insert air into the Simulator E!!!!<br><br>→ Remove the needle and throw it into the sharps container.<br><br>→ Observe injection site and the animal closely.	

Intramuscular Administration

Only used if other routes of administration are not appropriate or potentially painful

**Material**	**Preparation**
➔ Gloves<br>➔ Needle (26G)<br>➔ 1 ml Syringe<br>➔ Media (*only use air for the Simulator E!)*	➔ Put on gloves.<br>➔ Find assisting person.<br>Already done/ not needed for the Rat Simulator E<br>✓ Fill 1 ml syringe with medium: avoid air bubbles

Techniques

➔ **Assisting person:**

- Restrain the rat by shoulder grip.
- Hold its hindquarters and one leg with the other hand.

➔ **Second person:**

- Gently extend and restrain the other hindleg.
- Stabilize thigh with three fingers (see photo).
- Insert the needle into the caudal thigh muscle.
  - CAVE: avoid the sciatic nerve and femoral vessels!

➔ **Aspirate slightly:**

- Intramuscular position: no blood will flow back into the needle hub.

➔ **Inject slowly and carefully:**

- **Max. volume for administration: 0,1 – 0,2 ml per injection.** ***(only use air for the Simulator E!)***

➔ **Remove needle and throw it in the sharps container.**

➔ **Observe injection site and animal closely.**

Intravenous administration via the lateral tail vein

Material

- ➔ Gloves
- ➔ Needle (27G)
- ➔ 1 ml syringe
- ➔ Media
- ➔ Warm water
- ➔ Water bin
- ➔ Sharps container
- ➔ Restrainer/ anesthesia
- ➔ Gauze
- ➔ Alcohol/ antiseptic solution

Preparation

- ➔ Put on gloves.

Already prepared

- ✓ Fill 1 ml syringe with medium: avoid air bubbles.
- ✓ Warm up water in bin.
    - CAVE: do not heat too much!
- ✓ Anesthetise/ Restrain animal.
- ✓ Warm up the tail in the water. → veins dilate.
- ✓ Dry the tail.
- ✓ Clean the tail with an alcoholic gauze. (Use each gauze only once to wipe distally.)

Techniques

Puncture of the lateral tail vein

- ➔ Hold the tail between your non-dominant hand 's thumb and index finger.
- ➔ Visualize the lateral tail veins.
- ➔ Stabilize the vein with your thumb and index finger and use them as a guideline for puncture and injection.
- ➔ Start the first puncture in the distal third of the tail.
- ➔ Insert the needle parallel to the tail, bevel of the needle facing upwards.
- ➔ Penetrate 2-4 mm into the lumen of the visualized lateral tail vein.

**In the live animal:**<br><br>○ **If no blood flows, try again.**<br><br>○ **If you entered the vein correctly, blood will flow back into the needle hub → intravenous administration is possible.**	
**<u>Intravenous administration into the tail vein</u>**<br><br>○ **Slowly inject max. 5 ml/kg weight.**<br><br>○ **There should not be any resistance!**<br><br>○ **Control injection site:**<br><br>○ **If paravenous: whitish bubble appears under the skin. → New attempt proximal to current site.**<br><br>○ **If intravenous: blood proximal to the injection site gives way to the injected fluid.**<br><br>→ **Remove needle, apply pressure with a clean gauze to stop bleeding, throw needle into the sharps container.**<br><br>→ **Observe injection site and animal closely.**	

Blood sampling from the lateral tail vein

**Material**<br>➔ Gloves<br>➔ Needle (27G)<br>➔ 1 ml syringe<br>➔ Warm water<br>➔ Water bin<br>➔ Sharps container<br>➔ Restrainer/ anesthesia<br>➔ Gauze<br>➔ Alcohol/Antispetic solution	**Preparation**<br>➔ Put on gloves.<br>Already prepared<br>✓ Warm up water in bin,<br>○ CAVE: do not heat too much!<br>✓ Anesthetise/ Restrain animal<br>✓ Warm up the tail in the water. ➔ veins dilate<br>✓ Dry the tail (gauze).<br>✓ Clean the tail with an alcoholic gauze (Use each gauze only once to wipe distally.)
**Techniques**	
**Puncture of the lateral tail vein**<br><br>➔ Hold the tail between your non-dominant hand 's thumb and index finger.<br><br>➔ Visualize the lateral tail veins.<br><br>➔ Stabilize the vein with your thumb and index finger and use them as a guideline for puncture and injection.<br><br>➔ Start the first puncture in the distal third of the tail.<br><br>➔ Insert the needle parallel to the tail, bevel of the needle facing upwards<br><br>➔ Penetrate 2-4 mm into the lumen of the visualized lateral tail vein.	Empty syringe for blood sampling!

**In the live animal:**<br><br>○ **If no blood flows, try again.**<br><br>○ **If you entered the vein correctly, blood will flow back into the needle hub. → Blood sampling is possible.**	
**<u>Blood sampling from the lateral tail vein</u>**<br><br>➔ **Blood can be sampled with a syringe or capillary tube:**<br>*(For the simulator, a syringe is recommended.)*<br><br>**CAVE: Aspiration may cause the vein to collapse!**<br>➔ **Work very slowly and carefully!**<br><br>○ **Max. volume for daily blood sampling: 0,7 ml/kg/day.**<br><br>➔ **Remove needle, apply pressure with a clean gauze to stop bleeding, throw needle into the sharps container.**<br><br>➔ **Observe injection site and animal closely.**	

Training mit Simulatoren

Gruppe Simulator A

Merkmale:

- Brust und Abdomen transparent
- Erfolgskontrolle: Bei Applikation in den Magen→ Flüssigkeitsaustritt an vorgesehener Öffnung
- Applikation von Flüssigkeit für alle Techniken

Vorbereitendes Training für

- Handling
- Fixieren (4 Griffe)
- Applikation per os mittels Sonde
- Applikation intravenös in die laterale Schwanzvene
- Blutentnahme aus der lateralen Schwanzvene

Freie Universität Berlin

Handling Ratte – Transfer von Käfig zu Käfig

Transfer von Käfig zu Käfig – Ratten nicht am Schwanz hochheben!

**Material:**	**Vorbereitung:**
➔ Handschuhe	➔ Handschuhe anziehen.<br>➔ Käfig öffnen.<br>➔ Wasserflasche und Käfiggitter zu Seite stellen.<br>➔ Tiere an neue Situation gewöhnen lassen.
**Technik**	
➔ Ratte um die Schultern greifen:<br><br>○ Daumen unter der Schulter auf einer Seite<br><br>○ Restliche Finger auf der anderen Seite. Zeigefinger über der Schulter<br><br>➔ Ratte vorsichtig mit einer Hand hochheben und Körper mit Fingerspitzen unterstützen.<br><br>➔ Ratte auf Unterarm/ Brust/ geeigneter Unterlage absetzen.	

**<u>Bei sehr lebhaften Tieren:</u>**<br><br>➔ Ratte an der Schwanzbasis zwischen Daumen, Zeige- und Mittelfinger fixieren.<br><br>➔ Mit der anderen Hand Ratte um die Schultern greifen:<br><br>o Daumen unter der Schulter auf einer Seite.<br><br>o Restliche Finger auf anderer Seite. Zeigefinger über der Schulter.<br><br>➔ Ratte vorsichtig hochheben und Körper mit Fingerspitzen unterstützen.<br><br>➔ Auf Unterarm/ Schulter/ geeigneter Unterlage absetzen.	

Fixieren Ratten I – "Fixieren mit Nackengriff"

**Material**	**Vorbereitung**
➔ Handschuhe	➔ Handschuhe anziehen<br>➔ Käfig öffnen.<br>➔ Wasser und Käfigdeckel zur Seite legen.<br>➔ Ratten an neue Situation gewöhnen lassen.
**Technik**	

➔ Ratte an der Schwanzbasis mit der dominanten Hand halten.

➔ Ratte an der Schwanzbasis sehr vorsichtig(!) auf einer rauen Unterlage zurückziehen.

➔ Die Ratte mit der nicht-dominanten Hand von hinten kommend fixieren:

- Die Ratte leicht (!) nach unten drücken.
- Daumen auf einer Seite, die restlichen Finger auf der anderen Seite platzieren.
- Mit Daumen und Zeigefinger die lose Haut im Nacken direkt hinter den Ohren zu einer Hautfalte greifen.
- Anschließend mit den restlichen Fingern eine Hautfalte mit der losen Haut am greifen.

➔ Atmung kontrollieren! Brust nicht komprimieren!

➔ **Manche Ratten können bei dieser Methode „schreien".**	

Fixieren Ratten II – "über der Schulter-Griff"

Herkömmliche Methode um Ratten für Eingriffe zu fixieren

**Material**	**Vorbereitung**
➔ Handschuhe	➔ Handschuhe anziehen<br>➔ Käfig öffnen.<br>➔ Wasser und Käfigdeckel zur Seite legen.<br>➔ Ratten an neue Situation gewöhnen lassen.

Technik

➔ Ratte an der Schwanzbasis mit der dominanten Hand halten.

➔ Ratte an der Schwanzbasis sehr vorsichtig(!) auf einer rauen Unterlage zurückziehen.

➔ Ratte mit der nicht-dominanten Hand von hinten kommend über den Schulterblättern greifen:

- Der Kopf liegt, so nah wie möglich an der Fingerbasis, zwischen Zeige- und Mittelfinger.
- Mit Ringfinger, kleinem Finger und Daumen Brust unterstüzen.

➔ Atmung kontrollieren! Brust nicht komprimieren!

➔ Den Körper mit der anderen Hand stabilisieren:

- Die Ratte um den Hinterleib greifen.
- Mit Daumen und Zeigefinger die Hinterbeine leicht nach unten drücken.

Fixieren von Ratten III – "mittlerer Schultergriff"

Herkömmliche Methode um Ratten für Eingriffe zu fixieren

Material	Vorbereitung
➔ Handschuhe	➔ Handschuhe anziehen<br>➔ Käfig öffnen.<br>➔ Wasser und Käfigdeckel zur Seite legen.<br>➔ Ratten an neue Situation gewöhnen lassen.

Technik

➔ Ratte an der Schwanzbasis mit der dominanten Hand halten.

➔ Ratte an der Schwanzbasis sehr vorsichtig(!) auf einer rauen Unterlage zurückziehen.

➔ Ratte um die Schultern greifen:

- Daumen unter der Schulter auf einer Seite
- Restliche Finger auf der anderen Seite. Zeigefinger über der Schulter.

➔ Daumen auf Zeigerfinger zubewegen und somit unter dem Kiefer den Kopf kontrollieren.

➔ Mit restliche Fingerspitzen die Brust unterhalb der Vorderbeine unterstützen.

➔ Atmung kontrollieren! Brust nicht komprimieren!

➔ **Den Körper mit der anderen Hand stabilisieren:**<br><br>○ **Die Ratte um den Hinterleib greifen.**<br>○ **Mit Daumen und Zeigefinger die Hinterbeine leicht nach unten drücken.**	

Fixieren Ratten IV – "unter der Schultergriff

Herkömmliche Methode um Ratten für Eingriffe zu fixieren

**Material**	**Vorbereitung**
➔ Handschuhe	➔ Handschuhe anziehen<br>➔ Käfig öffnen.<br>➔ Wasser und Käfigdeckel zur Seite legen.<br>➔ Ratten an neue Situation gewöhnen lassen.
**Technik**	

➔ Ratte an der Schwanzbasis mit der dominanten Hand halten.

➔ Ratte an der Schwanzbasis sehr vorsichtig(!) auf einer rauen Unterlage zurückziehen.

➔ Ratte mit der nicht-dominanten Hand von hinten kommend um die Schultern greifen:

- Daumen auf einer Seite, unter dem Vorderbein
- Restliche Finger auf der anderen Seite, unter dem Vorderbein.

➔ Die Vorderbeine mit Daumen und Zeigerfinger leicht nach vorne drücken.
➔ Die Vorderbeine sollten sich vor der Brust verschränken und somit ein Beißen verhindern.

➔ Atmung kontrollieren! Brust nicht komprimieren!

➔ **Den Körper mit der anderen Hand stabilisieren:**<br><br>○ **Die Ratte um den Hinterleib greifen.**<br>○ **Mit Daumen und Zeigefinger die Hinterbeine leicht nach unten drücken.**	

Applikation *per os* mittels Schlundsonde

Enterale Applikation

Material	Vorbereitung
➔ Handschuhe<br>➔ Schlundsonde (20-22 G x2-inch)<br>➔ 1 ml Spritze<br>➔ Medium *(für* Simulator E *nur Luft!)*<br>➔ (Marker)	➔ Handschuhe anziehen<br>Bereits erledigt<br>✓ Medium in Spritze aufziehen.
Methodik	
**Richtige Sondenlänge abmessen**<br><br>➔ Tier sicher mit Nackengriff fixieren.<br>○ Richtige Länge = Abstand zwischen Nasenspitze und Rippenbogen.<br><br>➔ Abstand falls nötig markieren.<br>○ (CAVE! Chemische Zusammensetzung Marker!)<br><br>**Einführen der Schlundsonde**<br><br>➔ Tier sicher (!) in vertikaler Position fixieren.<br><br>➔ Sonde neben Schneidezähnen, am oberen Gaumen entlang, Richtung Kehlkopf vorführen.<br>(echtes Tier: Schluckreflex)<br><br>➔ Den Kopf des Tieres vorsichtig gerade richten➔ Sonde gleitet von selbst in die Speiseröhre.<br><br>➔ Schlundsonde bis in den Magen vorführen.<br>○ Die Sonde gleitet fast von selbst den Ösophagus entlang.	

CAVE!

➔ **Niemals Gewalt anwenden!**

➔ **Bei Widerstand: Stop! Sonde entfernen, erneut versuchen!**

➔ **Atmung immer kontrollieren!**

➔ **Bei unregelmäßiger Atmung:**

- **Eingriff sofort stoppen!**
- **Tier in den Käfig zurücksetzen und erneut versuchen, wenn sich das Tier erholt hat.**

Bolusgabe und Entfernen der Sonde

Bei regelmäßiger Atmung und Lage im Magen:

➔ **Eingabe von Medium (max. 10 ml/kg) langsam und stetig.**

➔ **Sonde dem Verlauf folgend entfernen.**

➔ **Das Tier vorsichtig in den Käfig zurücksetzen.**

➔ **Tier und Atmung beobachten.**

Applikation intravenös in die laterale Schwanzvene

**Material**<br>➔ Handschuhe<br>➔ Nadel (27G)<br>➔ 1 ml Spritze<br>➔ Medium<br>➔ Warmes Wasser<br>➔ Wasserbehälter<br>➔ Abwurfcontainer<br>➔ Restrainer/ Anästhesie<br>➔ Tupfer<br>➔ Alkohohllösung o. antiseptische Lösung	**Vorbereitung**<br>➔ Handschuhe anziehen.<br>Bereits erledigt:<br>✓ 1 ml Spritze mit Medium füllen:<br>➔ Luftblasen vermeiden.<br>✓ Wasser im Behälter erwärmen.<br>○ CAVE: Nicht zu stark erhitzen!<br>✓ Tier im Restrainer fixieren. / Tier anästhesieren.<br>✓ Schwanz im Wasserbad erwärmen ➔ Dilatation der Venen.<br>✓ Schwanz trocknen<br>✓ Schwanz mit Alkoholtupfer reinigen. (pro Tupfer nur einmal nach distal wischen!)
**Technik**	
**Punktion der lateralen Schwanzvene**<br><br>➔ Den Schwanz zwischen Daumen und Zeigerfinger der nicht-dominaten Hand halten.<br><br>➔ Die lateralen Venen erkennen.<br><br>➔ Den Schwanz zwischen Daumen und Zeigefinger fixieren und die Finger als Führungslinie zur Punktion und Injektion nutzen.<br><br>➔ Mit der ersten Punktion im distalen Drittel des Schwanzes beginnen.<br><br>➔ Parallel zum Schwanz einstechen, Anschliff der Nadel zeigt nach oben.<br><br>➔ 2-4 mm in das Lumen der lateralen Vene einstechen.	

**Am lebenden Tier:**<br><br>○ **Kein Blut im Konus sichtbar → neuer Versuch proximal der Einstichstelle.**<br><br>○ **Vene getroffen → Blutfluss in den Ansatz des Konus → intravenöse Applikation möglich.**	
**<u>Intravenöse Applikation in die Schwanzvene:</u>**<br><br>○ **Medium langsam injizeren: max. 5 ml/kg Gewicht.**<br><br>○ **Injektion muss ohne Widerstand möglich sein!**<br><br>○ **Injektionstelle kontrollieren:**<br><br>○ **Paravenöse Injektion: weißliche Blasen erscheinen unter der Haut → neuer Versuch proximal der Einstichstelle.**<br><br>○ **Intravenöse Injektion: Blut proximal der Einstichstelle weicht der injezierten Flüssigkeit.**<br><br>→ **Nadel entfernen, mit Tupfer auf Einstichstelle drücken um Blutung zu stoppen, Nadel im Abwurfkontainer entsorgen.**<br><br>→ **Injektionsstelle und Tier genau beobachten.**	

Freie Universität Berlin

Blutentnahme an der lateralen Schwanzvene

Material

- ➔ Handschuhe
- ➔ Nadel (27G)
- ➔ 1ml Spritze
- ➔ Warmes Wasser
- ➔ Wasserbehälter
- ➔ Tupfer
- ➔ Alkohol-/Desinfektionslösung
- ➔ Abwurfbehälter
- ➔ Retrainer, Narkose

Vorbereitung

- ➔ Handschuhe anziehen.

Bereits erledigt

- ✓ Wasser im Behälter erwärmen.
  - CAVE: Nicht zu stark erhitzen!
- ✓ Tier im Restrainer fixieren/ narkotisieren
- ✓ Schwanz im warmen Wasserbad erwärmen-> Dilatation der Venen
- ✓ Schwanz trocknen
- ✓ Schwanz mit in Alkohol getränkten Tupfer desinfizieren (pro Tupfer 1x nach distal wischen.)

Methodik

Punktion der lateralen Schwanzvene

- ➔ Den Schwanz in Daumen und Zeigefinger der nicht-dominanten Hand halten.
- ➔ Verlauf der seitlichen Venen betrachten.
- ➔ Schwanz mit Zeigefinger und Daumen der nicht-dominanten Hand stabilisieren und als Führungslinie für Punktion und Applikation nutzen.
- ➔ Mit der ersten Punktion im distalen Drittel des Schwanzes beginnen.
- ➔ Die Nadel parallel zum Schwanz einstechen, Anschliff nach oben um die laterale Vene zu treffen.

Leere Spritze für Blutentnahme!

**Beim lebenden Tier:**<br><br>○ **Kein Bluteinstrom in den Nadelansatz→ nochmal versuchen.**<br><br>○ **Wenn die Vene getroffen wurde→ Bluteinfluss in den Nadelansatz→ Blutentnahme möglich.**	
**2.**	
**Blutentnahme aus der lateralen Schwanzvene**<br><br>➔ **Einschießendes Blut mittels Kappillarröhrchen oder Spritze aufnehmen.**<br>*(für den Simulator empfiehlt sich die Spritze.)*<br><br>**CAVE: Durch Aspiration kann die Vene kollabieren!**<br>**--> Sehr langsam und vorsichtig arbeiten!**<br><br>○ **Max. Volumen bei täglicher Blutentnahme: 0,7 ml/kg/Tag.**<br><br>➔ **Nadel entfernen, Tupfer auf Einstichstelle drücken, Nadel in den Abwurfbehälter.**<br><br>➔ **Einstichstelle und Tier beobachten.**	

Training mit Simulatoren

Gruppe Simulator B

Merkmale:

- Brust und Abdomen transparent
- Erfolgskontrolle: Bei Applikation in den Magen→ Flüssigkeitsaustritt an vorgesehener Öffnung
- Applikation von Flüssigkeit für alle Techniken

Vorbereitendes Training für

- Handling
- Fixieren (4 Griffe)
- Applikation per os mittels Sonde
- Applikation intravenös in die laterale Schwanzvene
- Blutentnahme aus der lateralen Schwanzvene

Freie Universität Berlin

Handling Ratte – Transfer von Käfig zu Käfig

Transfer von Käfig zu Käfig – Ratten nicht am Schwanz hochheben!

**Material:**<br>➔ Handschuhe	**Vorbereitung:**<br>➔ Handschuhe anziehen.<br>➔ Käfig öffnen.<br>➔ Wasserflasche und Käfiggitter zu Seite stellen.<br>➔ Tiere an neue Situation gewöhnen lassen.
**Technik**	
➔ Ratte um die Schultern greifen:<br>○ Daumen unter der Schulter auf einer Seite<br>○ Restliche Finger auf der anderen Seite. Zeigefinger über der Schulter<br>➔ Ratte vorsichtig mit einer Hand hochheben und Körper mit Fingerspitzen unterstützen.<br>➔ Ratte auf Unterarm/ Brust/ geeigneter Unterlage absetzen.	

**<u>Bei sehr lebhaften Tieren:</u>**<br><br>➔ **Ratte an der Schwanzbasis zwischen Daumen, Zeige- und Mittelfinger fixieren.**<br><br>➔ **Mit der anderen Hand Ratte um die Schultern greifen:**<br><br>o **Daumen unter der Schulter auf einer Seite.**<br><br>o **Restliche Finger auf anderer Seite. Zeigefinger über der Schulter.**<br><br>➔ **Ratte vorsichtig hochheben und Körper mit Fingerspitzen unterstützen.**<br><br>➔ **Auf Unterarm/ Schulter/ geeigneter Unterlage absetzen.**	

Fixieren Ratten I – "Fixieren mit Nackengriff"

**Material**	**Vorbereitung**
➔ Handschuhe	➔ Handschuhe anziehen<br>➔ Käfig öffnen.<br>➔ Wasser und Käfigdeckel zur Seite legen.<br>➔ Ratten an neue Situation gewöhnen lassen.

Technik

➔ Ratte an der Schwanzbasis mit der dominanten Hand halten.

➔ Ratte an der Schwanzbasis sehr vorsichtig(!) auf einer rauen Unterlage zurückziehen.

➔ Die Ratte mit der nicht-dominanten Hand von hinten kommend fixieren:

- Die Ratte leicht (!) nach unten drücken.
- Daumen auf einer Seite, die restlichen Finger auf der anderen Seite platzieren.
- Mit Daumen und Zeigefinger die lose Haut im Nacken direkt hinter den Ohren zu einer Hautfalte greifen.
- Anschließend mit den restlichen Fingern eine Hautfalte mit der losen Haut am greifen.

➔ Atmung kontrollieren! Brust nicht komprimieren!

➔ **Manche Ratten können bei dieser Methode „schreien“.**	

Freie Universität Berlin

Fixieren Ratten II – "über der Schulter-Griff"

Herkömmliche Methode um Ratten für Eingriffe zu fixieren

**Material**	**Vorbereitung**
➔ Handschuhe	➔ Handschuhe anziehen<br>➔ Käfig öffnen.<br>➔ Wasser und Käfigdeckel zur Seite legen.<br>➔ Ratten an neue Situation gewöhnen lassen.
**Technik**	

➔ Ratte an der Schwanzbasis mit der dominanten Hand halten.

➔ Ratte an der Schwanzbasis sehr vorsichtig(!) auf einer rauen Unterlage zurückziehen.

➔ Ratte mit der nicht-dominanten Hand von hinten kommend über den Schulterblättern greifen:

- Der Kopf liegt, so nah wie möglich an der Fingerbasis, zwischen Zeige- und Mittelfinger.
- Mit Ringfinger, kleinem Finger und Daumen Brust unterstüzen.

➔ Atmung kontrollieren! Brust nicht komprimieren!

➔ Den Körper mit der anderen Hand stabilisieren:

- Die Ratte um den Hinterleib greifen.
- Mit Daumen und Zeigefinger die Hinterbeine leicht nach unten drücken.

Freie Universität Berlin

Fixieren von Ratten III – “mittlerer Schultergriff“

Herkömmliche Methode um Ratten für Eingriffe zu fixieren

**Material**	**Vorbereitung**
➔ Handschuhe	➔ Handschuhe anziehen<br>➔ Käfig öffnen.<br>➔ Wasser und Käfigdeckel zur Seite legen.<br>➔ Ratten an neue Situation gewöhnen lassen.
**Technik**	
➔ Ratte an der Schwanzbasis mit der dominanten Hand halten.<br><br>➔ Ratte an der Schwanzbasis sehr vorsichtig(!) auf einer rauen Unterlage zurückziehen.<br><br>➔ Ratte um die Schultern greifen:<br><br>○ Daumen unter der Schulter auf einer Seite<br><br>○ Restliche Finger auf der anderen Seite. Zeigefinger über der Schulter.<br><br>➔ Daumen auf Zeigerfinger zubewegen und somit unter dem Kiefer den Kopf kontrollieren.<br><br>➔ Mit restliche Fingerspitzen die Brust unterhalb der Vorderbeine unterstützen.<br><br>➔ Atmung kontrollieren! Brust nicht komprimieren!	

➔ **Den Körper mit der anderen Hand stabilisieren:**<br><br>○ **Die Ratte um den Hinterleib greifen.**<br>○ **Mit Daumen und Zeigefinger die Hinterbeine leicht nach unten drücken.**	

Fixieren Ratten IV – “unter der Schultergriff

Herkömmliche Methode um Ratten für Eingriffe zu fixieren

Material	Vorbereitung
➔ Handschuhe	➔ Handschuhe anziehen<br>➔ Käfig öffnen.<br>➔ Wasser und Käfigdeckel zur Seite legen.<br>➔ Ratten an neue Situation gewöhnen lassen.

Technik

➔ Ratte an der Schwanzbasis mit der dominanten Hand halten.

➔ Ratte an der Schwanzbasis sehr vorsichtig(!) auf einer rauen Unterlage zurückziehen.

➔ Ratte mit der nicht-dominanten Hand von hinten kommend um die Schultern greifen:

- Daumen auf einer Seite, unter dem Vorderbein
- Restliche Finger auf der anderen Seite, unter dem Vorderbein.

➔ Die Vorderbeine mit Daumen und Zeigerfinger leicht nach vorne drücken.

➔ Die Vorderbeine sollten sich vor der Brust verschränken und somit ein Beißen verhindern.

➔ Atmung kontrollieren! Brust nicht komprimieren!

➔ **Den Körper mit der anderen Hand stabilisieren:**<br><br>○ **Die Ratte um den Hinterleib greifen.**<br>○ **Mit Daumen und Zeigefinger die Hinterbeine leicht nach unten drücken.**	

Applikation *per os* mittels Schlundsonde

Enterale Applikation

Material	Vorbereitung
➔ Handschuhe<br>➔ Schlundsonde (20-22 G x2-inch)<br>➔ 1 ml Spritze<br>➔ Medium (*für* Simulator E *nur Luft!)*<br>➔ (Marker)	➔ Handschuhe anziehen<br>Bereits erledigt<br>✓ Medium in Spritze aufziehen.
Methodik	
**Richtige Sondenlänge abmessen**<br><br>➔ Tier sicher mit Nackengriff fixieren.<br>○ Richtige Länge = Abstand zwischen Nasenspitze und Rippenbogen.<br><br>➔ Abstand falls nötig markieren.<br>○ (CAVE! Chemische Zusammensetzung Marker!)<br><br>**Einführen der Schlundsonde**<br><br>➔ Tier sicher (!) in vertikaler Position fixieren.<br><br>➔ Sonde neben Schneidezähnen, am oberen Gaumen entlang, Richtung Kehlkopf vorführen.<br>(echtes Tier: Schluckreflex)<br><br>➔ Den Kopf des Tieres vorsichtig gerade richten➔ Sonde gleitet von selbst in die Speiseröhre.<br><br>➔ Schlundsonde bis in den Magen vorführen.<br>○ Die Sonde gleitet fast von selbst den Ösophagus entlang.	

CAVE!

➔ **Niemals Gewalt anwenden!**

➔ **Bei Widerstand: Stop! Sonde entfernen, erneut versuchen!**

➔ **Atmung immer kontrollieren!**

➔ **Bei unregelmäßiger Atmung:**

- **Eingriff sofort stoppen!**
- **Tier in den Käfig zurücksetzen und erneut versuchen, wenn sich das Tier erholt hat.**

Bolusgabe und Entfernen der Sonde

Bei regelmäßiger Atmung und Lage im Magen:

➔ Eingabe von Medium (max. 10 ml/kg) langsam und stetig.

➔ Sonde dem Verlauf folgend entfernen.

➔ Das Tier vorsichtig in den Käfig zurücksetzen.

➔ Tier und Atmung beobachten.

Applikation intravenös in die laterale Schwanzvene

Material	Vorbereitung
→ Handschuhe<br>→ Nadel (27G)<br>→ 1 ml Spritze<br>→ Medium<br>→ Warmes Wasser<br>→ Wasserbehälter<br>→ Abwurfcontainer<br>→ Restrainer/ Anästhesie<br>→ Tupfer<br>→ Alkohohllösung o. antiseptische Lösung	→ Handschuhe anziehen.<br>Bereits erledigt:<br>✓ 1 ml Spritze mit Medium füllen: →Luftblasen vermeiden.<br>✓ Wasser im Behälter erwärmen.<br>o CAVE: Nicht zu stark erhitzen!<br>✓ Tier im Restrainer fixieren. / Tier anästhesieren.<br>✓ Schwanz im Wasserbad erwärmen → Dilatation der Venen.<br>✓ Schwanz trocknen<br>✓ Schwanz mit Alkoholtupfer reinigen. (pro Tupfer nur einmal nach distal wischen!)
Technik	
Punktion der lateralen Schwanzvene<br><br>→ Den Schwanz zwischen Daumen und Zeigerfinger der nicht-dominaten Hand halten.<br><br>→ Die lateralen Venen erkennen.<br><br>→ Den Schwanz zwischen Daumen und Zeigefinger fixieren und die Finger als Führungslinie zur Punktion und Injektion nutzen.<br><br>→ Mit der ersten Punktion im distalen Drittel des Schwanzes beginnen.<br><br>→ Parallel zum Schwanz einstechen, Anschliff der Nadel zeigt nach oben.<br><br>→ 2-4 mm in das Lumen der lateralen Vene einstechen.	

Am lebenden Tier:

- Kein Blut im Konus sichtbar → neuer Versuch proximal der Einstichstelle.
- Vene getroffen → Blutfluss in den Ansatz des Konus → intravenöse Applikation möglich.

Intravenöse Applikation in die Schwanzvene:

- Medium langsam injizeren: max. 5 ml/kg Gewicht.
    - Injektion muss ohne Widerstand möglich sein!
- Injektionstelle kontrollieren:
    - Paravenöse Injektion: weißliche Blasen erscheinen unter der Haut → neuer Versuch proximal der Einstichstelle.
    - Intravenöse Injektion: Blut proximal der Einstichstelle weicht der injezierten Flüssigkeit.

→ Nadel entfernen, mit Tupfer auf Einstichstelle drücken um Blutung zu stoppen, Nadel im Abwurfkontainer entsorgen.

→ Injektionsstelle und Tier genau beobachten.

Blutentnahme an der lateralen Schwanzvene

Material

- ➔ Handschuhe
- ➔ Nadel (27G)
- ➔ 1ml Spritze
- ➔ Warmes Wasser
- ➔ Wasserbehälter
- ➔ Tupfer
- ➔ Alkohol-/Desinfektionslösung
- ➔ Abwurfbehälter
- ➔ Retrainer, Narkose

Vorbereitung

- ➔ Handschuhe anziehen.

Bereits erledigt

- ✓ Wasser im Behälter erwärmen.
  - CAVE: Nicht zu stark erhitzen!
- ✓ Tier im Restrainer fixieren/ narkotisieren
- ✓ Schwanz im warmen Wasserbad erwärmen-> Dilatation der Venen
- ✓ Schwanz trocknen
- ✓ Schwanz mit in Alkohol getränkten Tupfer desinfizieren
  (pro Tupfer 1x nach distal wischen.)

Methodik

Punktion der lateralen Schwanzvene

- ➔ Den Schwanz in Daumen und Zeigefinger der nicht-dominanten Hand halten.
- ➔ Verlauf der seitlichen Venen betrachten.
- ➔ Schwanz mit Zeigefinger und Daumen der nicht-dominanten Hand stabilisieren und als Führungslinie für Punktion und Applikation nutzen.
- ➔ Mit der ersten Punktion im distalen Drittel des Schwanzes beginnen.
- ➔ Die Nadel parallel zum Schwanz einstechen, Anschliff nach oben um die laterale Vene zu treffen.

Leere Spritze für Blutentnahme!

**Beim lebenden Tier:**<br><br>○ **Kein Bluteinstrom in den Nadelansatz→ nochmal versuchen.**<br><br>○ **Wenn die Vene getroffen wurde→ Bluteinfluss in den Nadelansatz→ Blutentnahme möglich.**	
**2.**	
**Blutentnahme aus der lateralen Schwanzvene**<br><br>➔ **Einschießendes Blut mittels Kappillarröhrchen oder Spritze aufnehmen.**<br>*(für den Simulator empfiehlt sich die Spritze.)*<br><br>**CAVE: Durch Aspiration kann die Vene kollabieren!**<br>**--> Sehr langsam und vorsichtig arbeiten!**<br><br>○ **Max. Volumen bei täglicher Blutentnahme: 0,7 ml/kg/Tag.**<br><br>➔ **Nadel entfernen, Tupfer auf Einstichstelle drücken, Nadel in den Abwurfbehälter.**<br><br>➔ **Einstichstelle und Tier beobachten.**	

Training mit Simulatoren

Die Simulator C

Vorbereitendes Training für

- Handling
- Fixieren (4 Griffe)
- Blutentnahme aus der Vena saphena
- Blutentnahme aus dem Herzen
- Applikation intravenös in die laterale Schwanzvene
- Blutentnahme aus der lateralen Schwanzvene

Handling Ratte – Transfer von Käfig zu Käfig

Transfer von Käfig zu Käfig – Ratten nicht am Schwanz hochheben!

**Material:**	**Vorbereitung:**
➔ Handschuhe	➔ Handschuhe anziehen.<br>➔ Käfig öffnen.<br>➔ Wasserflasche und Käfiggitter zu Seite stellen.<br>➔ Tiere an neue Situation gewöhnen lassen.
**Technik**	
➔ Ratte um die Schultern greifen:<br>○ Daumen unter der Schulter auf einer Seite<br>○ Restliche Finger auf der anderen Seite. Zeigefinger über der Schulter<br>➔ Ratte vorsichtig mit einer Hand hochheben und Körper mit Fingerspitzen unterstützen.<br>➔ Ratte auf Unterarm/ Brust/ geeigneter Unterlage absetzen.	

**<u>Bei sehr lebhaften Tieren:</u>**<br><br>➔ **Ratte an der Schwanzbasis zwischen Daumen, Zeige- und Mittelfinger fixieren.**<br><br>➔ **Mit der anderen Hand Ratte um die Schultern greifen:**<br><br>o **Daumen unter der Schulter auf einer Seite.**<br><br>o **Restliche Finger auf anderer Seite. Zeigefinger über der Schulter.**<br><br>➔ **Ratte vorsichtig hochheben und Körper mit Fingerspitzen unterstützen.**<br><br>➔ **Auf Unterarm/ Schulter/ geeigneter Unterlage absetzen.**	

Freie Universität Berlin

Fixieren Ratten I – "Fixieren mit Nackengriff"

Material	Vorbereitung
➔ Handschuhe	➔ Handschuhe anziehen<br>➔ Käfig öffnen.<br>➔ Wasser und Käfigdeckel zur Seite legen.<br>➔ Ratten an neue Situation gewöhnen lassen.

Technik

➔ Ratte an der Schwanzbasis mit der dominanten Hand halten.

➔ Ratte an der Schwanzbasis sehr vorsichtig(!) auf einer rauen Unterlage zurückziehen.

➔ Die Ratte mit der nicht-dominanten Hand von hinten kommend fixieren:

- Die Ratte leicht (!) nach unten drücken.
- Daumen auf einer Seite, die restlichen Finger auf der anderen Seite platzieren.
- Mit Daumen und Zeigefinger die lose Haut im Nacken direkt hinter den Ohren zu einer Hautfalte greifen.
- Anschließend mit den restlichen Fingern eine Hautfalte mit der losen Haut am greifen.

➔ Atmung kontrollieren! Brust nicht komprimieren!

➔ **Manche Ratten können bei dieser Methode „schreien“.**	

Freie Universität Berlin

Fixieren Ratten II – "über der Schulter-Griff"

Herkömmliche Methode um Ratten für Eingriffe zu fixieren

**Material**	**Vorbereitung**
➔ Handschuhe	➔ Handschuhe anziehen<br>➔ Käfig öffnen.<br>➔ Wasser und Käfigdeckel zur Seite legen.<br>➔ Ratten an neue Situation gewöhnen lassen.
**Technik**	

➔ Ratte an der Schwanzbasis mit der dominanten Hand halten.

➔ Ratte an der Schwanzbasis sehr vorsichtig(!) auf einer rauen Unterlage zurückziehen.

➔ Ratte mit der nicht-dominanten Hand von hinten kommend über den Schulterblättern greifen:

- Der Kopf liegt, so nah wie möglich an der Fingerbasis, zwischen Zeige- und Mittelfinger.
- Mit Ringfinger, kleinem Finger und Daumen Brust unterstüzen.

➔ Atmung kontrollieren! Brust nicht komprimieren!

➔ Den Körper mit der anderen Hand stabilisieren:

  - Die Ratte um den Hinterleib greifen.
  - Mit Daumen und Zeigefinger die Hinterbeine leicht nach unten drücken.

Freie Universität Berlin

Fixieren von Ratten III – “mittlerer Schultergriff“

Herkömmliche Methode um Ratten für Eingriffe zu fixieren

**Material**	**Vorbereitung**
➔ Handschuhe	➔ Handschuhe anziehen<br>➔ Käfig öffnen.<br>➔ Wasser und Käfigdeckel zur Seite legen.<br>➔ Ratten an neue Situation gewöhnen lassen.

Technik

➔ Ratte an der Schwanzbasis mit der dominanten Hand halten.

➔ Ratte an der Schwanzbasis sehr vorsichtig(!) auf einer rauen Unterlage zurückziehen.

➔ Ratte um die Schultern greifen:

- Daumen unter der Schulter auf einer Seite
- Restliche Finger auf der anderen Seite. Zeigefinger über der Schulter.

➔ Daumen auf Zeigerfinger zubewegen und somit unter dem Kiefer den Kopf kontrollieren.

➔ Mit restliche Fingerspitzen die Brust unterhalb der Vorderbeine unterstützen.

➔ Atmung kontrollieren! Brust nicht komprimieren!

➔ **Den Körper mit der anderen Hand stabilisieren:**<br><br>○ **Die Ratte um den Hinterleib greifen.**<br>○ **Mit Daumen und Zeigefinger die Hinterbeine leicht nach unten drücken.**	

Fixieren Ratten IV – “unter der Schultergriff“

Herkömmliche Methode um Ratten für Eingriffe zu fixieren

Material	Vorbereitung
➔ Handschuhe	➔ Handschuhe anziehen<br>➔ Käfig öffnen.<br>➔ Wasser und Käfigdeckel zur Seite legen.<br>➔ Ratten an neue Situation gewöhnen lassen.
**Technik**	
➔ Ratte an der Schwanzbasis mit der dominanten Hand halten.<br><br>➔ Ratte an der Schwanzbasis sehr vorsichtig(!) auf einer rauen Unterlage zurückziehen.<br><br>➔ Ratte mit der nicht-dominanten Hand von hinten kommend um die Schultern greifen:<br><br>○ Daumen auf einer Seite, unter dem Vorderbein<br><br>○ Restliche Finger auf der anderen Seite, unter dem Vorderbein.<br><br>➔ Die Vorderbeine mit Daumen und Zeigerfinger leicht nach vorne drücken.<br>➔ Die Vorderbeine sollten sich vor der Brust verschränken und somit ein Beißen verhindern.<br><br>➔ Atmung kontrollieren! Brust nicht komprimieren!	

➔ **Den Körper mit der anderen Hand stabilisieren:**<br><br>○ **Die Ratte um den Hinterleib greifen.**<br>○ **Mit Daumen und Zeigefinger die Hinterbeine leicht nach unten drücken.**	

Freie Universität Berlin

Blutentnahme aus der Vena saphena

Nur für kleine Blutvolumina

**Material:**	**Vorbereitung**
➔ Handschuhe<br>➔ Elektrorasierer (lebendiges Tier)<br>➔ Alkoholtupfer (lebendiges Tier)<br>➔ Nadel (25G) / Lanzette (28G)<br>➔ Heparin- (Kapillar-) Röhrchen /Tube (lebendiges Tier)<br>➔ Anästhesie (lebendiges Tier)<br>➔ Tupfer (trocken)	✓ Handschuhe anziehen.<br>**Bereits erledigt:**<br>✓ Hilfsperson organisieren. / Tier anästhesieren.<br>✓ Hinterbein lateral rasieren.<br>✓ Injektionsstelle mit Alkoholtupfer desinfizieren.
**Technik**	
➔ Vene über dem Knie stauen. (nicht nötig beim Simulator).<br><br>➔ Vena saphena entdecken./ Verlauf betrachten.	

➔ **Vene im 90° Winkel zur Vene punktieren.**<br>○ **CAVE: Nicht durchstechen!**<br>○ **Max. 3 Versuche. Dann das Tier erholen lassen.**<br><br>➔ **Vene punktiert➔ Tropfen Blut wird sichtbar.**<br><br>➔ **Austretendes Blut mit Kappillarröhrchen/Tube auffangen.**<br>○ **Max. Volumen: 10 % des gesamten Blutvolumens.**<br><br>➔ **Venösen Stau lösen (nicht nötig für den Simulator).**<br><br>➔ **Kurz mit Tupfer auf die Injektionsstelle drücken, um Blutung zu stoppen.**<br><br>➔ **Injektionsstelle mit trockenem Tupfer abwischen.**<br><br>➔ **Injektionsstelle und Tier genau beobachten.**	

Herzpunktion (Blutentnahme aus dem Herzen)

Finale Blutentnahme für große Blutmengen

Tiefe Narkose und umgehende Euthanasie nach Blutentnahme obligat.

**Material:**	**Vorbereitung:**
➔ Handschuhe<br>➔ Tiefe Anästhesie beim lebenden Tier<br>➔ Nadel (25G)<br>➔ 3 ml Spritze	➔ Handschuhe anziehen.<br>➔ Tier anästhesieren.<br>(Tiefe Narkose➔ Reflexe überprüfen!)
**Technik**	
➔ Tier auf dem Rücken liegend auf einer stabilen Unterlage fixieren (z.B Operationstisch).<br><br>➔ Herz und Processus xyphoideus palpieren (Herz befindet sich 1 cm cranial des Processus xyphoideus).<br><br>Punktion vom Sternum aus<br><br>➔ Rippenbogen zwischen Daumen und Zeigerfinger der nicht-dominanen Hand festhalten.<br><br>➔ Nadel an Sternumspitze im 20 - 30° Winkel mit Anschliff nach oben einstechen: unterhalb oder links des Processus xyphoideus. Auf die linke Schulter zielen.<br><br>➔ Langsam aspirieren, während man die Nadel vorschiebt.<br><br>➔ Stoppen und Position halten bei Blutrückfluss in den Nadelansatz.<br><br>➔ Nadel und Spritze in Position halten, dabei langsam aspirieren bis gesamte Blutmenge entnommen ist.<br>○ Max. Blutentnahmevolumen 50 % des Gesamtblutes	

➔ Tier unverzüglich euthanasieren und Eintritt des Todes sicherstellen.<br><br>➔ Kein fließt Blut in den Nadelansatz:<br>○ Spritze zurückziehen und Einstichwinkel etwas ändern.<br>○ Langsam aspirieren während man die Nadel vorschiebt.	
<u>**Punktion über den linken Brustkorb**</u><br><br>➔ Rippenbogen zwischen Daumen und Zeigefinger der nicht-dominaten Hand halten.<br><br>➔ Nadel mit Anschliff nach oben im 45° Winkel in den Zwischenrippenraum auf Höhe des <u>linken</u> Ellenbogens einstechen.<br><br>➔ Langsam aspirieren während man die Nadel vorschiebt.<br><br>➔ Stoppen und Position halten bei Blutrückfluss in den Nadelansatz.<br><br>➔ Nadel und Spritze in Position halten, dabei langsam aspirieren bis gesamte Blutmenge entnommen ist.<br>○ Max. Blutentnahmevolumen 50 % des Gesamtblutes<br><br>➔ Tier unverzüglich euthanasieren und Eintritt des Todes sicherstellen.<br><br>➔ Kein Blut fließt in den Nadelansatz:<br>○ Spritze zurückziehen und Einstichwinkel etwas ändern.<br>○ Langsam aspirieren während man die Nadel vorschiebt.	

Applikation intravenös in die laterale Schwanzvene

<table>
<tr>
<td>
<u>Material</u>
➔ Handschuhe
➔ Nadel (27G)
➔ 1 ml Spritze
➔ Medium
➔ Warmes Wasser
➔ Wasserbehälter
➔ Abwurfcontainer
➔ Restrainer/ Anästhesie
➔ Tupfer
➔ Alkohohllösung o. antiseptische Lösung
</td>
<td>
<u>Vorbereitung</u>
➔ Handschuhe anziehen.
<u>Bereits erledigt:</u>
✓ 1 ml Spritze mit Medium füllen:
➔Luftblasen vermeiden.
✓ Wasser im Behälter erwärmen.
○ CAVE: Nicht zu stark erhitzen!
✓ Tier im Restrainer fixieren. / Tier anästhesieren.
✓ Schwanz im Wasserbad erwärmen ➔ Dilatation der Venen.
✓ Schwanz trocknen
✓ Schwanz mit Alkoholtupfer reinigen. (pro Tupfer nur einmal nach distal wischen!)
</td>
</tr>
<tr>
<td><u>Technik</u></td>
<td></td>
</tr>
<tr>
<td>
<u>Punktion der lateralen Schwanzvene</u>

➔ Den Schwanz zwischen Daumen und Zeigerfinger der nicht-dominaten Hand halten.

➔ Die lateralen Venen erkennen.

➔ Den Schwanz zwischen Daumen und Zeigefinger fixieren und die Finger als Führungslinie zur Punktion und Injektion nutzen.

➔ Mit der ersten Punktion im distalen Drittel des Schwanzes beginnen.

➔ Parallel zum Schwanz einstechen, Anschliff der Nadel zeigt nach oben.

➔ 2-4 mm in das Lumen der lateralen Vene einstechen.
</td>
<td></td>
</tr>
</table>

**Am lebenden Tier:**<br>◦ **Kein Blut im Konus sichtbar → neuer Versuch proximal der Einstichstelle.**<br>◦ **Vene getroffen → Blutfluss in den Ansatz des Konus → intravenöse Applikation möglich.**	
**<u>Intravenöse Applikation in die Schwanzvene:</u>**<br>◦ **Medium langsam injizeren: max. 5 ml/kg Gewicht.**<br>◦ **Injektion muss ohne Widerstand möglich sein!**<br>◦ **Injektionstelle kontrollieren:**<br>◦ **Paravenöse Injektion: weißliche Blasen erscheinen unter der Haut → neuer Versuch proximal der Einstichstelle.**<br>◦ **Intravenöse Injektion: Blut proximal der Einstichstelle weicht der injezierten Flüssigkeit.**<br>→ **Nadel entfernen, mit Tupfer auf Einstichstelle drücken um Blutung zu stoppen, Nadel im Abwurfkontainer entsorgen.**<br>→ **Injektionsstelle und Tier genau beobachten.**	

Freie Universität Berlin

Blutentnahme an der lateralen Schwanzvene

Material

- ➔ Handschuhe
- ➔ Nadel (27G)
- ➔ 1ml Spritze
- ➔ Warmes Wasser
- ➔ Wasserbehälter
- ➔ Tupfer
- ➔ Alkohol-/Desinfektionslösung
- ➔ Abwurfbehälter
- ➔ Retrainer, Narkose

Vorbereitung

- ➔ Handschuhe anziehen.

Bereits erledigt

- ✓ Wasser im Behälter erwärmen.
  - CAVE: Nicht zu stark erhitzen!
- ✓ Tier im Restrainer fixieren/ narkotisieren
- ✓ Schwanz im warmen Wasserbad erwärmen-> Dilatation der Venen
- ✓ Schwanz trocknen
- ✓ Schwanz mit in Alkohol getränkten Tupfer desinfizieren (pro Tupfer 1x nach distal wischen.)

Methodik

Punktion der lateralen Schwanzvene

- ➔ Den Schwanz in Daumen und Zeigefinger der nicht-dominanten Hand halten.
- ➔ Verlauf der seitlichen Venen betrachten.
- ➔ Schwanz mit Zeigefinger und Daumen der nicht-dominanten Hand stabilisieren und als Führungslinie für Punktion und Applikation nutzen.
- ➔ Mit der ersten Punktion im distalen Drittel des Schwanzes beginnen.
- ➔ Die Nadel parallel zum Schwanz einstechen, Anschliff nach oben um die laterale Vene zu treffen.

Leere Spritze für Blutentnahme!

**Beim lebenden Tier:**<br><br>○ **Kein Bluteinstrom in den Nadelansatz→ nochmal versuchen.**<br><br>○ **Wenn die Vene getroffen wurde→ Bluteinfluss in den Nadelansatz→ Blutentnahme möglich.**	
**2.**	
**Blutentnahme aus der lateralen Schwanzvene**<br><br>➔ **Einschießendes Blut mittels Kappillarröhrchen oder Spritze aufnehmen.**<br>*(für den Simulator empfiehlt sich die Spritze.)*<br><br>**CAVE: Durch Aspiration kann die Vene kollabieren!**<br>**--> Sehr langsam und vorsichtig arbeiten!**<br><br>○ **Max. Volumen bei täglicher Blutentnahme: 0,7 ml/kg/Tag.**<br><br>➔ **Nadel entfernen, Tupfer auf Einstichstelle drücken, Nadel in den Abwurfbehälter.**<br><br>➔ **Einstichstelle und Tier beobachten.**	

Training mit Simulatoren

Gruppe Simulator D

Vorbereitendes Training für

- Handling
- Fixieren (4 Griffe)
- Ohrlochmarkierung
- Applikation intravenös in die laterale Schwanzvene
- Blutentnahme aus der lateralen Schwanzvene

Merkmale:

- Beweglicher Kopf
- Applikation von Flüssigkeit in die Schwanzvene

Freie Universität Berlin

Handling Ratte – Transfer von Käfig zu Käfig

Transfer von Käfig zu Käfig – Ratten nicht am Schwanz hochheben!

**Material:**	**Vorbereitung:**
➔ Handschuhe	➔ Handschuhe anziehen.<br>➔ Käfig öffnen.<br>➔ Wasserflasche und Käfiggitter zu Seite stellen.<br>➔ Tiere an neue Situation gewöhnen lassen.
**Technik**	
➔ Ratte um die Schultern greifen:<br>○ Daumen unter der Schulter auf einer Seite<br>○ Restliche Finger auf der anderen Seite. Zeigefinger über der Schulter<br>➔ Ratte vorsichtig mit einer Hand hochheben und Körper mit Fingerspitzen unterstützen.<br>➔ Ratte auf Unterarm/ Brust/ geeigneter Unterlage absetzen.	

**<u>Bei sehr lebhaften Tieren:</u>**<br><br>➔ **Ratte an der Schwanzbasis zwischen Daumen, Zeige- und Mittelfinger fixieren.**<br><br>➔ **Mit der anderen Hand Ratte um die Schultern greifen:**<br><br>o **Daumen unter der Schulter auf einer Seite.**<br><br>o **Restliche Finger auf anderer Seite. Zeigefinger über der Schulter.**<br><br>➔ **Ratte vorsichtig hochheben und Körper mit Fingerspitzen unterstützen.**<br><br>➔ **Auf Unterarm/ Schulter/ geeigneter Unterlage absetzen.**	

Fixieren Ratten I – "Fixieren mit Nackengriff"

Material	Vorbereitung
➔ Handschuhe	➔ Handschuhe anziehen<br>➔ Käfig öffnen.<br>➔ Wasser und Käfigdeckel zur Seite legen.<br>➔ Ratten an neue Situation gewöhnen lassen.
**Technik**	

➔ Ratte an der Schwanzbasis mit der dominanten Hand halten.

➔ Ratte an der Schwanzbasis sehr vorsichtig(!) auf einer rauen Unterlage zurückziehen.

➔ Die Ratte mit der nicht-dominanten Hand von hinten kommend fixieren:

- Die Ratte leicht (!) nach unten drücken.
- Daumen auf einer Seite, die restlichen Finger auf der anderen Seite platzieren.
- Mit Daumen und Zeigefinger die lose Haut im Nacken direkt hinter den Ohren zu einer Hautfalte greifen.
- Anschließend mit den restlichen Fingern eine Hautfalte mit der losen Haut am greifen.

➔ Atmung kontrollieren! Brust nicht komprimieren!

➔ **Manche Ratten können bei dieser Methode „schreien“.**	

Freie Universität Berlin

Fixieren Ratten II – "über der Schulter-Griff"

Herkömmliche Methode um Ratten für Eingriffe zu fixieren

Material	Vorbereitung
➔ Handschuhe	➔ Handschuhe anziehen<br>➔ Käfig öffnen.<br>➔ Wasser und Käfigdeckel zur Seite legen.<br>➔ Ratten an neue Situation gewöhnen lassen.
**Technik**	

➔ Ratte an der Schwanzbasis mit der dominanten Hand halten.

➔ Ratte an der Schwanzbasis sehr vorsichtig(!) auf einer rauen Unterlage zurückziehen.

➔ Ratte mit der nicht-dominanten Hand von hinten kommend über den Schulterblättern greifen:

- Der Kopf liegt, so nah wie möglich an der Fingerbasis, zwischen Zeige- und Mittelfinger.
- Mit Ringfinger, kleinem Finger und Daumen Brust unterstüzen.

➔ Atmung kontrollieren! Brust nicht komprimieren!

➔ Den Körper mit der anderen Hand stabilisieren:

- Die Ratte um den Hinterleib greifen.
- Mit Daumen und Zeigefinger die Hinterbeine leicht nach unten drücken.

Freie Universität Berlin

Fixieren von Ratten III – "mittlerer Schultergriff"

Herkömmliche Methode um Ratten für Eingriffe zu fixieren

**Material**	**Vorbereitung**
➔ Handschuhe	➔ Handschuhe anziehen<br>➔ Käfig öffnen.<br>➔ Wasser und Käfigdeckel zur Seite legen.<br>➔ Ratten an neue Situation gewöhnen lassen.
**Technik**	
➔ Ratte an der Schwanzbasis mit der dominanten Hand halten.<br><br>➔ Ratte an der Schwanzbasis sehr vorsichtig(!) auf einer rauen Unterlage zurückziehen.<br><br>➔ Ratte um die Schultern greifen:<br>○ Daumen unter der Schulter auf einer Seite<br>○ Restliche Finger auf der anderen Seite. Zeigefinger über der Schulter.<br><br>➔ Daumen auf Zeigerfinger zubewegen und somit unter dem Kiefer den Kopf kontrollieren.<br><br>➔ Mit restliche Fingerspitzen die Brust unterhalb der Vorderbeine unterstützen.<br><br>➔ Atmung kontrollieren! Brust nicht komprimieren!	

➔ **Den Körper mit der anderen Hand stabilisieren:**<br><br>○ **Die Ratte um den Hinterleib greifen.**<br>○ **Mit Daumen und Zeigefinger die Hinterbeine leicht nach unten drücken.**	

Freie Universität Berlin

Fixieren Ratten IV – "unter der Schultergriff

Herkömmliche Methode um Ratten für Eingriffe zu fixieren

**Material**	**Vorbereitung**
→ Handschuhe	→ Handschuhe anziehen<br>→ Käfig öffnen.<br>→ Wasser und Käfigdeckel zur Seite legen.<br>→ Ratten an neue Situation gewöhnen lassen.
**Technik**	

→ Ratte an der Schwanzbasis mit der dominanten Hand halten.

→ Ratte an der Schwanzbasis sehr vorsichtig(!) auf einer rauen Unterlage zurückziehen.

→ Ratte mit der nicht-dominanten Hand von hinten kommend um die Schultern greifen:

- Daumen auf einer Seite, unter dem Vorderbein
- Restliche Finger auf der anderen Seite, unter dem Vorderbein.

→ Die Vorderbeine mit Daumen und Zeigefinger leicht nach vorne drücken.

→ Die Vorderbeine sollten sich vor der Brust verschränken und somit ein Beißen verhindern.

→ Atmung kontrollieren! Brust nicht komprimieren!

➔ **Den Körper mit der anderen Hand stabilisieren:**<br><br>○ **Die Ratte um den Hinterleib greifen.**<br>○ **Mit Daumen und Zeigefinger die Hinterbeine leicht nach unten drücken.**	

Ohlochmarkierung

Permanente Kennzeichnung (und Genotypisierung)

Material:	Preparation
→ Handschuhe<br>→ Ohrlochzange	→ Handschuhe anziehen.<br>→ Funktionalität der Ohrlochzange prüfen.
Technik	
→ Ratte und besonders Kopf mit nicht-dominanter Hand sicher(!) fixieren. (z.B. mit Über-der-Schulter-Griff).<br><br>→ Die flache Seite der Ohrlochzange auf das äußere Ohr legen, sodass man die Stelle, die man stanzt, sieht.<br><br>○ Ohrlochzange nur am äußeren Ohrrand anlegen und das Gefäßsystem schonen.<br><br>→ Lage kontrollieren.<br><br>→ Ohrlochzange fest zu drücken; Tier und Kopf dabei sicher(!) fixieren!<br><br>→ Ohrlochmarkierung kontrollieren.<br><br>→ Tier in den Käfig zurücksetzen.<br><br>→ Tier genau beobachten.	

Applikation intravenös in die laterale Schwanzvene

Material

- ➔ Handschuhe
- ➔ Nadel (27G)
- ➔ 1 ml Spritze
- ➔ Medium
- ➔ Warmes Wasser
- ➔ Wasserbehälter
- ➔ Abwurfcontainer
- ➔ Restrainer/ Anästhesie
- ➔ Tupfer
- ➔ Alkohohllösung o. antiseptische Lösung

Vorbereitung

- ➔ Handschuhe anziehen.

Bereits erledigt:

- ✓ 1 ml Spritze mit Medium füllen: ➔Luftblasen vermeiden.
- ✓ Wasser im Behälter erwärmen.
  - CAVE: Nicht zu stark erhitzen!
- ✓ Tier im Restrainer fixieren. / Tier anästhesieren.
- ✓ Schwanz im Wasserbad erwärmen ➔ Dilatation der Venen.
- ✓ Schwanz trocknen
- ✓ Schwanz mit Alkoholtupfer reinigen. (pro Tupfer nur einmal nach distal wischen!)

Technik

Punktion der lateralen Schwanzvene

- ➔ Den Schwanz zwischen Daumen und Zeigerfinger der nicht-dominaten Hand halten.
- ➔ Die lateralen Venen erkennen.
- ➔ Den Schwanz zwischen Daumen und Zeigefinger fixieren und die Finger als Führungslinie zur Punktion und Injektion nutzen.
- ➔ Mit der ersten Punktion im distalen Drittel des Schwanzes beginnen.
- ➔ Parallel zum Schwanz einstechen, Anschliff der Nadel zeigt nach oben.
- ➔ 2-4 mm in das Lumen der lateralen Vene einstechen.

Am lebenden Tier:

- Kein Blut im Konus sichtbar → neuer Versuch proximal der Einstichstelle.
- Vene getroffen → Blutfluss in den Ansatz des Konus → intravenöse Applikation möglich.

Intravenöse Applikation in die Schwanzvene:

- Medium langsam injizeren: max. 5 ml/kg Gewicht.
  - Injektion muss ohne Widerstand möglich sein!
- Injektionstelle kontrollieren:
  - Paravenöse Injektion: weißliche Blasen erscheinen unter der Haut → neuer Versuch proximal der Einstichstelle.
  - Intravenöse Injektion: Blut proximal der Einstichstelle weicht der injezierten Flüssigkeit.

→ Nadel entfernen, mit Tupfer auf Einstichstelle drücken um Blutung zu stoppen, Nadel im Abwurfkontainer entsorgen.

→ Injektionsstelle und Tier genau beobachten.

Blutentnahme an der lateralen Schwanzvene

Material

- ➔ Handschuhe
- ➔ Nadel (27G)
- ➔ 1ml Spritze
- ➔ Warmes Wasser
- ➔ Wasserbehälter
- ➔ Tupfer
- ➔ Alkohol-/Desinfektionslösung
- ➔ Abwurfbehälter
- ➔ Retrainer, Narkose

Vorbereitung

- ➔ Handschuhe anziehen.

Bereits erledigt

- ✓ Wasser im Behälter erwärmen.
  - CAVE: Nicht zu stark erhitzen!
- ✓ Tier im Restrainer fixieren/ narkotisieren
- ✓ Schwanz im warmen Wasserbad erwärmen-> Dilatation der Venen
- ✓ Schwanz trocknen
- ✓ Schwanz mit in Alkohol getränkten Tupfer desinfizieren
  (pro Tupfer 1x nach distal wischen.)

Methodik

Punktion der lateralen Schwanzvene

- ➔ Den Schwanz in Daumen und Zeigefinger der nicht-dominanten Hand halten.
- ➔ Verlauf der seitlichen Venen betrachten.
- ➔ Schwanz mit Zeigefinger und Daumen der nicht-dominanten Hand stabilisieren und als Führungslinie für Punktion und Applikation nutzen.
- ➔ Mit der ersten Punktion im distalen Drittel des Schwanzes beginnen.
- ➔ Die Nadel parallel zum Schwanz einstechen, Anschliff nach oben um die laterale Vene zu treffen.

Leere Spritze für Blutentnahme!

**Beim lebenden Tier:**<br><br>○ **Kein Bluteinstrom in den Nadelansatz→ nochmal versuchen.**<br><br>○ **Wenn die Vene getroffen wurde→ Bluteinfluss in den Nadelansatz→ Blutentnahme möglich.**	
**2.**	
**Blutentnahme aus der lateralen Schwanzvene**<br><br>➔ **Einschießendes Blut mittels Kappillarröhrchen oder Spritze aufnehmen.**<br>*(für den Simulator empfiehlt sich die Spritze.)*<br><br>**CAVE: Durch Aspiration kann die Vene kollabieren!**<br>**--> Sehr langsam und vorsichtig arbeiten!**<br><br>○ **Max. Volumen bei täglicher Blutentnahme: 0,7 ml/kg/Tag.**<br><br>➔ **Nadel entfernen, Tupfer auf Einstichstelle drücken, Nadel in den Abwurfbehälter.**<br><br>➔ **Einstichstelle und Tier beobachten.**	

Freie Universität Berlin
Institut für Tierschutz, Tierverhalten und Versuchstierkunde [ITTV]

Freie Universität Berlin

Training mit Simulatoren

Gruppe Simulator E

Merkmale:

- Flexible Wirbelsäule
- Applikation per os, subkutan und intramuskulär nur mit **Luft** möglich
- Applikation von Flüssigkeit in die Schwanzvene

Vorbereitendes Training für

- Handling
- Fixieren (4 Griffe)
- Applikation per os mittels Sonde
- **Subkutane Applikation in die Hautfalte (Nacken, Flanke)**
- **Intramuskuläre Applikation**
- Applikation intravenös in die laterale Schwanzvene
- Blutentnahme aus der lateralen Schwanzvene

Freie Universität Berlin

Handling Ratte – Transfer von Käfig zu Käfig

Transfer von Käfig zu Käfig – Ratten nicht am Schwanz hochheben!

**Material:**	**Vorbereitung:**
➔ Handschuhe	➔ Handschuhe anziehen.<br>➔ Käfig öffnen.<br>➔ Wasserflasche und Käfiggitter zu Seite stellen.<br>➔ Tiere an neue Situation gewöhnen lassen.
**Technik**	
➔ Ratte um die Schultern greifen:<br><br>○ Daumen unter der Schulter auf einer Seite<br><br>○ Restliche Finger auf der anderen Seite. Zeigefinger über der Schulter<br><br>➔ Ratte vorsichtig mit einer Hand hochheben und Körper mit Fingerspitzen unterstützen.<br><br>➔ Ratte auf Unterarm/ Brust/ geeigneter Unterlage absetzen.	

**<u>Bei sehr lebhaften Tieren:</u>**<br><br>➔ **Ratte an der Schwanzbasis zwischen Daumen, Zeige- und Mittelfinger fixieren.**<br><br>➔ **Mit der anderen Hand Ratte um die Schultern greifen:**<br><br>o **Daumen unter der Schulter auf einer Seite.**<br><br>o **Restliche Finger auf anderer Seite. Zeigefinger über der Schulter.**<br><br>➔ **Ratte vorsichtig hochheben und Körper mit Fingerspitzen unterstützen.**<br><br>➔ **Auf Unterarm/ Schulter/ geeigneter Unterlage absetzen.**	

Fixieren Ratten I – “Fixieren mit Nackengriff“

Material
➔ Handschuhe

Vorbereitung
➔ Handschuhe anziehen
➔ Käfig öffnen.
➔ Wasser und Käfigdeckel zur Seite legen.
➔ Ratten an neue Situation gewöhnen lassen.

Technik

➔ Ratte an der Schwanzbasis mit der dominanten Hand halten.

➔ Ratte an der Schwanzbasis sehr vorsichtig(!) auf einer rauen Unterlage zurückziehen.

➔ Die Ratte mit der nicht-dominanten Hand von hinten kommend fixieren:

- Die Ratte leicht (!) nach unten drücken.
- Daumen auf einer Seite, die restlichen Finger auf der anderen Seite platzieren.
- Mit Daumen und Zeigefinger die lose Haut im Nacken direkt hinter den Ohren zu einer Hautfalte greifen.
- Anschließend mit den restlichen Fingern eine Hautfalte mit der losen Haut am greifen.

➔ Atmung kontrollieren! Brust nicht komprimieren!

➔ **Manche Ratten können bei dieser Methode „schreien“.**	

Fixieren Ratten II – "über der Schulter-Griff"

Herkömmliche Methode um Ratten für Eingriffe zu fixieren

<table>
<tr><td><u>Material</u>
➔ Handschuhe</td><td><u>Vorbereitung</u>
➔ Handschuhe anziehen
➔ Käfig öffnen.
➔ Wasser und Käfigdeckel zur Seite legen.
➔ Ratten an neue Situation gewöhnen lassen.</td></tr>
<tr><td><u>Technik</u></td><td></td></tr>
<tr><td>➔ Ratte an der Schwanzbasis mit der dominanten Hand halten.

➔ Ratte an der Schwanzbasis sehr vorsichtig(!) auf einer rauen Unterlage zurückziehen.

➔ Ratte mit der nicht-dominanten Hand von hinten kommend über den Schulterblättern greifen:

- Der Kopf liegt, so nah wie möglich an der Fingerbasis, zwischen Zeige- und Mittelfinger.

– Mit Ringfinger, kleinem Finger und Daumen Brust unterstützen.

➔ Atmung kontrollieren! Brust nicht komprimieren!

➔ Den Körper mit der anderen Hand stabilisieren:

o Die Ratte um den Hinterleib greifen.

o Mit Daumen und Zeigefinger die Hinterbeine leicht nach unten drücken.</td><td></td></tr>
</table>

Freie Universität Berlin

Fixieren von Ratten III – “mittlerer Schultergriff“

Herkömmliche Methode um Ratten für Eingriffe zu fixieren

Material	Vorbereitung
➔ Handschuhe	➔ Handschuhe anziehen<br>➔ Käfig öffnen.<br>➔ Wasser und Käfigdeckel zur Seite legen.<br>➔ Ratten an neue Situation gewöhnen lassen.

Technik

➔ Ratte an der Schwanzbasis mit der dominanten Hand halten.

➔ Ratte an der Schwanzbasis sehr vorsichtig(!) auf einer rauen Unterlage zurückziehen.

➔ Ratte um die Schultern greifen:

- Daumen unter der Schulter auf einer Seite

- Restliche Finger auf der anderen Seite. Zeigefinger über der Schulter.

➔ Daumen auf Zeigerfinger zubewegen und somit unter dem Kiefer den Kopf kontrollieren.

➔ Mit restliche Fingerspitzen die Brust unterhalb der Vorderbeine unterstützen.

➔ Atmung kontrollieren! Brust nicht komprimieren!

➔ **Den Körper mit der anderen Hand stabilisieren:**<br><br>○ **Die Ratte um den Hinterleib greifen.**<br>○ **Mit Daumen und Zeigefinger die Hinterbeine leicht nach unten drücken.**	

Freie Universität Berlin

Fixieren Ratten IV – “unter der Schultergriff“

Herkömmliche Methode um Ratten für Eingriffe zu fixieren

**Material**	**Vorbereitung**
➔ Handschuhe	➔ Handschuhe anziehen<br>➔ Käfig öffnen.<br>➔ Wasser und Käfigdeckel zur Seite legen.<br>➔ Ratten an neue Situation gewöhnen lassen.
**Technik**	

➔ Ratte an der Schwanzbasis mit der dominanten Hand halten.

➔ Ratte an der Schwanzbasis sehr vorsichtig(!) auf einer rauen Unterlage zurückziehen.

➔ Ratte mit der nicht-dominanten Hand von hinten kommend um die Schultern greifen:

- Daumen auf einer Seite, unter dem Vorderbein
- Restliche Finger auf der anderen Seite, unter dem Vorderbein.

➔ Die Vorderbeine mit Daumen und Zeigerfinger leicht nach vorne drücken.

➔ Die Vorderbeine sollten sich vor der Brust verschränken und somit ein Beißen verhindern.

➔ Atmung kontrollieren! Brust nicht komprimieren!

➔ **Den Körper mit der anderen Hand stabilisieren:**<br><br>◦ **Die Ratte um den Hinterleib greifen.**<br>◦ **Mit Daumen und Zeigefinger die Hinterbeine leicht nach unten drücken.**	

Applikation *per os* mittels Schlundsonde

Enterale Applikation

Material	Vorbereitung
→ Handschuhe<br>→ Schlundsonde (20-22 G x2-inch)<br>→ 1 ml Spritze<br>→ Medium (für Simulator E *nur* *Luft!*)<br>→ (Marker)	→ Handschuhe anziehen<br>Bereits erledigt<br>✓ Medium in Spritze aufziehen.
Methodik	
**Richtige Sondenlänge abmessen**<br><br>→ Tier sicher mit Nackengriff fixieren.<br>○ Richtige Länge = Abstand zwischen Nasenspitze und Rippenbogen.<br><br>→ Abstand falls nötig markieren.<br>○ (CAVE! Chemische Zusammensetzung Marker!)<br><br>**Einführen der Schlundsonde**<br><br>→ Tier sicher (!) in vertikaler Position fixieren.<br><br>→ Sonde neben Schneidezähnen, am oberen Gaumen entlang, Richtung Kehlkopf vorführen.<br>(echtes Tier: Schluckreflex)<br><br>→ Den Kopf des Tieres vorsichtig gerade richten→ Sonde gleitet von selbst in die Speiseröhre.<br><br>→ Schlundsonde bis in den Magen vorführen.<br>○ Die Sonde gleitet fast von selbst den Ösophagus entlang.	

CAVE!

➔ **Niemals Gewalt anwenden!**

➔ **Bei Widerstand: Stop! Sonde entfernen, erneut versuchen!**

➔ **Atmung immer kontrollieren!**

➔ **Bei unregelmäßiger Atmung:**

- **Eingriff sofort stoppen!**
- **Tier in den Käfig zurücksetzen und erneut versuchen, wenn sich das Tier erholt hat.**

Bolusgabe und Entfernen der Sonde

Bei regelmäßiger Atmung und Lage im Magen:

➔ **Eingabe von Medium (max. 10 ml/kg) langsam und stetig.**

➔ **Sonde dem Verlauf folgend entfernen.**

➔ **Das Tier vorsichtig in den Käfig zurücksetzen.**

➔ **Tier und Atmung beobachten.**

Freie Universität Berlin

Subkutane Applikation

**Material:**	**Vorbereitung:**
➔ Handschuhe<br>➔ Nadel (26G)<br>➔ 1ml Spritze<br>➔ Medium (für Simulator E *bitte nur Luft verwenden!)*	➔ Handschuhe anziehen.<br>Bereits erledigt./nicht nötig für Simulator E:<br>✓ Medium in 1ml Spritze aufziehen:<br>○ Luftblasen vermeiden.
**Technik**	
Subkutane Applikation in die Nackenhautfalte<br><br>➔ Ratte mit Nackengriff fixieren.<br><br>➔ Nadel an der Basis der Nackenhautfalte parallel zur Körperachse einstechen.<br><br>➔ Vorsichtig aspirieren:<br>○ Subkutane Lage: Kein Blutrückfluss in den Nadelansatz. ➔<br>○ Parallel zwischen den fixierenden Fingern in die Hautfalte injizieren. CAVE: Nicht in die eigenen Finger injizieren!<br><br>➔ Max. Applikationsvolumen: 10 ml/ kg. !!!!! In die Simulator E bitte nur Luft injizieren!!!!!!<br><br>➔ Nadel entfernen und im Abwurfkontainer entsorgen.<br><br>➔ Injektionsstelle und Tier genau beobachten.	

Applikation in die seitliche Bauchfalte (Flanke)	
✓ Hilfsperson organisieren:<br>○ Tier mit Schultergriff fixieren.<br>○ Hinterleib und Hinterbeine mit der anderen Hand fixieren.<br><br>➔ Mit Daumen, Zeige- und Mittelfinger der nicht-dominanten Hand eine Hautfalte an der seitlichen Bauchwand über den Hinterbeinen (Bereich der Flanke) greifen.<br><br>➔ Nadel an der Basis der Hautfalte im 30-45° Winkel einstechen.<br><br>➔ Vorsichtig aspirieren:<br>○ Subkutane Lage: Kein Blutrückfluss im Nadelansatz. ➔<br><br>➔ Parallel zwischen den fixierenden Fingern in die Hautfalte injizieren.<br>○ CAVE: Nicht in die eigenen Finger injizieren!<br><br>➔ Max. Applikationsvolumen: 10 ml/ kg. !!!!! In die Simulator E bitte nur Luft injizieren!!!!!!<br><br>➔ Nadel entfernen und im Abwurfkontainer entsorgen.<br><br>➔ Injektionsstelle und Tier genau beobachten.	

Freie Universität Berlin

Intramuskuläre Administration

Anwendung nur wenn andere Applikationswege nicht möglich. Potentiell schmerzhaft.

Material

- ➔ Handschuhe
- ➔ Nadel (26G)
- ➔ 1 ml Spritze
- ➔ Medium (*für Simulator E bitte nur Luft benutzen!*)

Vorbereitung

- ➔ Handschuhe anziehen.
- ➔ Hilfsperson organisieren.

Bereits erledigt. /Nicht nötig für Simulator E.

- ✓ Medium in 1 ml Spritze aufziehen.

Technik

➔ **Hilfsperson:**

- Ratte mit Schultergriff fixieren.
- Hinterleib und ein Hinterbein mit anderer Hand festhalten.

➔ **Zweite Person:**

- Anderes Hinterbein vorsichtig strecken und fixieren.
- Bein mit drei Fingern stabilisieren. (siehe Foto).
- Einstich in den kaudalen Oberschenkelmuskel
  - CAVE: Nervus ischiadicus und fermorale Blutgefäße schonen!

➔ Leicht aspirieren:

  - Intramuskuläre Lage: Kein Blutrückfluss in den Nadelansatz.

➔ **Langsam und vorsichtig injizieren:**<br><br>○ **Max. Applikationsvolumen 0,1 – 0,2 ml pro Injektion.**<br>***(<u>für Simulator E bitte nur Luft verwenden!</u>)***<br><br>➔ **Nadel entfernen und im Abwurfkontainer entsorgen.**<br><br>➔ **Injektionstelle und Tier genau beobachten.**	

Freie Universität Berlin

Applikation intravenös in die laterale Schwanzvene

Material	Vorbereitung
→ Handschuhe<br>→ Nadel (27G)<br>→ 1 ml Spritze<br>→ Medium<br>→ Warmes Wasser<br>→ Wasserbehälter<br>→ Abwurfcontainer<br>→ Restrainer/ Anästhesie<br>→ Tupfer<br>→ Alkohohllösung o. antiseptische Lösung	→ Handschuhe anziehen.<br>Bereits erledigt:<br>✓ 1 ml Spritze mit Medium füllen: →Luftblasen vermeiden.<br>✓ Wasser im Behälter erwärmen.<br>o CAVE: Nicht zu stark erhitzen!<br>✓ Tier im Restrainer fixieren. / Tier anästhesieren.<br>✓ Schwanz im Wasserbad erwärmen → Dilatation der Venen.<br>✓ Schwanz trocknen<br>✓ Schwanz mit Alkoholtupfer reinigen. (pro Tupfer nur einmal nach distal wischen!)
**Technik**	
**Punktion der lateralen Schwanzvene**<br><br>→ Den Schwanz zwischen Daumen und Zeigerfinger der nicht-dominaten Hand halten.<br><br>→ Die lateralen Venen erkennen.<br><br>→ Den Schwanz zwischen Daumen und Zeigefinger fixieren und die Finger als Führungslinie zur Punktion und Injektion nutzen.<br><br>→ Mit der ersten Punktion im distalen Drittel des Schwanzes beginnen.<br><br>→ Parallel zum Schwanz einstechen, Anschliff der Nadel zeigt nach oben.<br><br>→ 2-4 mm in das Lumen der lateralen Vene einstechen.	

Am lebenden Tier:

- Kein Blut im Konus sichtbar → neuer Versuch proximal der Einstichstelle.

- Vene getroffen → Blutfluss in den Ansatz des Konus → intravenöse Applikation möglich.

Intravenöse Applikation in die Schwanzvene:

- Medium langsam injizeren: max. 5 ml/kg Gewicht.

  - Injektion muss ohne Widerstand möglich sein!

- Injektionstelle kontrollieren:

  - Paravenöse Injektion: weißliche Blasen erscheinen unter der Haut → neuer Versuch proximal der Einstichstelle.

  - Intravenöse Injektion:
    Blut proximal der Einstichstelle weicht der injezierten Flüssigkeit.

→ Nadel entfernen, mit Tupfer auf Einstichstelle drücken um Blutung zu stoppen, Nadel im Abwurfkontainer entsorgen.

→ Injektionsstelle und Tier genau beobachten.

Freie Universität Berlin

Blutentnahme an der lateralen Schwanzvene

Material

- ➔ Handschuhe
- ➔ Nadel (27G)
- ➔ 1ml Spritze
- ➔ Warmes Wasser
- ➔ Wasserbehälter
- ➔ Tupfer
- ➔ Alkohol-/Desinfektionslösung
- ➔ Abwurfbehälter
- ➔ Retrainer, Narkose

Vorbereitung

- ➔ Handschuhe anziehen.

Bereits erledigt

- ✓ Wasser im Behälter erwärmen.
  - CAVE: Nicht zu stark erhitzen!
- ✓ Tier im Restrainer fixieren/ narkotisieren
- ✓ Schwanz im warmen Wasserbad erwärmen-> Dilatation der Venen
- ✓ Schwanz trocknen
- ✓ Schwanz mit in Alkohol getränkten Tupfer desinfizieren (pro Tupfer 1x nach distal wischen.)

Methodik

Punktion der lateralen Schwanzvene

- ➔ Den Schwanz in Daumen und Zeigefinger der nicht-dominanten Hand halten.
- ➔ Verlauf der seitlichen Venen betrachten.
- ➔ Schwanz mit Zeigefinger und Daumen der nicht-dominanten Hand stabilisieren und als Führungslinie für Punktion und Applikation nutzen.
- ➔ Mit der ersten Punktion im distalen Drittel des Schwanzes beginnen.
- ➔ Die Nadel parallel zum Schwanz einstechen, Anschliff nach oben um die laterale Vene zu treffen.

Leere Spritze für Blutentnahme!

Beim lebenden Tier:

- **Kein Bluteinstrom in den Nadelansatz→ nochmal versuchen.**

- **Wenn die Vene getroffen wurde→ Bluteinfluss in den Nadelansatz→ Blutentnahme möglich.**

2.

Blutentnahme aus der lateralen Schwanzvene

➔ **Einschießendes Blut mittels Kappillarröhrchen oder Spritze aufnehmen.**
(für den Simulator empfiehlt sich die Spritze.)

CAVE: Durch Aspiration kann die Vene kollabieren!
--> Sehr langsam und vorsichtig arbeiten!

- **Max. Volumen bei täglicher Blutentnahme: 0,7 ml/kg/Tag.**

➔ **Nadel entfernen, Tupfer auf Einstichstelle drücken, Nadel in den Abwurfbehälter.**

➔ **Einstichstelle und Tier beobachten.**
